# Understanding the role of TNFR2 signaling in the tumor microenvironment of breast cancer

**DOI:** 10.1186/s13046-024-03218-1

**Published:** 2024-11-28

**Authors:** Ali Mussa, Nor Hayati Ismail, Mahasin Hamid, Mohammad A. I. Al-Hatamleh, Anthony Bragoli, Khalid Hajissa, Noor Fatmawati Mokhtar, Rohimah Mohamud, Vuk Uskoković, Rosline Hassan

**Affiliations:** 1 Department of Haematology, School of Medical Sciences, Universiti Sains Malaysia, Kubang Kerian, Kota Bharu , Kelantan, 16150 Malaysia; 2 Department of Biology, Faculty of Education, Omdurman Islamic University, P.O. Box 382, Omdurman, Sudan; 3 Department of Pharmaceutics, Xiangya School of Pharmaceutical Sciences, Central South University, Hunan Province, Changsha, 410013 China; 4 Department of Zoology, Faculty of Sciences and Information Technology, University of Nyala, Nyala, 63311 Sudan; 5 Division of Hematology and Oncology, Department of Medicine, UPMC Hillman Cancer Center, University of Pittsburgh, Pittsburgh, PA 15213 USA; 6 Department of Immunology, University of Pittsburgh, Pittsburgh, PA 15260 USA; 7 Department of Zoology, Faculty of Science and Technology, Omdurman Islamic University, P.O. Box 382, Omdurman, Sudan; 8 Institute for Research in Molecular Medicine (iNFORMM), Universiti Sains Malaysia, Kubang Kerian, Kota Bharu , Kelantan, 16150 Malaysia; 9 Department of Immunology, School of Medical Sciences, Universiti Sains Malaysia, Kubang Kerian, Kota Bharu , Kelantan, 16150 Malaysia; 10 TardigradeNano LLC, Irvine, CA 92604 USA; 11 Division of Natural Sciences, Fullerton College, Fullerton, CA 92832 USA

**Keywords:** Immune checkpoint, Immunosuppressive TME, TNF, TNFRSF1B, CD120b

## Abstract

Breast cancer (BC) is the most frequently diagnosed malignancy among women. It is characterized by a high level of heterogeneity that emerges from the interaction of several cellular and soluble components in the tumor microenvironment (TME), such as cytokines, tumor cells and tumor-associated immune cells. Tumor necrosis factor (TNF) receptor 2 (TNFR2) appears to play a significant role in microenvironmental regulation, tumor progression, immune evasion, drug resistance, and metastasis of many types of cancer, including BC. However, the significance of TNFR2 in BC biology is not fully understood. This review provides an overview of TNFR2 biology, detailing its activation and its interactions with important signaling pathways in the TME (e.g., NF-κB, MAPK, and PI3K/Akt pathways). We discuss potential therapeutic strategies targeting TNFR2, with the aim of enhancing the antitumor immune response to BC. This review provides insights into role of TNFR2 as a major immune checkpoint for the future treatment of patients with BC.

## Introduction

Breast cancer (BC) is the most frequently diagnosed malignancy among women, with an expected 2,261,419 (11.7%) reported incidents and 684,996 (6.9%) new fatalities each year [1]. This cancer is characterized by a very complex and heterogeneous microenvironment with distinct molecular and histological characteristics, therapeutic responses, and clinical prognosis [2]. This high level of heterogeneity is reflected by the expression of three prime genes—estrogen receptor (ER), progesterone receptor (PR), and human epidermal growth factor receptor 2 (HER2) — which are primarily utilized for diagnosis and therapy selection. BC is classified as hormone receptor (HR)-positive (HR^+^/HER2^−^, or ER^+^/PR^+^/HER2^−^), triple positive (HR^+^/HER2^+^, or ER^+^/PR^+^/HER2^+^), HER2-positive (ER^−^/PR^−^/HER2^+^, or HER2^+^), HER2-negative (ER^+^/PR^+^/HER2^−^, or HER2^−^), or triple-negative (TNBC) (ER^−^/PR^−^/HER2^−^). Furthermore, based on phenotypic and genetic categorization BC has been separated into four different subgroups: 1) luminal A (ER^+^/PR^+^/HER2^−^, low proliferation factor Ki67^+^ (14%), low grade); 2) luminal B (ER^+^/PR^+^/HER2^+^, high proliferation factor Ki67^+^ (14%), high grade); 3) HER2-enriched (ER^−^/PR^−^/HER2^+^, any Ki67 level, strong proliferation); 4) basal-like (ER^−^/PR^−^/HER2^−^, high grade and proliferation, necrosis) [3, 4]. All of these groupings have been linked to a worse prognosis and outcome for patients with BC.

The tumor microenvironment (TME) is currently known to play a significant role in the initiation and progression of tumors, serving also as a quantitative indicator of therapy efficacy [5]. The complexity of TME in BC is characterized by the aberrant expression of various immunoinhibitory receptors/ligands, cytokines, chemokines, and several cellular components that support tumor growth. Most notably, among the receptors, the aberrant expression of tumor necrosis factor (TNF) receptor 2 (TNFR2, TNFRSF1B) on the TME cells activates several signaling pathways after the interaction with its ligand, TNF, that support the tumor growth [6, 7]. Recent findings suggest that the expression of TNFR2 is associated with the immunosuppression, progression, and development of certain tumors, including BC [8, 9]. However, the significance of TNFR2 in BC biology is not fully understood. This review discusses TNFR2, including its activation, its interactions with various critical signaling pathways, its immunosuppressive cell activation, and potential targeted TNFR2 therapies in the context of BC TME.

## The breast cancer tumor microenvironment

The TME of BC is a complex network of diverse specialized cells that contributes significantly to its development and progression [10, 11]. It typically consists of tumor cells, immune cells and stroma, which together behave in a highly coordinated manner. In the process of cancer development, the TME experiences continuous dynamic changes [10, 11]. The major cellular components of the TME in addition to BC cells and breast cancer stem cells (BCSCs) encompass CD8^+^ T cells, CD4^+^ T cells, regulatory T cells (T-regs), B cells, macrophages, neutrophils, eosinophils, mast cells, dendritic cells (DCs), myeloid derived suppressor cells (MDSCs), and natural killer (NK) cells, thus including both adaptive and innate immune cells [12–14]. The TME stroma comprises various stromal cells, including adipocytes, cancer-associated fibroblasts (CAFs), endothelial cells (ECs), mesenchymal stem cells (MSCs), blood cells, and pericytes. In addition, the TME comprises not only various tissue-specific resident cells, but also includes non-cellular components such as the extracellular matrix (ECM) [12, 13, 15]. The considerable diversity of the TME presents substantial challenges for the development of novel effective treatments for BC; therefore, efforts are still ongoing to address this issue.

**Fig. 1:**
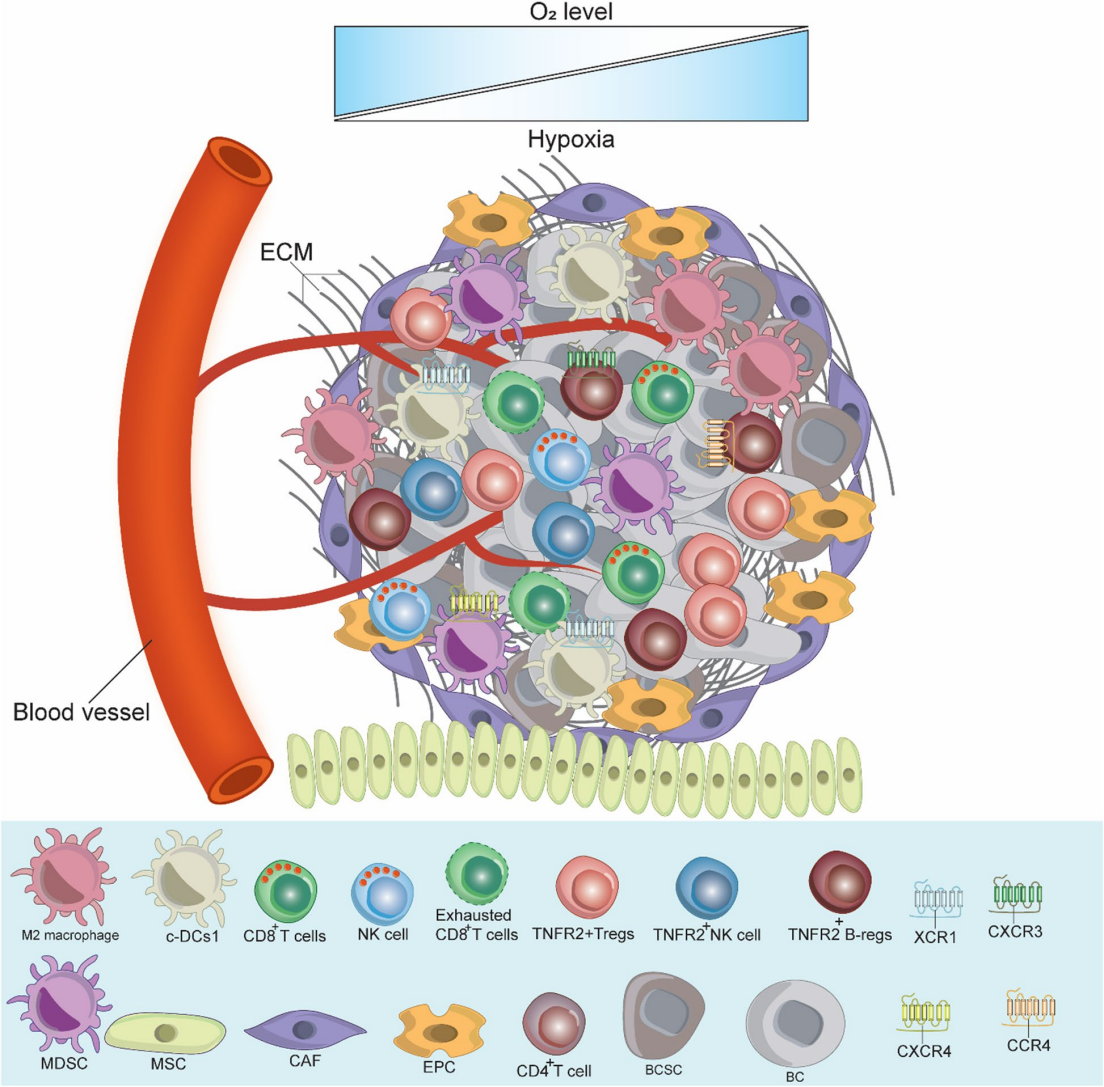
Breast Cancer Tumor Microenvironment. As a solid tumor, the BC TME is highly complex and contains the ECM and various immune cells in addition to BC cells and BCSCs. These cells interact with each other, express, and release several immunomodulators to stimulate BC therapy resistance and escape from the anti-tumor immune response, which in turn promotes BC cell growth, invasion, and metastasis. c-DC1, type 1 conventional DCs; BC, breast cancer; BCSCs, breast cancer stem cells; MDSCs, myeloid-derived suppressor cells; T-regs, T regulatory cells; B-regs, B regulatory cells, CAFs, cancer associated fibroblast; EPCs, endothelial; MSCs, mesenchymal stem cells; TME, tumor microenvironment; ECM, extracellular matrix; CXCR3, C-X-C chemokine receptor 3; CXCR4, C-X-C chemokine receptor 4; XCR1, X-C motif chemokine receptor 1

The cells of the BC TME express various functional receptors, such as PD-1, PD-L1, CTLA-4, TNFR2, and chemokine receptors (e.g., XCR1, CXCR3, CXCR-4, and CCR4). These cells also release various cellular mediators, including cytokines (e.g., TNF-α, IL-1β, IL-4, IL-6, IL-8, IL-10, and TGF-β), as well we as expressing various chemokines (e.g., CXCL-12, CCL8, and CXCL-9), and other molecules like PEG_2_, IDO, COX-2, VEGF, MMPs, iNOS, and ARG-1, which collectively favor BC development, angiogenesis, growth, metastasis, drug resistance and immune response escape [10, 12, 15–17]. These mediators and molecules induce their function via autocrine and paracrine loops, coordinating effectively all the components of BC TME and activating various signaling pathways, such as the EGFR, JAK/STAT3, MAPK, PI3K/Akt and NF-κB [12]. Hypoxia, which can be generated due to the nature of BC as a solid tumor, also effects BC TME by activating HIF-1α, subsequently stimulating various signaling pathways [15]. The intricate interaction between all the components of BC TME requires the development of a targeted therapy that would inhibit or reduce the expression of the immunoinhibitory receptors such as PD-1/PD-L1, CTLA-4 and TNFR2, which control and regulate various signaling pathways. Figure 1 describes BC TME, showing the diverse cells involved in mutual interaction favoring BC drug resistance, immune escape, development, invasion, and metastasis.

## The molecular biology of TNFR2

### The genomic organization of the TNFRSF1B gene

**Fig. 2:**
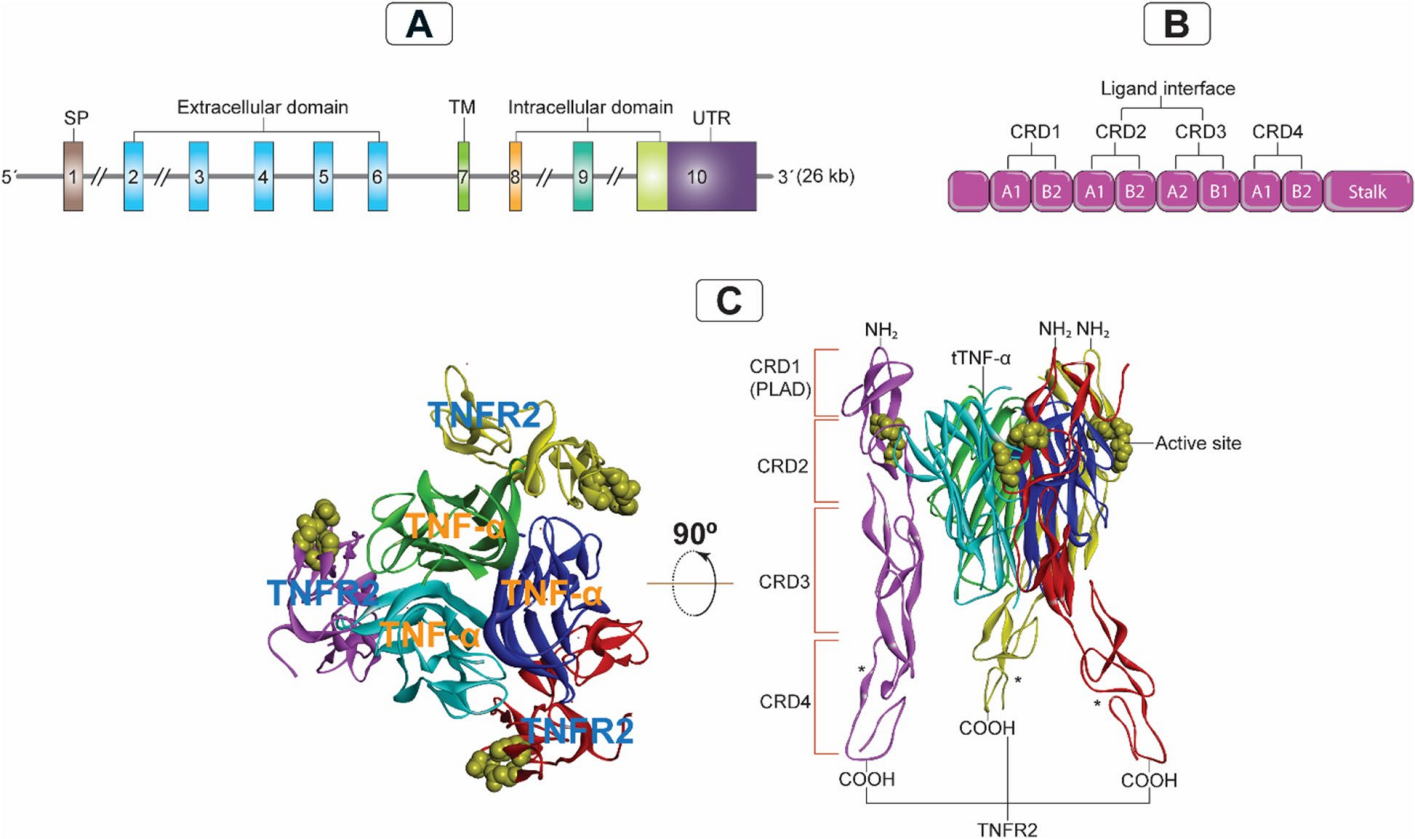
Genomic organization of TNFR2 and its interaction with TNF-α. A: The *TNFRSF1B* gene organization on chromosome 1p36.2, consisting of 10 exons ranging from 35 to 2489 bp and 9 introns ranging from 338 to 7500 bp, totals 26 kb. B: Schematic representation of CRD 1–4 of TNFR2 indicating the component of each CRD of the two modules (A1, A2, B1 or B2), CRD 1 (PLAD; A1 and B2), CRD 2 (A1 and B2), CRD 3 (A2 and B1), and CRD 4 (A1 and B2). C: Showing the crystal structure of the 3ALQ (Protein Data Bank ID) of the TNFR2/TNF-α complex, indicating the confirmational interaction between three units of TNFR2 and three units of TNF-α. TNFR2, tumor necrosis factor receptor type 2; tTNF-α, triple tumor necrosis factor; TM, transmembrane; UTR, untranslated region; CRD, cysteine-rich domain; PLAD, pre-ligand assembly domain; kb, kilobases; bp, base pairs; (*), three identical TNFR2 units

Decades ago, considerable efforts were made to unravel the genetic, structural, and mechanistic importance of human TNFR1 and TNFR2. In this respect, the *TNFRSF1A* and *TNF-α* genes are located on chromosomes 12p13, and 6p21.3 respectively, while the *TNFRSF1B* gene is situated on chromosome 1p36.2 and consists of 10 exons ranging from 35 to 2489 base pairs (bp) and 9 introns ranging from 338 to 7500 bp, totaling 26 kilobases (kb) (Fig. 2A)[18, 19]. Structurally, exon 1 comprises the N-terminal sequences and the peptide chain important for the signaling transfer, whereas exons 2–6 include the cysteine-rich extracellular domain. Exons 8–10 encode the intracellular domain, while exon 7 encodes the trans-membrane (TM) region, which overlaps with a minor portion of exon 6 [18]. Remarkably, the functional domains are separated by *TNFRSF1B* gene splice sites, allowing each domain to be expressed as a distinct exon. Both *TNFRSF1A* and *TNFRSF1B* have the same number of exons and are comparable in size, indicating a high degree of structural similarity between the two genes. Also, the splice sites in both genes operate in the same manner to partition the functional domains of each gene [18]. Based on these findings, it can be inferred that there is a common association between the functional organization of the TNFR2 protein and the genomic architecture of its encoding gene.

### TNFR2 structure, expression and interaction with TNF-α

**Fig. 3:**
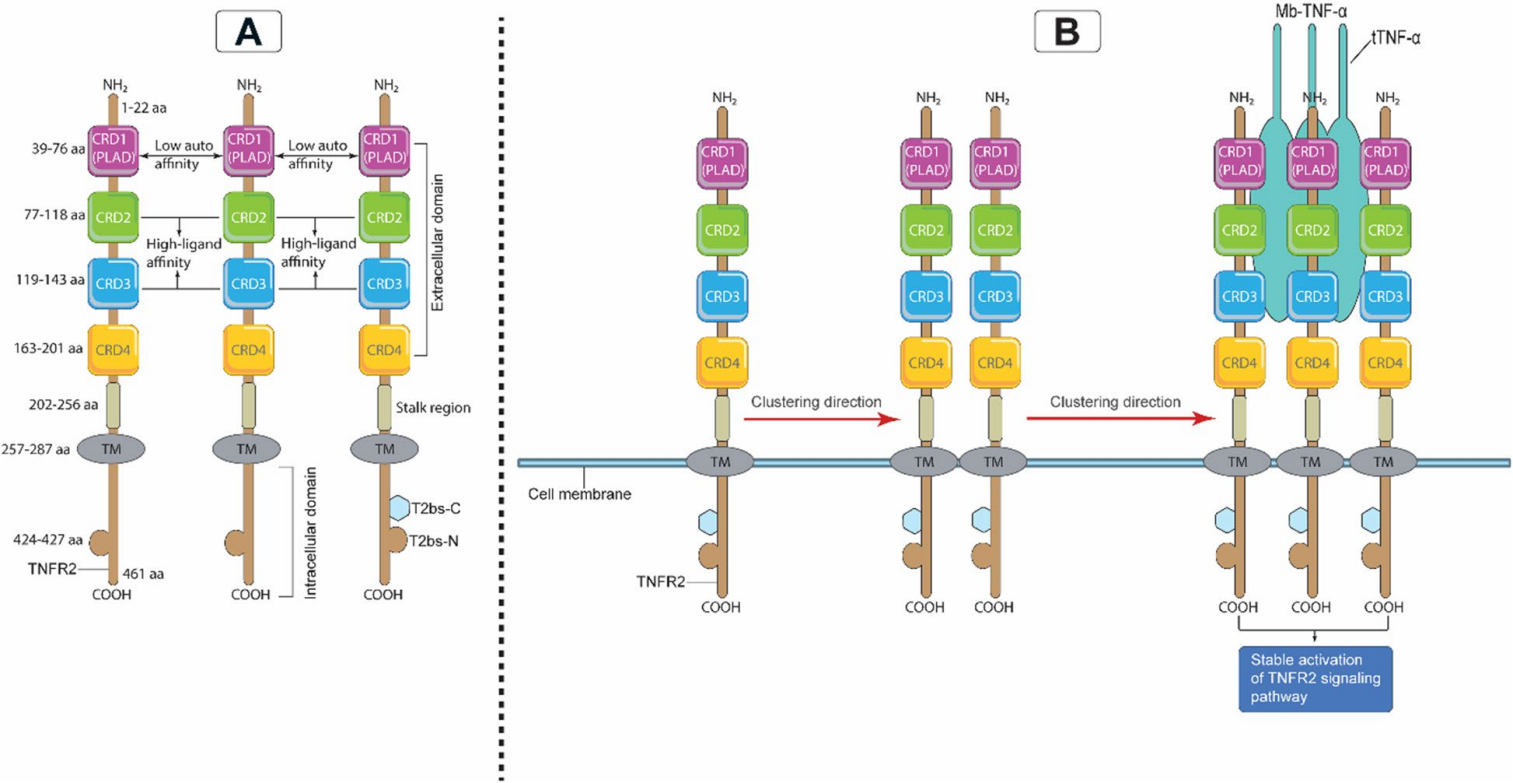
TNFR2 protein structure and assembly on the cell surface. A. The full amino acid protein structure of the TNFR2 extracellular and intracellular domains. In the extracellular amino N-terminus domain, the organization of each CRD (1–4) is indicated, with the CRD1 harboring the self-structure interacting domain PLAD that interacts with low affinity with TNF-α and the regions of the CRD2 and CRD3 interacting with strong affinity with TNF-α. In the intracellular C-terminus domain, the receptor contains the TRAF binding sites (T2bs-C and T2bs-N) that actively recruit TRAF2 to inhibit or initiate signaling. B. A single chain of TNFR2 will start clustering with a second TNFR2 chain via their PLADs, then the two chains will cluster with a third chain via PLAD-PLAD interactions; then a Mb-TNF-α trimer binds to these clustering chains of TNFR2 to form a fully stable and active signalosome. CRD, cysteine-rich domain; PLAD, pre-ligand assembly domain; TNFR2, tumor necrosis receptor type two; Mb-TNF-α, membrane-bound tumor necrosis factor; TRAF, TNF receptor associated factor; T2bs-C, TRAF2-binding site C; T2bs-N, TRAF2-binding site N

TNFR2 is an approximately 75-kDa glycoprotein that mediates its function upon TNF-α binding (Fig. 3A). Although they share similar extracellular TNF-α-binding regions, TNFR2 is structurally distinct from TNFR1, another TNF-α receptor in the same superfamily [18]. TNFR1 and TNFR2 are type I transmembrane proteins belonging to the members of the TNFR1-associated death domain (TRADD) and TNFR-associated factors (TRAFs), respectively. Typically, the superfamily contains an extracellular domain composed of multiple cysteine-rich domains (CRDs) organized in a long structure and containing three disulfide linkages between six cysteine residues [20, 21]. Indeed, both TNFR1 and TNFR2 include four CRDs (CRD1-CRD4), of which CRD1—pre-ligand assembly domain (PLAD)—controls the formation of the TNFR self-assembly on the cellular membrane [22]. CRD2 and CRD3 enable the binding of TNF-α to TNFRs. However, the function of the membrane-proximal CRD4 is still elusive [23]. Here, it should be noted that TNFR1 and TNFR2 are membrane-bound receptors and can sometimes be enzymatically cleaved to their soluble forms.

In fact, the signal that is delivered when membrane-bound TNF-α (Mb-TNF-α, 34-kDa) binds to TNFR2 is stronger and mediates more functions than the signal that is transmitted by soluble TNF-α (s-TNF-α, 17-kDa) [19, 23]. Indeed, both TNF-α forms feature a CRD-interacting component known as the TNF-α homology domain (THD), which is responsible for TNF-α binding to the CRD regions of TNFR2 [21]. Structurally, the THD comprises 150 amino acid residues that are conserved, aromatic, and hydrophobic, in nature. Its conformational structure is a β-sandwich consisting of 10 anti-parallel β strands that form two stacked β-plated sheets, ultimately resulting in a jelly-roll structure [21]. Similarly, the trimeric contact involves the A, A', H, C, and F strands of the inner sheet, with the B, B', D, E, and G strands of the outer sheet protruding outward. The trimeric structure of THD has a bell-shaped form of approximately 60 angstroms in height. It comprises a conserved anti-parallel β-strand compact core with variable loops extending from it [21].

When the sequence structures of TNFR2 and TNFR1 were analyzed, topological and structural similarities in both receptors' CRD1 and CRD2 were discovered [20]. Unexpectedly, CRDs have two modules: module A1, which has a single disulfide bond, and module B2, which has two disulfides. However, CRD3 of TNFR2 contains type A2 module with 1–4, and 2–3 disulfide bridges (Fig. 2B) [20]. Notably, the binding of TNF-α to TNFR2 necessitates a strong interaction between various component-mediated clusters to initiate an effective signaling complex. In order to do this, TNF-α creates a core homotrimer for the three TNFR2 subunits to attach to. More specifically, regions 3 and 4 make up the center of the contact between TNFR2 and TNF-α [20]. It was discovered that Region 3 included an A1 module of CRD2, but Region 4 contained the B2 module of CRD2 and the A2 module of CRD3, which were adjacent to regions 1 and 2 (Fig. 2B). In TNFR2 region 3, a sequence of acidic amino acid residues (Asp54, Glu57, and Glu70) forms one group, generating a negatively charged surface [20]. Region 4 of TNFR2 comprises the basic amino acid residues Arg77, Lys108, and Arg133, which ultimately results in a positively charged surface. Region 3 of TNFR2 mediates binding through interactions with the basic Arg31 of TNF-α (Figs. 2C and 3B) [20]. Furthermore, Arg32, Asp143, Gln149, and Glu23 of TNF-α establish hydrogen bonds with Ser73, Arg113, and Arg77 of TNFR2 to increase the binding stability (Figs. 2C and 3B) [20]. This evidence suggests that regions 3 and 4 play a crucial role in the maintenance of an effective and firm signaling flow generated by the TNF-α/TNFR2 signalosome complex (Fig. 2B). Moreover, these interactions are essential for the stability and functionality of the TNF-α-TNFR2 signaling complex. This could have important implications for the comprehension and targeting of inflammatory immune responses and associated diseases.

Another equally important factor is the PLAD of the CRD1. At the cell surface, TNFR2 subunits may communicate with one another via PLAD-PLAD interactions (Fig. 3B). TNF-α trimers have the ability to bind around the TNFR2 trimer (Fig. 3B). A two-dimensional TNF-α/TNFR2 network might be created as a consequence of the binding of TNF-α to TNFR2. This may connect it to other TNFR2 trimers via various attachment domains [20, 22, 23]. In order for the TNF-α/TNFR2 signaling pathway to be fully activated, trimeric Mb-TNF-α needs to interact with trimeric membrane-bound TNFR2(Fig. 3B) [24]. This interaction is not complete until spatulous clustering occurs through the PLADs of the TNFR2 trimer, which then triggers a robust signaling pathway (Fig. 2B) [24]. It is important to mention that regardless of the presence of TNF-α (Mb-TNF-α or s-TNF-α), membrane-bound TNFR2 can still form dimeric or trimeric structures through their PLADs on the cell surface [22, 25]. However, this alone is not enough to fully activate the full signal. Understanding the complex structure of TNFR2 and its intricate interactions with TNF-α is critical for the development potent anti-TNFR2 antibodies or inhibitors. These could be utilized alone or as a part of an effective therapeutic regimen to combat various malignancies, including BC.

## TNFR2 signalosome in *cancer* in general and BC in specific

**Fig. 4:**
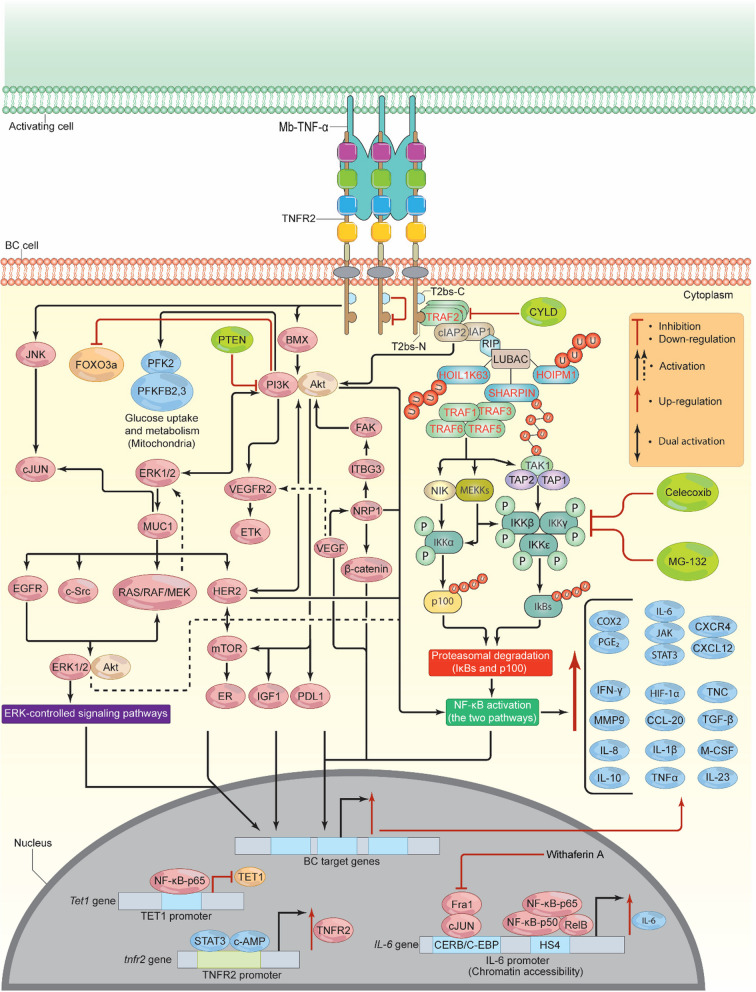
TNFR2/Mb-TNF-α signalosome pathway. Upon Mb-TNF-α binding to TNFR2, the T2bs-N region recruits TRAF2, which then recruits E3 ligases (cIAP1, cIAP2, RIP, and LUBAC). LUBAC attaches M1-linked ubiquitin chains, stabilizing the signaling complex and enhancing downstream signaling. This leads to the accumulation of TRAFs 1, 3, 5, and 6, which phosphorylate TRAF 3, releasing NIK. NIK stimulates MEKK1, 2, 3, and TAK1, which then phosphorylate, IKKα, IKKβ, IKKε, and KKγ, inducing NF-κB pathways. Inhibitors like Celecoxib, CYLD, and MG-132 can prevent NF-κB activation. In addition, cIAP1 and cIAP2 stimulate BMX and JNK/cJUN, which induce PI3K/Akt, resulting in the activation and phosphorylation of several pathways (ETK/VEGFR2, ERK1/2, IGF1, HER2, mTOR, NF-κB, FOXO3a, and PD-L1). HER2 and ERK1/2 induce positive feedback loop signals, which enhance BC cell metabolism and proliferation via NF-κB, c-MYC, STATs, SAP-1a, AP-1, Elk-1, Cyclin D1, and ER. TNFR2 also stimulates HIF-1α, which in turn activates VEGF and its associated signaling pathways, leading to NF-κB-p65 activation. TNFR2 in its promoter contains binding sites for both STAT3 and c-AMP, the binding of which induces TNFR2 overexpression. Epigenetically, TNFR2 enhances IL-6 overexpression and down-regulates TET1 expression. As a result of TNFR2 activation, multiple proteins will be stimulated (CCL-2, PDL-1, IL-1β, IL-6, IL-8, IL-10, IL-23, M-CSF, MMP-9, COX-2/PGE_2_, TGF-β, CXCR4/CXCL-12, TNF-α, TNC, IFN-γ, and HIF-1α), leading to drug resistance, altered metabolism, migration, invasion, and the development of EMT. TNFR2, tumor necrosis receptor type 2; Mb-TNF-α, membrane-bound tumor necrosis factor; TRAF, TNF receptor associated factor; T2bs-C, TRAF2-binding site C; T2bs-N, TRAF2-binding site N; cIAP, cellular inhibitor of apoptosis; SHARPIN, shank-associated RH domain-interacting protein; HOIL-1-K63-Ub, heme-oxidized IRP2 ubiquitin ligase 1; HOIP-M1-Ub, HOIL-1-interacting protein; LUBAC, linear ubiquitin chain assembly complex; NIK, activate NF-κB inducing kinase; MEKK, MAP kinase/ERK kinase; TAK1, transforming growth factor-activated kinase 1; TAP, TAK1-binding protein complex; PI3K, phosphoinositide 3-kinases; Akt, protein kinase B/serine-threonine kinase; CYLD, cylindromatosis; PFK-2, phosphofructokinase-2; PFKFB2, 3, 6-phosophofrcto-2-kinase/fructose-2, 6-biphosphatase 3; mTOR, mammalian (or mechanistic) target of rapamycin; EGFR, epidermal growth factor receptor; PTEN, phosphatase and tensin homolog; VEGF, vascular endothelial growth factor; ETK, endothelial/epithelial tyrosine kinase; BMX, marrow x-linked kinase; VEGFR2, vascular endothelial growth factor receptor 2; EMT, epithelial-mesenchymal transition; MMP-9, matrix metalloproteinase-9; FOXO3a, forkhead box O3a; JNK, c-jun N-terminal kinase; ERK, extracellular signal-regulated kinase; MUC-1 and MUC1-C, Mucin; c-MYC, cellular myelocytomatosis; STAT, signal transducer and activator of transcriptions; AP-1, activator protein-1; IL-6, interleukin 6; IL-8, interleukin 8; IL-10, interleukin 10; IL-23, interleukin 23, M-CSF, macrophage colony stimulating factor; TET, ten-eleven translocation; COX-2, cyclooxygenase-2; PGE_2_, prostaglandin E2; TGF-β, transforming-growth factor β; IFN-γ, interferon gamma; HIF-1α, hypoxia inducible factor 1 alpha; NRP-1, Neuropilin-1; TNC, Tenascin C; ITBG3, integrin 3; FAK, focal adhesion kinase pathway; IKKs, IκB kinases; IGF1, insulin-like growth factor 1; HER2, human epidermal growth factor receptor 2

Once TNF-α and TNFR2 have fully bound, a potent signalosome-complex cascade is triggered. Both forms of TNF-α have the ability to activate TNFR2. The soluble form, however, has a lower affinity for TNFR2 after being cleaved by TNF-α-converting enzyme (TACE), also known as a disintegrin or metalloproteinase domain-containing 17 (ADAM17) [26]. Therefore, TNFR2 has a tendency to bind more effectively and with greater affinity to the Mb-TNF-α, as mentioned previously [23]. Typically, six TNF receptor-associated factors (TRAFs 1–6) are found in mammalian cells, of which TRAFs 1, 2, 3, and 5 are responsible for initiating the signaling of TNFR2 (Fig. 4). While TRAF6 may engage in signaling through the toll/interleukin-1 receptors family, it may also act via other TNFR superfamily members [27]. Essentially, near the C-terminal region of TNFR2, there is a short sequence motif that interacts with 3 adapter-homotrimer TRAF2 protein promoters [28]. However, TRAF2-binding site C (T2bs-C) has the ability to inactivate TNFR2 signaling by competing with conventional TRAF2’s binding site N (T2bs-N), preventing it from interacting [27]. The TRAF2 adapter-homotrimer further accumulates and associates with two known ubiquitin–protein E3 ligases, the cellular inhibitor of apoptosis 1 and 2 (cIAP1 and cIAP2). The coiled-coil domain of TRAF2 also associates with three E3 ligases, including shank-associated RH domain-interacting protein (SHARPIN), heme-oxidized IRP2 ubiquitin ligase 1 (HOIL-1-K63-Ub) and HOIL-1-interacting protein (HOIP-M1-Ub), which together form the linear ubiquitin chain assembly complex (LUBAC)). Thus, TRAF2, together with TRAFs 1, 3, 5, and 6, activates the nuclear factor kappa-light-chain-enhancer of activated B cells (NF-κB) inducing kinase (NIK), which regulates activation of the noncanonical NF-κB pathway, and p100 pathway [29–36].

Specifically, TRAF 3 degradation results in the dissociation and release of the NIK, which then interacts with kinases such as MAP kinase/ERK kinase (MEKK1,2, and 3), a typical protein kinase C, and transforming growth factor-activated kinase 1 (TAK1). Specifically, TAK1 will associate with the TAK1-binding protein complex (TAP1 and 2) and undergo auto-phosphorylation, after which it mediates the activation of TAP1, which then together phosphorylates the IκB kinases (IKKs) complex (IKKα, IKKβ, IKKε, and IKKγ; also known NEMO) (Fig. 4) [29–36]. Indeed, these processes are critical for the activation of NF-κB’s canonical and non-canonical pathways [27, 30, 35, 36]. Therefore, the two pathways appear to control different but overlapping genes, depending on the cancer type. For instance, stimulation of the constitutively active phosphoinositide 3-kinases (PI3K)-protein kinase B*/*serine-threonine kinase (PI3K/Akt) pathway by TNFR2 phosphorylates IKKβ (pIKKβ), which in turn activates the NF-κB canonical pathway. In contrast, Mb-TNF-α-TNFR2 interaction activates both pathways [37], which are very important factors in the progression and development of BC [38]. However, other TNFR family members (CD40, EDAR, and XEDAR) may be able to inhibit TNFR2-mediated NF-κB activation through a process involving ubiquitin-deubiquitinase cylindromatosis (CYLD). CYLD inhibits TRAFs, particularly TRAFs 2 and 6 [39], and can be inactivated via phosphorylation by NEMO [40]. At the transcriptional level, TNFR2 comprises responsive regions and a consensus site for c-AMP binding, which promote TNFR2 overexpression (Fig. 4) [41].

Conversely, TNFR2-activated PI3K/Akt and phosphoinositide 3-kinases (PI3K)-protein kinase B*/*serine-threonine kinase (PKB/Akt) can mediate BC cell survival by inducing cellular growth factors, such as insulin-like growth factor 1 (IGF-1), promoting drug resistance, and preventing of DNA damage in cancerous cells [42–46]. The activated PI3K/Akt can thus phosphorylate various proteins such as phosphofructokinase-2 (PFK-2) and 6-phosophofrcto-2-kinase/fructose-2, 6-biphosphatase 3 (PFKFB2, 3), which play significant roles in BC glucose metabolism [47]. In addition, TNFR2-mediated activation of NF-κB can also occur via PI3K/Akt/mammalian (or mechanistic) target of rapamycin (mTOR) signaling pathway in BC. This activation occurs independently of HER2, HER3, and epidermal growth factor receptor (EGFR), and is coupled with the loss of the phosphatase and tensin homolog (*PTEN*) tumor suppressor gene, one of the most mutated genes in BC (Fig. 3) [48–50]. Furthermore, this TNFR2-dependant pathway can induce the overexpression of the *HER2* gene [51]. HER2 may also stimulate the PI3K/mTOR signaling pathway, causing ER phosphorylation and HER2 overexpression [52]. As a result, mTOR stimulates and phosphorylates various transcription factors and signaling pathways that actively contribute to the development of BC [53].

Interestingly, PTEN loss, on the other hand, may have a detrimental effect on the immune system by decreasing CD8^+^ T cells infiltration mediated by vascular endothelial growth factor (VEGF) or chemokine (C–C) motif ligand 2 (CCL-2), and increasing programmed death ligand 1 (PDL-1) overexpression mediated by PI3K [25]. PTEN loss additionally triggers the secretion of NF-κB-mediated IL-1β, C-X-C motif chemokine ligand 1 (CXCL-1), IL-6, CCL-20, IL-23, and macrophage colony stimulating factor (M-CSF). This leads to an accumulation of MDSCs, T-regs, and myeloid cells in the BC TME (Fig. 4) [25], thereby amplifying the impact of TNFR2 activation. Additionally, activated PI3K/Akt may interact with endothelial/epithelial tyrosine kinase (ETK)/marrow x-linked kinase (BMX) and VEGF receptor 2 (VEGFR2), resulting in TNFR2/PI3K/Akt/ETK/VEGFR2 signaling complex, leading to cell proliferation and development [54, 55]. Likewise, independently of TRAF2, BMX promotes Akt synthesis and regulates TNFR2-induced NF-κB activation by binding to a specific domain at the C-terminus of TNFR2 [55]. Conversely, NF-κB/PI3K/Akt inhibition suppresses migration, invasion, epithelial-mesenchymal transition (EMT), and matrix metalloproteinase-9 (MMP-9) in BC [56].

An important regulator of cell cycle arrest and DNA damage-induced apoptosis is forkhead box O3a (FOXO3a). It has been shown that PI3K/Akt activation promotes the phosphorylation of FOXO3a, which in turn inhibits its activity (Fig. 3) [57]. In fact, TNFR2 signaling can induce the c-jun N-terminal kinase (JNK) phosphorylation in TRAF2-independent manner [58, 59], supporting BC migration and invasion [60]. A TNFR2 deletion mutant without its cytoplasmic domain was unable to activate JNK through TNFR2 [59]. However, TRAF1 and TRAF3 may interfere with TNFR2-induced NF-κB and JNK activation by acting in a manner opposite to that of TRAF2 [58]. Interestingly, via TNFR2-induced NF-κB activation, the PI3K/Akt/extracellular signal-regulated kinase (ERK1/2—mitogen-activated protein kinase, MAPK) signaling pathway can induce the oncoprotein Mucin (MUC-1 and MUC1-C). This in turn increases TNF-α expression and interacts with EGFR, c-Src, c-jun, RAS/RAF/MEK, HER2, and other tyrosine kinase receptors [61, 62]. MUC-1 blockage successfully inhibited BC development [61]. Indeed, the induced EGFR stimulates and phosphorylates the RAS/RAF/MEK/ERK and ERK/Akt/NF-κB signaling pathways, the most essential signaling chains among the MAPK cell signaling networks, which are significantly expressed in BC [63–66]. Activated ERK subsequently activates and phosphorylates a variety of ERK-controlled signaling pathways, including NF-κB, cellular myelocytomatosis (c-MYC), signal transducer and activator of transcriptions (STATs), SAP-1a, activator protein-1 (AP-1), Elk-1, Cyclin D1, and the ER [66–69]. Notably, the activated TNFR2-NF-κB not only directly induces Cyclin D1 [38], but it also enhances the production of TRAF1 and TRAF2, which functions as a positive feedback loop for NF-κB activation in BC [38].

Moreover, c-MYC can also be induced through the TNFR2-activated ER and HER2 resulting in the MAPK/HER2/ER/c-MYC signaling pathway [70], and can therefore positively modulate glucose and glutamine uptake in favor of BC [71]. The constitutive activation of NF-κB and AP-1 by TNFR2 can lead to epigenetic dysregulation in metastatic BC. Constitutively activated NF-κB and AP-1 can enhance chromatin accessibility and gene transcription in interleukin 6 (IL-6) promoters, leading to increased IL-6 production (Fig. 4) [72]. Following phosphorylation and activation, AP-1 molecules (i.e., Fra-1 and c-Jun) and NF-κB molecules (i.e., p50, p65, and RelB) exhibit specific binding to the CERB/C-EBP enhancer and the NF-κB binding motif (HS4), respectively (Fig. 4) [72]. Ndlovu and colleagues showed that withaferin A, a natural compound, effectively decreased the levels of Fra-1 protein, reduced phosphorylation of NF-κB-p65, and decreased the levels of RelB protein, resulting in the inhibition of constitutive production of IL-6 [72]. The findings suggest that, in addition to AP-1, both NF-κB signaling pathways play a role in the epigenetic changes and increased production of IL-6 in highly metastatic BC. Likewise, the dioxygenases, specifically the ten-eleven translocation (TET1, TET2, and TET3) enzymes, are of particular interest in cancer. These enzymes are frequently found to be down-regulated in various types of tumors, including BC [2]. They have a crucial role in facilitating DNA demethylation and regulating gene transcription through epigenetic mechanisms.

The enzyme TET1 is responsible for the conversion of 5-methylcytosine (5mC) to 5-hydroxymethycytosine (5hmC) and 5-formylcytosine (5fC), and subsequently to 5-carboxylcytosine (5caC) [2]. Remarkably, NF-κB-p65 significantly decreased the expression of the *Tet1* gene in TNBC. This reduction occurred as a result of NF-κB-p65 binding to the consensus promoter region of the *Tet1* gene following treatment with TNF-α and lipopolysaccharide (LPS). The repression of TET1 induced by TNF-α was significantly reduced when NF-κB was blocked using MG-132, a known inhibitor of NF-κB (Fig. 4) [2]. This observation suggests that the TNFR2 promoter and several BC genes might be hypermethylated as a result of the downregulation of TET1. These results showed that treatment with both TNF-α and LPS did not have a significant impact on the activity of TET2 and TET3 [2]. However, it did lead to the repression of TET1, suggesting that the canonical TNF-α/TNFR2/NF-κB-p65 signaling pathway selectively affects TET1. The observation that TET1 repression was specific to TNBC and not the luminal or HRE2 BC subtypes highlights the necessity for further investigation into the NF-κB-p65-mediated TET1 repression (Fig. 4). It is also important to analyze whether the non-canonical NF-κB pathway plays a positive role in the repression of TET1. Supporting inflammatory BC (IBC) signaling cascade, following the induction of constitutive NF-κB via TNFR2, a series of pro- and anti-inflammatory pathways can be stimulated including, JAK/STAT, and cyclooxygenase-2 (COX-2) [73]. The intimate relationship between NF-κB and COX-2 has been investigated in BC, since NF-κB constitutive stimulation was found to increase the expression of COX-2. Celecoxib, a selective inhibitor of COX-2, effectively inhibited the nuclear translocation and DNA binding activity of NF-κB-p65 [74]. This resulted in the suppression of NF-κB-p65’s transcriptional stimulation by inhibiting the downstream signals of IKKs and IκB proteins’ breakdown [74]. Remarkably, the addition of phorbol-12-myristate-13-acetate (PMA) increased the motility and invasion of BC cells by phosphorylating IKKα, IKKβ, IκBα, and Akt. This phosphorylation facilitated the transport of NF-κB-p65 into the cell nucleus, which then activated *COX-2* and led to an increase in the secretion of prostaglandin E_2_ (PGE_2_) (Fig. 3) [75]. Transfection of N-myc downstream-regulated gene 2 (NDRG-2) in these cells, which is a tumor suppressor gene with low expression in BC, counteracted the aforementioned COX-2-mediated PGE_2_ activation. However, Akt protein remained unaffected, suggesting that inhibition of NF-κB-p65 and Akt would consequently result in a down-regulation of COX-2 [70]. Together these pathways, in addition to activated NF-κB, produce various pro- and anti-inflammatory mediators including, C-reactive protein (CRP), transforming-growth factor β (TGF-β), interferon-gamma (IFN-γ), TNF-α, and various chemokines (e.g. CXCR4/CXCL-12) and cytokines (e.g. interleukins (IL-6, IL-8, IL-10, IL-1β) (Fig. 4) [73]. In fact, the transcriptional complex of NF-κB/IL-6/JAK/STAT may boost ER-α synthesis. Increased levels of IL-6 promote HER2 expression through the activation of STAT3, indicating that the TNFR2/NF-κB/IL-6/JAK/STAT3/HER2 signaling pathway may play a significant role in the progression and the establishment of EMT, producing a highly aggressive form of BC [73]. HER2 is capable of inducing NF-κB and Akt expression, thus maximizing the effect of TNFR2 (Fig. 4) [76]. In fact, TNF-α and IL-6 could effectively induce STAT3-mediated TNFR2 overexpression. STAT3 selectively binds to the − 1,578 region of the TNFR2 promoter, thus inducing its up-regulation (Fig. 4) [77]. Mechanistically, TNFR2 may stimulate hypoxia-inducible factor 1 alpha (HIF-1α) expression through NF-κB. Multiple hypoxic-solid tumors, including BC, have established roles for HIF-1α [78]. Indeed, HIF-1α can induce a series of oncogenes and transcription factors that support the growth and development of BC [78]. Conversely, it was shown that Neuropilin-1 (NRP-1) and Tenascin C (TNC) interact in a new signaling pathway in BC leading to cell migration and invasion [79]. In this signaling cascade, NRP-1 activates integrin 3 (ITBG3), which interacts with and phosphorylates focal adhesion kinase (FAK) at Tyr397. Together, they phosphorylate Akt at Ser473 which in turn promote NF-κB-p65, resulting in the NRP-1/ITBG3/FAK/NF-κB-p65 cascade and TNC activation. Also, NRP-1 increases the overexpression of TNFR2 and reduces the expression of TNFR1, PI3K/Akt-308, and the anti-apoptotic BC resistant protein (BCRP/ABCG2) (Fig. 4) [79]. Importantly, signals from VEGF, a key angiogenic molecule, significantly boosted NRP-1 production and activity [80]. VEGF/NRP-1 phosphorylate NF-κB-p65 and β-catenin, which in turn leads to the VEGF/NRP-1/NF-κB-p65 or VEGF/NRP-1/β-catenin signaling cascades, accelerating BC cell motility, invasion, metastasis, and EMT initiation [80]. Indeed, NF-κB-p65 plays a significant role in the above-mentioned process, suggesting a potential positive feedback loop between TNFR2 and NRP-1 (Fig. 4). Taken together, TNFR2 signaling pathway seems to have a systemic impact that significantly contributes to the biology of BC. This suggests that its effective suppression may provide novel treatment possibilities due to its definite role in promoting BC development.

## Signaling via TNFR2 inhibits BC cell death by apoptosis

In order for cancer to grow and survive, reducing or inhibiting the burden of apoptosis and apoptotic signals provides an effective strategy. TNFR2 can inhibit apoptosis directly or indirectly through recruiting alternative signaling pathways; however, this role is still controversial. Activated NF-κB has the ability to increase the expression of cellular FLICE (FADD-like IL-1β-converting enzyme)-inhibitory protein (c-FLIP) [81], which is structurally similar to caspases 8 and 10. C-FLIP aggressively competes with pro-caspase 8, likely blocking the autocatalytic degradation of caspase 8 and suppressing the activation of both caspases through associating with the fas-associated protein-death domain (FADD), TRAF2, TRADD, and receptor-interacting protein kinase 1 (RIPK1) (Fig. 4) [41]. In contrast to this mechanism, TNFR2 is able to cause apoptosis through its association with FADD, although the truncated version of FADD (FADD-DN) successfully abolished this action [82]. Supporting this notion, TNFR2-mediated TRAF2 depletion resulted in cell death via NF-κB-mediated CD95 activation [32].

**Fig. 5:**
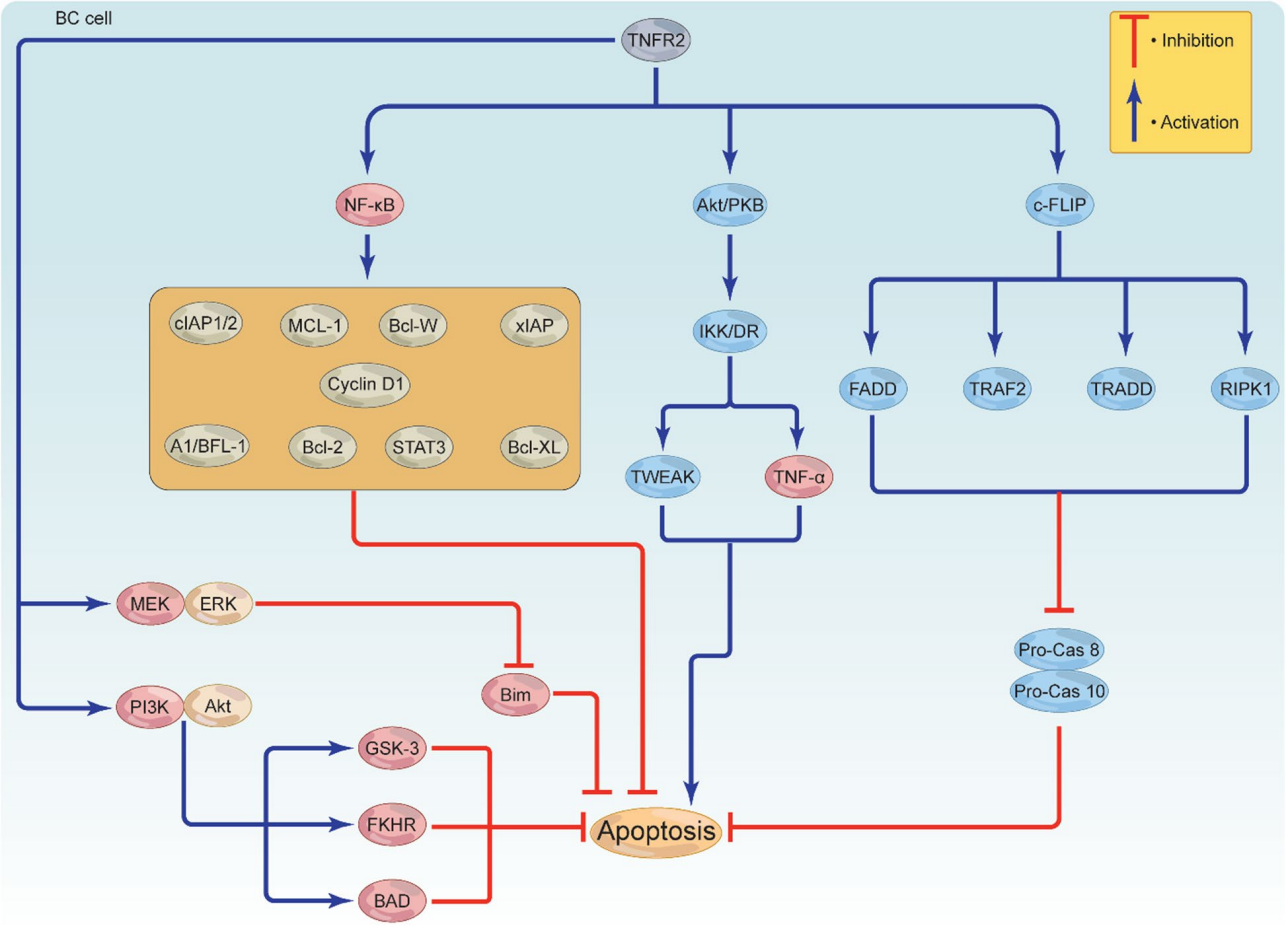
TNFR2-mediated apoptosis resistance in BC cells. TNFR2 triggers the activation of various proteins to help BC escape apoptosis. TNFR2-mediated Akt, NF-κB, and c-FLIP activation stimulate c-FLIP to activate FADD, RIPK1, TRAF2, and TRADD, inhibiting pro-Cas 8 and pro-Cas 10. While Akt activation leads to apoptosis via IKK/DR activation, inducing TNF-α and TWEAK, NF-κB activation induces a series of anti-apoptotic proteins, including Bcl-2, Bcl-XL, Bcl-W, xIAP, cIAP1/2, A1/BFL-1, Cyclin D1, STAT3, and MCL-1 to inhibit apoptosis. TNFR2 may also activate MEK/ERK and PI3K/Akt. MEK/ERK deactivates the protein Bim, while PI3K/Akt deactivates GSK-3, BAD, and FKHR. Together, these proteins inhibit apoptosis, leading to BC cell survival and growth. TNFR2, tumor necrosis receptor type 2; TNF-α, tumor necrosis factor; TRAF, TNF receptor associated factor; c-FLIP, cellular FLICE (FADD-like IL-1β-converting enzyme)-inhibitory protein; FADD, fas-associated protein-death domain; RIPK1, receptor-interacting protein kinase 1; TRADD, TNFR1-associated death domain; TWEAK, tumor necrosis-like weak inducer of apoptosis; BAD, Bcl-2-associated agonist of cell death; pro-cas8, pro-caspase 8; pro-cas10, pro-caspase 10; Bcl-2, B cell lymphoma 2; Bcl-XL, B cell lymphoma extra-large, Bcl-W, B cell lymphoma W; xIAP, X-linked inhibitor of apoptosis protein; cIAP, cellular inhibitor of apoptosis; STAT3, signal transducer and activator of transcription 3; MCL-1, Myeloid cell leukemia 1; GSK-3, glycogen kinase synthase-3; FKHR, fork-head in rhabdomyosarcoma; PI3K, phosphoinositide 3-kinases; Akt, protein kinase B/serine-threonine kinase; ERK, extracellular signal-regulated kinase

It has been postulated that interaction of activated-Akt/PKB with IKK/death receptor (DR) through the transcriptional control of two genes, TNF-α and tumor necrosis-like weak inducer of apoptosis (TWEAK), may trigger apoptosis possibly via a yet to be defined mechanism (Fig. 5) [83]. Moreover, through the activation and control of multiple anti-apoptotic genes including B cell lymphoma 2 (Bcl-2), B cell lymphoma extra-large (Bcl-XL), B cell lymphoma W (Bcl-W), X-linked inhibitor of apoptosis protein (xIAP), cIAP1/2, A1/BFL-1, Cyclin D1, STAT3 and Myeloid cell leukemia 1 (MCL-1), activated NF-κB negatively regulates apoptosis, leading to cell survival and growth (Fig. 5) [84–87]. Interestingly, activated MEK/ERK can target and phosphorylate the pro-apoptotic protein Bim, leading to apoptosis inactivation [88]. TNFR2 has the ability to prevent BC death by activating the PI3K/Akt signaling pathway. This pathway then inactivates glycogen kinase synthase-3 (GSK-3), Bcl-2-associated agonist of cell death (BAD), and fork-head in rhabdomyosarcoma (FKHR), leading to apoptosis-resistant BC cell (Fig. 5) [89]. Altogether, these data suggest that signaling from TNFR2 may trigger various direct or indirect anti-apoptotic pathways that can interact with each other to generate a highly complex apoptotic suppressive environment favoring BC growth and development. Consequently, targeted inhibition of TNFR2 could result in reduced expression of survival genes and facilitate apoptosis in both the immunosuppressive cells as well as BC cells.

## Signals from TNF-α/TNFR2 mediate membrane permeabilization to enhance BC invasion and metastasis

**Fig. 6:**
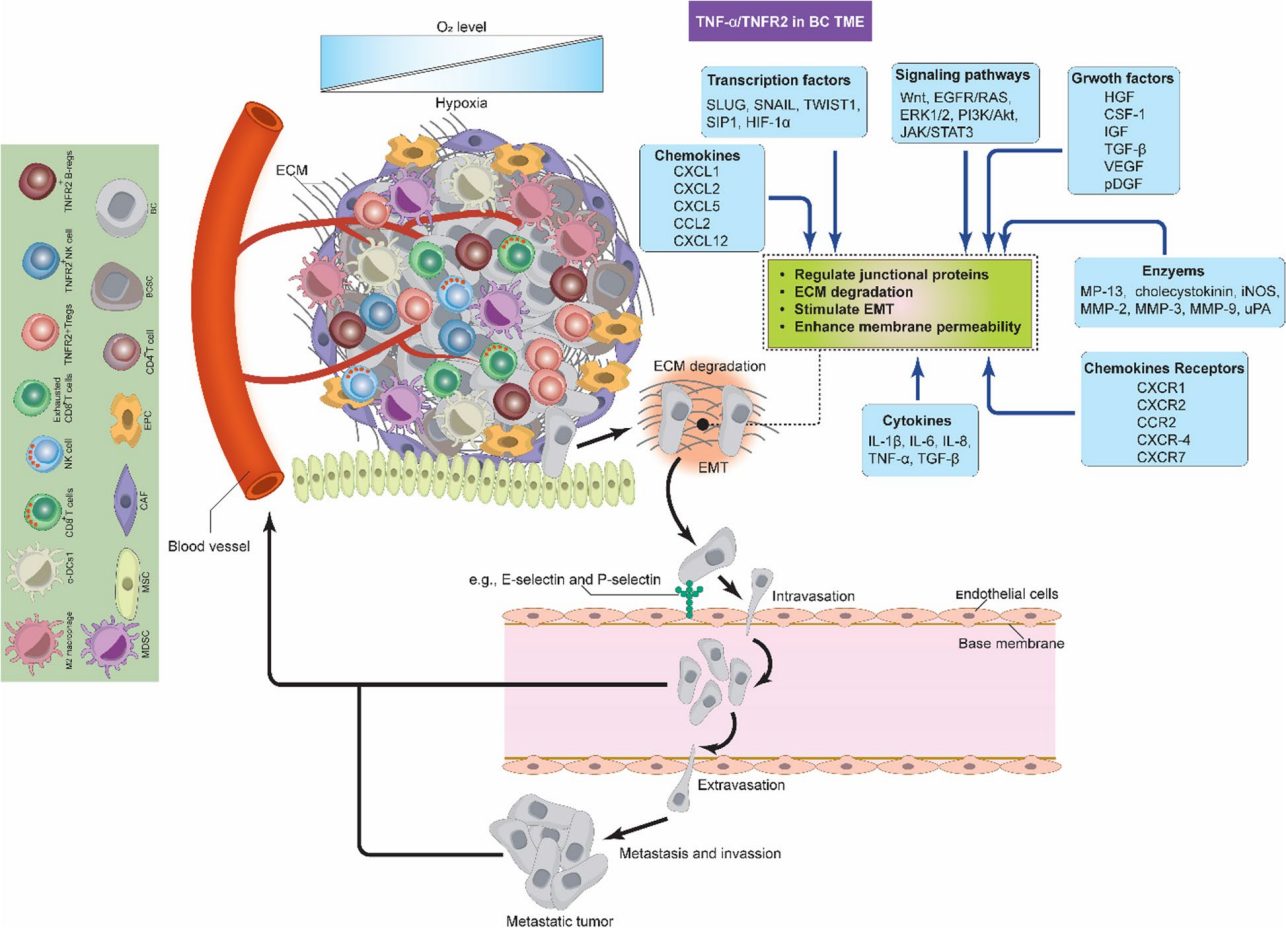
TNF-α/TNFR2-mediated invasion and metastasis in BC TME. TNFR2 signals can stimulate various proteins and signaling pathways, supporting almost all aspects of BC cell invasion and metastasis. For tumor cells to move from the primary tumor site by undergoing intravasation first and then extravasation, several enzymes, such as MMPs, must degrade the ECM. Many signaling pathways, in addition to several cytokines, chemokines, chemokine receptors, and growth factors, control the endothelial junctional proteins to induce membrane permeability. These events facilitate the movement of tumor cells, proliferation, vascularization, migration, and metastasis of BC to generate new tumors at distant sites and organs. c-DC1, type 1 conventional DCs; BC, breast cancer; BCSCs, breast cancer stem cells; MDSCs, myeloid-derived suppressor cells; T-regs, T regulatory cells; B-regs, B regulatory cells, CAFs, cancer associated fibroblast; EPCs, endothelial; MSCs, mesenchymal stem cells; TME, tumor microenvironment; ECM, extracellular matrix; MMPs, matrix metalloproteinases; iNOS, inducible nitric oxide synthase; NO, nitric oxide; TNF-α, membrane-bound tumor necrosis factor; TNFR2, tumor necrosis factor receptor type two; IL-1β, interleukin 1 beta; IL-6, interleukin 6; IL-8, interleukin 8; HIF-1α, hypoxia inducible factor 1 alpha; VEGF, vascular endothelial growth factor; pDGF, platelet-derived growth factor; IGF, insulin-like growth factor; FGF, fibroblast growth factor; HGF; CSF-1, colony stimulating factor 1; TGF-β, transforming-growth factor β; uPA, urokinase-type plasminogen; TWIST1, twist related protein-1; CCL-2, chemokine (C–C) motif ligand 2; CXCR1, C-X-C chemokine receptor 1; CXCR2, C-X-C chemokine receptor 2; CXCR-4, C-X-C chemokine receptor 4; CXCR7, C-X-C chemokine receptor 7; CCR2, C–C chemokine receptor type 2; CXCL-1, C-X-C motif chemokine ligand 1; CXCL-2, C-X-C motif chemokine ligand 2; CXCL-5, C-X-C motif chemokine ligand 5; CXCL-12, C-X-C motif chemokine ligand 12

Cellular motility is essential for BC invasion and plays a significant role in the metastatic process. During invasion and metastasis, cells detach from the primary tumor sites, reach the TME and the local parenchyma, invading (intravasating) nearby blood vessels and lymphatic tissues [90, 91]. From there on, the cells inter-circulate and undergo a dissemination process, then extravasate to form pre-metastatic niche before generating new colonies in distant organs, leading to several problems, including cancer recurrence (Fig. 6) [91]. Understanding the mechanisms behind invasion and metastasis is crucial to develop effective treatments, and recent reports have linked aberrant TNFR2 activation in response to these processes. As a matter of fact, TNFR2 signals can stimulate various proteins and signaling pathways to support almost all aspects of the invasion and metastasis. This was confirmed by multiple murine cancer models (colon, melanoma, and lung metastasis) that experimentally lack TNFR2 expression, which showed that the metastasis was dramatically inhibited in animals as compared to the control [92–94]. This suggests that the TNFR2 expression within TME favors cancer invasion and metastasis; however, the exact mechanisms are not yet fully understood.

One of the important events during invasion and metastasis is the EMT by which cancer cells undergo transformation from the epithelial to the mesenchymal phenotype to facilitate their motility. TNFR2 activates many inflammatory signaling pathways and membrane permeability-increasing mediators to support BC cells EMT and metastasis. NF-κB controls tight junction proteins by altering their distribution and reducing the expression of E-cadherin to enhance permeability as well as up-regulating EMT-associated proteins such as MP-13, MCP-1 (CCL2), cholecystokinin, MMP-2, MMP-3, MMP-9, and urokinase-type plasminogen (uPA) [91, 95–97]. Also, several EMT-associated transcription factors such as SLUG, SNAIL, twist related protein-1 (TWIST1), and SIP1 are induced by NF-κB [96]. These proteins and factors interact with each other to assist the tumor cells to protrude and degrade the ECM (Fig. 6) [98]. Moreover, VEGF also increases membrane permeability by altering the endothelial cells, increasing NO production by iNOS, increasing E-selectin expression, and activating several cascades such as ERK, PI3K/Akt, JNK, and Src, which together phosphorylate occludin and ZO-1. VEGF-mediated Src activation, in addition to TNF-α and platelet-derived growth factor (pDGF), phosphorylates vascular endothelial cadherin (VE-cadherin), β-catenin, and p120-catenin (Fig. 6) [99]. It should be noted that VEGF is a direct product of TNFR2-mediated HIF-1α activation. Collectively, these events disrupt the integrity of cell-to-cell contacts, priming BC cells for invasion and metastasis.

Various cytokines, chemokines and chemokine receptors play vital roles in the process of BC cell invasion and metastasis. For example, IL-6 promotes the EMT via JAK/STAT3-mediated ER-α regulation, as previously mentioned [100]. IL-8 enhances the permeability of the endothelium, facilitating the extravasation of tumor cells. Additionally, it signals through CXCR1 and CXCR2 to induce Src, which in turn phosphorylates VEGFR2 to reorganize junctional proteins [101]. IL-1β induces the phosphorylation of VE-cadherin [101] and increases the expression of P- and E-selectins [99]. TGF-β stimulates various signaling pathways, such as Wnt, EGFR/RAS, ERK1/2, and PI3K/Akt, and also modulates the function of platelet-derived growth factor receptor B (pDFGFR- β) to promote BC EMT, migration, metastasis, and differentiation [91]. CCL2 binding to CCR2 mediates proliferation, vascularization, migration, and metastasis of BC [101]. TNF-α-mediated CXCL1, CXCL2, and CXCL5 expression recruits neutrophils expressing CXCR2 to the TME of BC. The interaction of BC cells with neutrophils enhances the expression of several metastasis-associated genes in cancer cells, including TGF-β, IL-6, MMP-12, MMP-13, CXCR-4, and CXCR7 [102]. Signals from CXCR-4 interaction with its ligand CXCL-12 reduce the expression of VE-cadherin, occludin and ZO-1 [101]. Several cellular growth factors in addition to TGF-β, VEGF, and pDGF are also implicated. Hepatocyte growth factor (HGF), colony stimulating factor 1 (CSF-1), and insulin-like growth factor (IGF), promote angiogenesis and metastasis (Fig. 6) [103–106]. Following all these events, BC cells invade and metastasize various tissues, such as the lung, the brain and the bone marrow, to form the new tumor. Here, it should be noted that all the BC TME cells must contribute and interact with each other for the invasion and metastasis to be successful. The process of invasion and metastasis is very complex, and TNF-α/TNFR2 signaling is not the only decisive force; other factors might also be involved. This highlights the need for more research to determine the common signaling pathways or proteins to work as effective targets for immunotherapy or any possible combinatorial therapeutic strategies.

## Signals from TNFR2 activate breast *cancer* stem cells

**Fig. 7:**
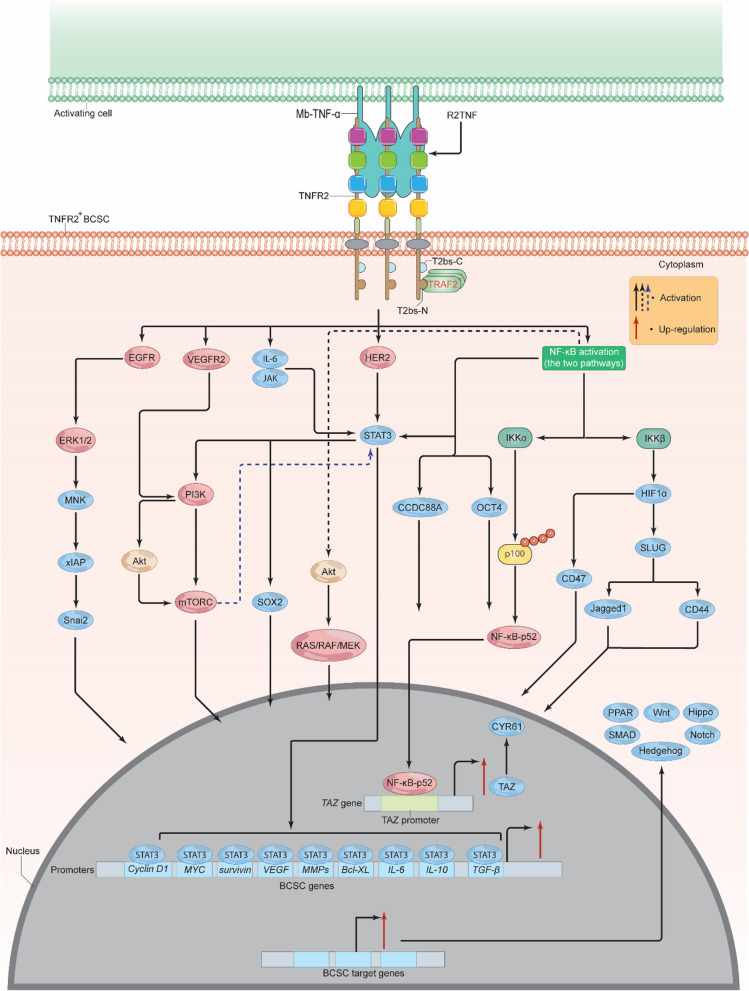
TNFR2 supports BCSC growth and development. Once TNFR2 is activated via Mb-TNF-α or R2TNF, multiple proteins including EGFR, VEGFR2, IL-6/JAK, HER2, and NF-κB are activated. EGFR activation leads to ERK1/2 phosphorylation, inducing MNK, xIAP, and Snai 2. VEGFR2 activation triggers PI3K/Akt activation, stimulating mTORC, which along with IL-6/JAK, HER2, and NF-κB, promotes STAT3 activation. STAT3 induces various oncogenic proteins, including Cycline D1, MYC, surviving, VEGF, MMPs, Bcl-XL, IL-6, IL-10, and TGF-β. IKKα and IKKβ activation promote HIF-1α, OCT4, CCDC88A, and Akt stimulation and the release of NF-κB-p52 to the nucleus and up-regulate TAZ-enhancing CYR61 production. HIF-1α will trigger CD47 and SLUG/Jagged1 or SLUG/CD44, while Akt stimulates the RAS/RAF/MEK/ERK pathway. These proteins lead to Notch, Hedgehog, Wnt, Hippo, SMAD, and PPAR stimulation, promoting BCSC self-renewal, EMT development, angiogenesis, and survival. TNFR2, tumor necrosis receptor type 2; Mb-TNF-α, membrane-bound tumor necrosis factor; TRAF, TNF receptor associated factor; T2bs-C, TRAF2-binding site C; T2bs-N, TRAF2-binding site N; Akt, protein kinase B/serine-threonine kinase; mTOR, mammalian (or mechanistic) target of rapamycin; EGFR, epidermal growth factor receptor; VEGF, vascular endothelial growth factor; VEGFR2, vascular endothelial growth factor receptor 2; EMT, epithelial-mesenchymal; MMPs, matrix metalloproteinases; c-MYC, cellular myelocytomatosis; STAT, signal transducer and activator of transcriptions; IL-6, interleukin 6; IL-10, interleukin 10; TGF-β, transforming-growth factor β; HIF-1α, hypoxia inducible factor 1 alpha; IKKs, IκB kinases; Bcl-2, B cell lymphoma 2; HER2, human epidermal growth factor receptor 2; PI3K, phosphoinositide 3-kinases; ERK, extracellular signal-regulated kinase; MNK, MAPK interacting kinase; PPAR, peroxisome proliferator-activated receptor

Breast cancer stem cells (BCSCs) are a small subset of BC cells with the potential to self-regenerate and spread to new sites in the body, as well as enhance the development, medication resistance, and recurrence of BC [107]. BCSCs are identified by the expression of certain cell-surface markers, including CD24, CD36, CD44, CD49f, CD55, CD90, CD133, CD326, E-cadherin, epithelial specific antigen (ESA), ATP-binding cassette transporter G-2 (A-BCG-2), and aldehyde dehydrogenase 1 (ALDH-A1) [107, 108]. Specifically, BCSCs-CD44^+^ can be distinguished by the expression of α2,3 sialylated core2 O-linked glycans [109]. Histologically, BCSCs are associated with positive HER2, expressing high levels of androgen receptor (AR) and moderate levels of PR, but very little to no ER [108]. A rising body of data indicates that TNF-α may play a crucial role in the establishment and maintenance of the BCSCs and that this impact is most likely due to its interaction with TNFR2 (Fig. 7) [110]. For example, through transcriptional upregulation of TAZ and its gene CYR61, which are pivotal for self-renewal and tumor initiation, TNF-α facilitated the expansion of both BCSCs-CD44^+^ and BCSCs-CD24^+^ [111]. Using siRNA to knock-down TAZ dramatically suppressed TNF-α-mediated mammosphere development, as well as the activation of BCSC-CD44^+^ and ALDH^+^ cells [111]. Knock-down of p100 decreased TAZ and CYR61 mRNA levels and prevented p100 processing to NF-κB-p52, demonstrating that TAZ controls the non-canonical NF-κB pathway. The expansion of BCSCs CD44^+^ and ALDH^+^ cells was inhibited by IKKα silencing. When TNF-α is present, it causes p100 to be processed into NF-κB-p52, which then attaches to the TAZ promoter and initiates transcription of the gene (Fig. 7) [111]. Supporting this notion, TNF-α induced the invasion and migration of BCSCs and increased expression of certain mesenchymal markers supporting their EMT. It also enhanced the intratumoral vessel formation capacity [112]. Moreover, SLUG—which is a critical EMT factor—activation by TNF-α or HIF-1α markedly increased the expression of CD44 and Jagged-1 as well as enhanced mammosphere initiation supporting BCSCs development. Knock-down of SLUG or both IKKα and IKKβ effectively diminished this process [113]. Conversely, p53 deficient BCSCs up-regulated HIF-1α, NF-κB, and SLUG leading to signaling through the NF-κB/HIF-1α/SLUG cascade. It should be mentioned here that TNF-α-mediated SLUG activation was supported by the canonical NF-κB-mediated activation of HIF-1αwhich enhanced the survival and tumorigenesis of BCSCs (Fig. 7) [113]. In fact, HIF-1α activation may also enhance the expression of integrin associated protein—CD47, which can boost the BCSC phenotype and mediate resistance to phagocytosis by macrophages [114]. However, other data suggest that TNF-α may negatively down-regulate BCSC genes [115]. Although these data strongly show that TNF-α/NF-κB interaction may assist in the establishment of the BCSC, it remains unclear whether TNFR2 is engaged directly or via signaling interactions with other molecules such as NF-κB. Recent research indicates that TNFR2 may promote the growth of cancer stem cells (CSCs) through a number of signaling pathways [110, 116]. The majority of these pathways also serve as effective TNFR2 downstream targets, promoting the expansion and development of BCSC. Moreover, in clear cell renal carcinoma (ccRCC) model of ccRCC expressing CD133 (ccRR-^CD133+^CSC), binding of TNF-α and/or R2TNF (a TNFR2 selective mutein agonist) to TNFR2 significantly phosphorylated VEGFR2, PI3K, Akt, and mTORC at amino acids Y1059, Thr308, Ser2448, and p110β respectively. This resulted in a TNFR2/VEGFR2/PI3K/Akt/mTORC signaling pathway that further induced the phosphorylation of STAT3 at serine 727 leading to a cascade of TNFR2/VEGFR2/PI3K/Akt/mTORC/STAT3, enhancing ccRR-^CD133+^CSC growth and survival [116]. Loss of TNFR2, STAT3, or any of the other activated kinases led to extensive apoptosis, mitochondrial damage, and generation of a wide variety of reactive species [116]. These data seem to support the notion that TNFR2-mediated NF-κB activation (conical and non-conical pathways) and other kinases may promote BCSC growth. Indeed, in addition to NF-κB, VEGFR2, PI3K, Akt, mTORC, and STAT3, BCSCs may employ JAK/STAT, TGF-β, and ER to promote their formation and proliferation, in addition to other signaling pathways such as Notch, Hedgehog, Wnt, Hippo, SMAD, and peroxisome proliferator-activated receptor (PPAR) (Fig. 7) [117], all of which may be influenced somehow by TNFR2 [117–119]. For instance, TNFR2-mediated activation of NF-κB or HER2 can trigger signals via STAT3 and Akt, which in turn activate the signaling cascades RAS/RAF/MEK/ERK, PI3K/Akt, and STATs, resulting in BCSC formation and survival as well as EMT initiation [107].STAT3 has a consensus response element in the promoter region of certain tumor genes. Thus, STAT3 can bind and activate targets such as cyclin D1, MYC, Bcl-XL, survivin, VEGF, IL-6, IL-10, MMPs, and TGF-β, all of which play important roles in the maintenance and growth of BCSCs (Fig. 7) [120, 121]. Paradoxically, some evidence suggests that STAT3 can also be induced through signals from the TNFR2/NF-κB-activated-PI3K/mTOR signaling pathway which can stimulate BCSC survival and growth [110]. STAT3, through the signaling pathway IL-6/JAK2/STAT3, was found to enhance the function, stability, and development of CD44^+^CD24^–^BCSCs [122].TNFR2-mediated activation of EGFR or HER2 can also induce ERK, which in turn mediates MAPK interacting kinase (MNK) activation. MNK subsequently targets and activates its downstream gene xIAP (potent anti-apoptotic protein). Activated xIAP increases the expression of the EMT mediator Snai2, which in turn can enhance BCSC stemness. xIAP suppression significantly reduced EGFR and Snai 2 activity, leading to MAPK inhibition, and mediated the transition between MAPK and NF-κB pathway activation (Fig. 7) [121]. NF-κB stimulation of OCT4 and CCDC88A resulted in the invasion and metastasis of BCSCs [121]. Furthermore, the phenotype of BCSCs might be established by a pathway involving the interactions of activated EGFR, STAT3, and sex-determining region Y-box 2 (SOX2) [123]. Likewise, TNFR2 may impact BCSC stemness, by enabling their interaction with different immunosuppressive cells like T-regs, MDSCs, and TAMs. This, in turn, promotes BCSC immune evasion, BC growth, and development. Various signaling pathways and proteins are produced and reciprocally activated by these cells, which are essentially or in part activated by TNFR2. Various signaling pathways, including NF-κB, Akt, IL-6/STAT3, NO/Notch, Wnt, and inhibitory molecules (prostaglandin-E2 (PEG_2_), indoleamine 2,3-dioxygenase-1 (IDO1)), in addition to multiple cytokines, chemokines, and their receptors, including IL-10, IL-13, TGF-β, macrophage colony-stimulating factor-1 (CSF-1), growth differentiation factor-15 (GDF15), C–C motif chemokine 1 (CCL1), CCL2, CCL5, CCR-5, C–C chemokine receptor type 4 (CCR-4), as well as receptor-type tyrosine-protein phosphatase ζ (PTPRZ1), and ephrin type-A receptor-4 (EPHA4), are interchangeably produced to mediate cross-talk between T-regs, MDSCs, TAMs, and BCSCs, activating each other and favoring BCSC self-renewal, drug resistance, metastasis, aggressiveness, and recurrence as well as BCSC immune escape [124]. All things considered, the notion supporting the interaction of TNFR2 with several signaling pathways and the complex interplay between T-regs, MDSCs, TAMs, TNFR2^+^BCSCs in BC TME is an open window for future research. Furthermore, understanding the full role of TNFR2 expression on BCSCs could facilitate the development of therapies for an effective elimination of these cells, thereby preventing BC progression.

## Signals from TNFR2 Activate HIF-1α

Because BC is a solid tumor, it forms a very hypoxic TME. This is due to the high BC cell proliferation inhibiting blood vessel function [125]. Remarkably, BC cells can survive this environment by over-expressing hypoxia-inducible factors (HIFs), which in turn upregulates a number of genes that enhance drug resistance, glucose uptake, cell proliferation, and metastasis, while reducing oxidative stress and apoptosis [125]. Of the different HIF proteins, HIF-1α overproduction in BC patients significantly promotes tumor metastasis and is associated with unfavorable clinical outcomes as well as mortality [126]. Although HIFs may be triggered in response to low oxygen levels, they can also be activated independently of the oxygen condition via a variety of signaling pathways such as STAT3, mTOR, MYC, Notch, and NF-κB [78, 127, 128]. Recent data suggest that NF-κB can interact with and regulate HIFs, particularly HIF-1α, under certain conditions and that signals delivered from TNFR2 may play a significant role in this process [78, 129]. In support of this, it was discovered that TNF-α increased the mRNA and protein levels of HIF-1α [129]. In fact, both the TNFR2-activated canonical and non-canonical pathways of NF-κB can stimulate HIF-1α [78]. Additionally, signals from HIF-1α may dramatically increase the expression of NF-κB, indicating a reciprocal activation of both transcription factors [128, 130]. More specifically, HIF-1α promotes the expression of both IKKβ and NF-κB-p65 by interacting with the hypoxia response element (HRE) in the *NF-κB* promoter sequence [131]. Consequently, the majority of tumorigenic genes that cause the overexpression of NF-κB might also function as HIF-1α target genes [132]. HIF-1α also induces the Wnt/β-catenin pathway by stimulating BCL9, while Wnt/β-catenin can then activate and enhance HIF-1α expression, in a positive feedback manner [133]. Wnt/β-catenin can also interact with TRAF2 that has been accumulated by TNFR2. TRAF2 then recruits TRAF 3, 5, and 6 to HIF-1α. Next, TRAF 6 (E3 ligase-catalyzed K63-ubiquitination) interacts with and activates HIF-1α in one of two ways.

**Fig. 8:**
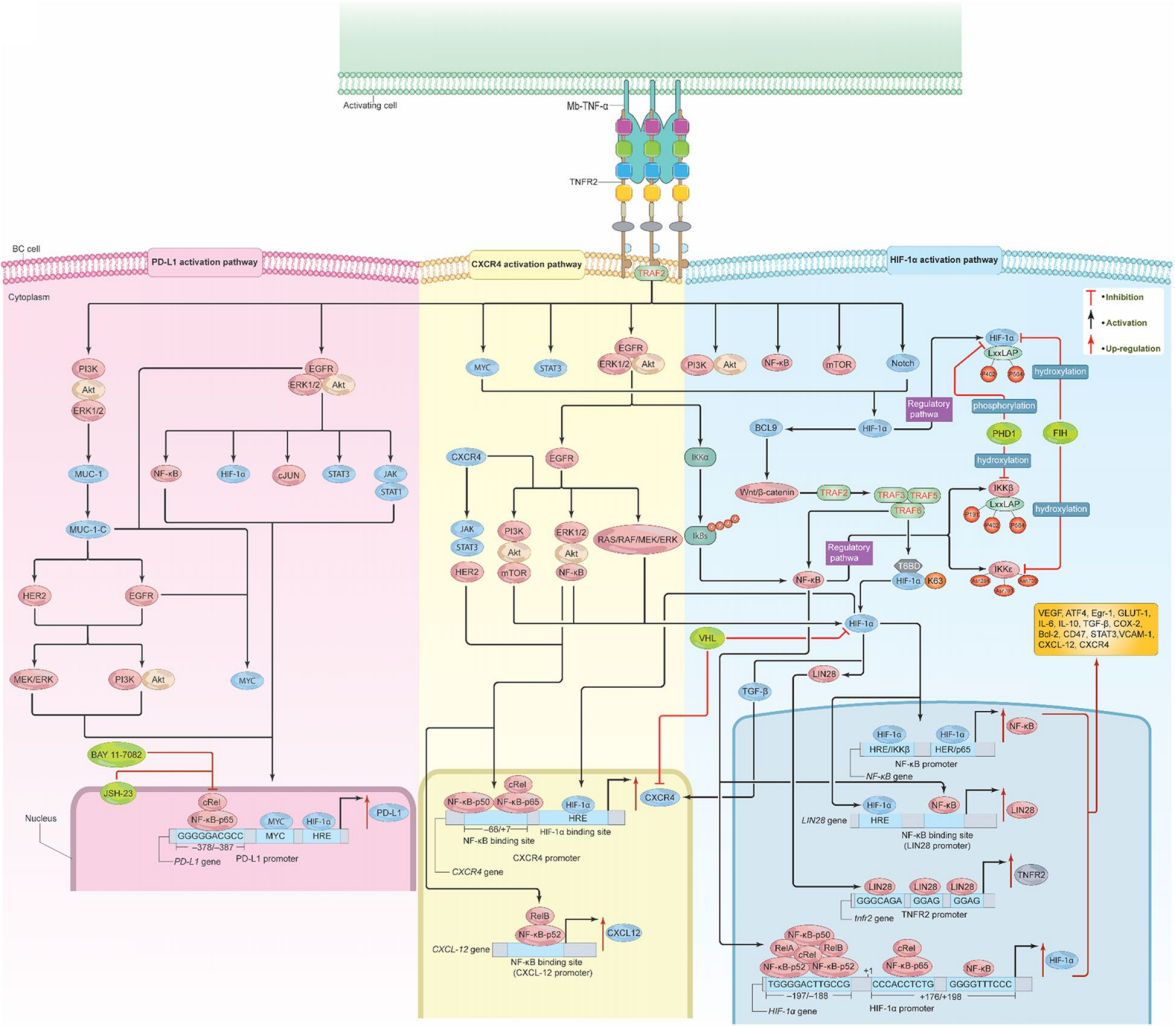
Signals from Mb-TNF-α/TNFR2 stimulate HIF-1α, CXCR4, and PD-L1. Binding of Mb-TNF-α to TNFR2 activates the HIF-1α, CXCR4, and PD-L1 pathways. In the HIF-1α pathway, TNFR2 signals activate STAT3, mTOR, MYC, Notch, NF-κB, EGFR/ERK/Akt, PI3K/Akt, and PI3K/Akt/mTOR. HIF-1α can also activate BCL9, stimulating Wnt/β-catenin, which interacts with TRAF2, leading to TRAF 3, 5, and 6 accumulations inducing NF-κB activation. NF-κB subunits bind to specific sequences in the HIF-1α promoter, enhancing its stimulation. TRAF 6 can directly mediate HIF-1α activation via ubiquitination of K63. EGFR/ERK/Akt-mediated IκBα phosphorylation can also activate HIF-1α, creating a positive feedback loop of several signaling pathways. LIN28, activated by HIF-1α, upregulates TNFR2 expression. IKKβ and HIF-1α possess shared residues targeted at PHD1. FIH inhibits IKKε, preventing its binding to TRAF3/TBK1 proteins. In the CXCR4 pathway, TNFR2 signals stimulate NF-κB, HER2, PI3K, Akt, mTOR, Wnt/βcatenin, the RAS/RAF/MEK/ERK pathway, and JAK/STAT. NF-κB subunits bind to CXCR4 and CXCL-12 promoters, inducing its overexpression. HIF-1α, NF-κB, TGF-β, and HER2 can also enhance CXCR4 expression. VHL, a negative regulator of HIF-1α, down-regulates CXCR4, but is inactive in most solid tumors, leading to CXCR4 overexpression. In the PD-L1 pathway, TNFR2 signals activate EGFR/ERK1/2/Akt and PI3K/Akt/ERK1/2, which in turn promote NF-κB, HIF-1α, cJUN, STAT3, and JAK/STAT3, up-regulating PD-L1. NF-κB-p65 binds to the PD-L1 promoter, enhancing its expression. The PI3K/Akt/ERK1/2 pathway also stimulates Mucin-1, activating HER2, EGFR, and MYC, which further up-regulate PD-L1. Inhibition of NF-κB-p65 via BAY 11–7082 and JSH-23 abolished PD-L1 overexpression. These pathways support BC growth, development, immune escape, drug resistance, angiogenesis, and metastasis. TNFR2, tumor necrosis receptor type 2; Mb-TNF-α, membrane-bound tumor necrosis factor; TRAF, TNF receptor associated factor; PI3K, phosphoinositide 3-kinases; Akt, protein kinase B/serine-threonine kinase; mTOR, mammalian (or mechanistic) target of rapamycin; EGFR, epidermal growth factor receptor; VEGF, vascular endothelial growth factor; ERK, extracellular signal-regulated kinase; MYC, cellular myelocytomatosis; STAT, signal transducer and activator of transcriptions; IL-6, interleukin 6; IL-10, interleukin 10; COX-2, cyclooxygenase-2; TGF-β, transforming-growth factor β; HIF-1α, hypoxia inducible factor 1 alpha; CXCR4, C-X-C chemokine receptor 4; HRE, hypoxia response element; VHL, Von Hppel Lindau; PD-L1, programmed cell death ligand 1; T6BD, TRAF 6 binding domain; PHD1, prolyl-hydroxylase 1; FIH, Factor-inhibiting HIF-1α; Bcl-2, B cell lymphoma 2; VCAM-1, vascular cell adhesion molecule-1

The first way is through NF-κB canonical pathway-dependent IKK activation [78, 134]. More precisely, the promoter region of HIF-1α comprises short sequences of ‘GGGGTTTCCC’ and ‘CCCACCTCTG’ from + 176 to + 198 downstream of the transcription start site. NF-κB and NF-κB/c-Rel can interact with these sequences to trigger transcription [135]. Alternatively, RelA/NF-κB-p52, RelB/NF-κB-p52, c-Rel, NF-κB-p65, and NF-κB-p50 can bind to the sequence TGGGGACTTGCCG, located –197/–188 bp upstream of the transcription start site of the HIF-1α promoter, via IKKα and IκBα. This interaction then induces transcription and translation of HIF-1α (Fig. 8) [136, 137].

The second way TRAF 6 activates HIF-1α is through binding of HIF-1α to TRAF 6 through its TRAF 6 binding domain (T6BD) (363PVESSD368). Following this interaction, TRAF6 mediates the ubiquitination of K63 of HIF-1α, activating it [138]. Subsequent, to its activation, HIF-1α can target, activate, and regulate various genes, including VEGF, activating transcription factor 4 (ATF4), early growth response-1 (Egr-1), glucose transporter-1 (GLUT-1), IL-6, IL-10, TGF-β, COX-2, Bcl-2, CD47, STAT3, and vascular cell adhesion molecule-1 (VCAM-1), as well as certain chemokines and their receptors, like CXCL-12 and its receptor CXCR4 (Fig. 8) [128, 130, 138–140].

Another mechanism by which HIF-1α can be activated is through TFNR2 stimulation of the EGFR/ERK/Akt pathway, which phosphorylates IκBα, hence activating both NF-κB and HIF-1α. PI3K/Akt activation can also induce HIF-1α stimulation [131]. Activated EGFR has the ability to stimulate the ERK/Akt/NF-κB, PI3K/Akt/mTOR, and RAS/RAF/MEK/ERK pathways, in addition to HIF-1α [65, 66, 141]. Interestingly, IKKβ and HIF-1α seem to share a similar regulatory fate, being targeted for degradation via prolyl-hydroxylase 1 (PHD1) (a natural inhibitor for HIF-1α under normal oxygen conditions) [142]. IKKβ and HIF-1α both possess the conservative LxxLAP domain which contains P402 and P564; these residues serve as the phosphorylation target site for PHD1. In addition, IKKβ includes another residue, P191, within the LxxLAP sequence. This residue serves as a target for PHD1 hydroxylation suggesting that the downregulation of PHD1 in the BC TME could significantly increase the activation of HIF-1α and the non-canonical NF-κB pathway. Moreover, IKKε contains three asparagine residues (Asn254, Asn700, and Asn701) that can be hydroxylated by factor-inhibiting HIF-1α (FIH, a natural inhibitor for HIF-1α under normal oxygen conditions), thus inhibiting IKKε activity and preventing its binding to TRAF3/TBK1 proteins (Fig. 8) [143]. Given the fact that both PHD1 and FIH are often defective in hypoxic tumors, there is likely a positive crosstalk between HIF-1α and TNFR2-mediated NF-κB in promoting BC development. However, this notion requires more investigation to elucidate the details of this crosstalk. Finally, both hypoxia and the introduction of TNF-α boosted the expression of endogenous TNF-α, TNFR2, LIN28 (an RNA-binding protein), and α-sarcomeric actin (α-SA) while reducing the expression of TNFR1. This suggests that hypoxia preferentially activates TNFR2 via interaction between HIF-1α and LIN28 [144].

The first intron of LIN28 has a highly conserved NF-κB DNA binding sequence. NF-κB can bind to this domain and vigorously activate LIN28 [145]. Interestingly, a comprehensive genome-wide analysis of thousands of LIN28 mRNA binding sites indicated that LIN28 can interact and control nearly 6,000 genes through the remarkably short motif ‘GGAGA(U)’ [146]. HIF-1α also can induce the expression of LIN28 but only at the mRNA level [147]. The mRNA 3’-UTR of the *TNFR2* promoter sequence has three conserved motifs: GGGCAGA, GGAG,’ and ‘GAT’ which can be recognized by LIN28. Binding of LIN28 to these motifs increases TNFR2 protein levels (Fig. 8) [148]. These mechanisms underscore the significance of TNFR2/HIF-1α in the regulation of various genes associated with BC angiogenesis, metastasis and cell survival. A comprehensive understanding of this pathway may facilitate research aimed at identifying potential therapeutic targets to modulate the aberrant TNFR2/HIF-1α activity, which is crucial for the treatment of cancers including BC.

## Signals from TNFR2 activate chemokine receptor CXCR-4

Chemokines are small molecules with molecular weights ranging from 8–12 kDa, engaging with certain G-protein–coupled receptors to induce a chemotaxis response, which results in cell motility, stimulation, and differentiation [140]. Among them, CXCL-12, also known as stromal cell-derived factor-1 (SDF-1), interacts and engages with its receptor, CXCR-4, to mediate a variety of cellular activities [140, 149]. Recent studies have also shown that CXCR-4 is implicated in the progression and development of various malignancies, as well as a poor clinical prognosis [150]. In BC, CXCR4 performs a crucial role in stimulating the growth, EMT, metastasis, and invasion of the disease [150]. CXCR4 over-expression is found in the majority of BCs relative to normal BC tissues [149]. Furthermore, CXCR-4 may influence *HER2* expression, which promotes cancer dissemination. However, HER2-negative BC with elevated CXCR-4 expression displayed aggressive behavior when compared to those with low CXCR-4 expression [149]. Furthermore, CXCR-4 overexpression may promote the development of BC, recurrence, and resistance to endocrine treatment through an estrogen-independent mechanism and is highly correlated with a poor prognosis and shorter patient survival rates, regardless of ER status [149]. In addition, practically all immunosuppressive cells in the BC TME exhibit high levels of CXCR-4 [151]. Despite the importance of CXCR-4 in the course of BC development, the exact signals mediated by this receptor remain largely elusive and seem to involve the interplay of numerous signaling pathways. Supporting this notion, TNFR2 signaling pathway appears to play a central role in this process. In fact, pretreatment of tumor cells with TNF-α increased the expression of CXCR4 at both mRNA and protein levels [152]. Furthermore, CXCR-4 up-regulation, through TNF-α, mediated tumor cell motility and the production of inflammatory cytokine and chemokine receptors/ligands, including CXCR4 [153]. Activation of CXCR4 resulted in the accelerated dissociation and release of membrane-bound TNF-α [154]. However, the particular impacts of the CXCR4 pathway and TNF-α/TNFR2 in BC have not been fully elucidated. TNFR2-mediated activation of NF-κB may also increase CXCR4 expression. Indeed, recent findings have shown that the *CXCR4* promoter region comprises sequences (–66 to + 7) with which NF-κB can interact. In this scenario, hepatocyte growth factor (HGF) treatment activates the NF-κB-p50 and NF-κB-p65 proteins. These two proteins then actively bind to the promoter of CXCR4 to facilitate its transcription (Fig. 8) [155]. Likewise, HIF-1α can also bind to a particular sequence inside the *CXCR4* promoter region (HRE) that may potently induce CXCR4 transcription. Similar to the two subunits of NF-κB. Von Hppel Lindau (VHL), which is active in normoxic settings, has been shown to negatively regulate HIF-1α as well as down-regulate CXCR4. As a consequence, when hypoxia occurs in solid tumors like BC, VHL is inactive, causing HIF-1α to induce persistent expression of CXCR4 (Fig. 8) [155]. One study suggested that HIF-1α interaction with TGF-β induced the expression of CXCR4 and VEGF in BC, leading to bone metastasis [156]. Moreover, there has been evidence that TNFR2 acts as a direct trigger for CXCR4 activation. In this context, TNF-α therapy increased CXCR4 mRNA levels through the activation of both TNFR2 and TNFR1 [152]. This suggests that both receptors are involved in CXCR4 expression. Indeed, TNFR2-mediated TRAF2 recruitment in TNFR2^+/+^ mice activated NF-κB-p52/Re1B, which in turn stimulated the expression of CXCL-12. Consequently, CXCL-12 stimulation leads to an increase in the expression of CXCR4. Mice with TNFR2^−/−^ however, show CXCL-12 down-regulation, which in turn negatively affected CXCR4 expression levels [157]. As previously stated, TNF-α/TNFR2 interaction activates a variety of proteins and signaling pathways, including NF-κB, HER2, PI3K, Akt, mTOR, Wnt/βcatenin, the RAS/RAF/MEK/ERK pathway, and JAK/STAT. HER2, in particular, can stimulate NF-κB, which in turn enhances HER2 expression. Both activated HER2 and NF-κB can significantly increase CXCR4 expression (Fig. 8) [158], suggesting reciprocal interaction and activation between the two proteins. This reciprocal activation might play a central role in the progression of BC, hinting at the possible therapeutic targets when designing drugs in the future. Upon CXCR4 activation, the receptor utilizes three subunits of the G protein heterodimer (α, β, and γ) by converting GTP to GDP; combined, they activate multiple signaling pathways including PI3K/Akt/mTOR, RAS/RAF/MEK/ERK, PI3K/Akt/NF-κB, and JAK/STAT [149]. Collectively, these pathways play crucial roles in BC development, either independently or via TNFR2-induced CXCR4 activation.

## Signals from TNFR2 activate PD-L1

In the TME, the overexpression and secretion of certain immune checkpoint proteins (ICPs) such as programmed cell death 1 (PD-1) and its ligand, programmed cell death ligand 1 (PD-L1), as well as cytotoxic T lymphocyte antigen 4 (CTLA-4) have led to the establishment of a highly immunosuppressive environment that allows cancer cells to resist and evade immune annihilation. In response, different immune checkpoint inhibitor (ICI) treatments have been designed to target these proteins in certain cancers including BC [159]. Among them, the PD-1/PD-L1 pathway is central to BC pathophysiology and the development of resistance to therapy [160–162]. More specifically, in addition to BC cells, PD-L1 expression was found in Tumor infiltrating lymphocytes (TILs) (T cells, B cells, natural killer cells (NK cells)), dendritic cells (DCs), and macrophages [163–165]. In contrast, TNBC patients with TILs-expressing TNFR2 exhibited a more favorable disease response in comparison to those with TILs-expressing PD-1 [166]. Surprisingly, it has been shown that PD-L1 expression causes worse disease outcomes in TNBC by blocking the protective effects of both sTNFR1 and sTNFR2 [167]. Undeniably, various cytokines (TNF-α, TGF-β, IL-1α, IL-2, IL-4, IL-6, IL-7, IL-10, IL-12, IL-15, IL-17, IL-21, IL-27, and IL-32) and complement fragment C5a can induce *PD-L1* expression via different mechanisms involving STAT3 and NF-κB-p65 transcription factors activation. However, IFN-γ produced by cancer-reactive T cells via STAT1 activation is the primary mechanism that increases *PD-L1* overexpression in TME [131, 167–169]. Furthermore, *PD-L1* gene expression is regulated by the interaction of various signaling pathways such as RAS/MAPK, PTEN/PI3k/Akt, and JAK/STAT, all of which activate a series of downstream transcription factors (IFR1, IFR3, HIF-1α, MYC, BRD4, Jun, and NF-κB) that bind to specific DNA sequences in the *PD-L1* gene promoter region and mediate its expression [131], while IFR3 does not have. Certainly, as we previously discussed, the majority of these signaling pathways triggered by the interaction of TNF-α and TNFR2, can also induce *PD-L1* overexpression in BC. In the promoter of the *PD-L1* gene, for instance, there is a sequence (GGGGGACGCC) located from –378 to –387 upstream of the transcription start site, after TNFR2-mediated NF-κB activation, NF-κB-p65 effectively binds and increases *PD-L1* gene transcription (Fig. 5) [131]. Moreover, IFN-γ not only increases PD-L1 production but also up-regulates the levels of TNFR2 [170], providing yet another mechanism by which PD-L1 is up-regulated. Likewise, HIF-1α has a HRE binding site in the *PD-L1* promoter (Fig. 8). TNFR2-mediated activation of EGFR/ERK/Akt signaling pathway can stimulate HIF-1α and NF-κB, which will then bind to HRE and NF-κB binding sites in the *PD-L1* promoter region, respectively, and thus, enhancing *PD-L1* expression [131]. Conversely, PI3K/Akt activation can also induce *PD-L1* overexpression at both the transcriptional and post-translational levels [171]. Not surprisingly, EGFR/ERK(MAPK) activation can also induce *PD-L1* up-regulation through the stimulation of both c-Jun and STAT3 as well as via JAK/STAT1 signaling pathway (Fig. 8) [171]. In addition to this, EGFR can trigger the expression MYC which is also capable of binding to a region of the PD-L1 promoter, increasing PD-L1 transcription [172]. The PI3K/Akt/ERK1/2 pathway can also upregulate PD-L1 by inducing the expression of MUC1 (Fig. 8) [61]. The PI3K/Akt/ERK1/2 pathway can also upregulate PD-L1 by inducing the expression of MUC1. MUC1 can activate a variety of proteins through its MUC1-C domain, including EGFR and HER2, which they will lead to induction of PI3K/Akt and MEK/ERK signaling pathways. MUC1-C also actively accumulates MYC and NF-κB-p65 on the *PD-L1* promoter, significantly raising *PD-L1* transcription rate in BC (Fig. 8) [173]. An additional NF-κB signaling pathway component may contribute to *PD-L1* expression. Treatment with TNF-α and TGF-β was enough to stimulate PD-L1 expression in BC cells. TGF-β enhanced PD-L1 promoter demethylation by decreasing the activity of DNA methyl transferases (DNMTs). While TNF-α stimulates the release of NF-κB-p65 via IKKε activation to be recruited to the demethylated promoter, which leads to PD-L1 overexpression. Blocking of IKKε with its known inhibitors IKK3, Bx795, and amlexanox reduced PD-L1 production [174]. Indeed, there is a dearth of evidence linking the direct interaction between TNF-α and TNFR2 with *PD-L1* expression in BC. TNF-α-mediated TNFR2 activation through NF-κB-p65 up-regulated *PD-L1* expression transcriptionally in a pancreatic cancer model [175]. NF-κB-p65 knockdown by using the IκB inhibitor (BAY 11–7082) and NF-κB JSH-23 or TNFR2 knockdown in KPC cells effectively decreased mRNA and protein levels of PD-L1. Antibodies against TNFR1 and TNFR2 were used in the presence of TNF-α. Anti-TNFR1 had no impact on PD-L1 protein levels, whereas anti-TNFR2 entirely eliminated *PD-L1* expression [175], demonstrating that TNFR2 is the major receptor via which TNF-α causes *PD-L1* up-regulation. Collectively, all of these data imply that TNFR2 may favorably up-regulate *PD-L1* after TNF-α engagement through activation of multiple signaling pathways, and that both NF-κB canonical and non-canonical pathways may be involved in this process. However, further research into the complex interactions between TNFR2-mediated NF-κB signaling and other signaling pathways that collectively up-regulate *PD-L1* in BC is strongly needed.

## Signals from TNFR2 activate various immuno-suppressive cells

The interaction of TNF-α with TNFR2 can result in the activation and differentiation of various immune cells whose normal function is to regulate excessive immune response; however, these mechanisms fail in many cancers, including BC.

### Signals from TNFR2 Activate T regulatory cells

Regulatory T cells (T-regs) are a specific type of T cells that are responsible for maintaining a state of immunological tolerance and preventing the autoimmune response that might result from an overreaction to self-antigens by CD4^+^ and CD8^+^ T cells. T-reg cells express high levels of the transcription factor forkhead box p3 (FoxP3), and IL-2 receptor alpha (CD25/IL-2R-α) chain [176]. T-regs not only inhibit CD4^+^ and CD8^+^ T cells, but also B cells, NK cells, natural killer T (NKT) cells, DCs, and macrophages [177]. In addition to FoxP3, T-regs express a variety of proteins in the cytoplasm and on the cell surface that contribute to and enhance their immunosuppressive function. One of the key protein receptors that significantly boost the immunosuppressive activity of Tregs is TNFR2. The first evidence to support this notion was proposed by Chen et al. [178]. They discovered that induced CD4^+^CD25^+^Tregs have higher surface TNFR2 expression than CD4^+^CD25¯Teffector cells (T-effs) in both resting and activated states. When co-cultured, in the presence of TNF-α, the inhibitory effect of T-regs on T-effs was temporarily abolished. However, prolonged TNF-α exposure augmented the inhibitory impact of TNFR2-expressing T-regs on T-effs. Surprisingly, TNF-α and IL-2 increased expression of FoxP3 and CD25 as well as STAT5 phosphorylation in T-regs, synergistically [179]. This suggests that TNF-α mediates these actions through its interaction with TNFR2, resulting in suppressive CD4^+^CD25^+^TNFR2^+^T-regs phenotype. Additionally, several studies have connected TNFR2 overexpression to an elevated suppressive function and enhanced phenotypic maturation in T-regs [179]. TNF-α/TNFR2 also promoted the differentiation, proliferation, and optimal activity of human Foxp3^+^T-regs in vivo and in vitro. T-regs deficient in TNFR2 are unable to differentiate or proliferate, leading to their loss of function. However, TNFR1-deficient T-regs showed no obvious alteration in their functions [180]. In line with this, MiR-125b-5p overexpression inhibited T-reg proliferation and suppressive function, by directly targeting TNFR2 on their surfaces [181]. However, this relationship between microRNA and TNFR2 up- or down-regulation requires more research. One study revealed that the lack of TNFR2 in T-regs did not decrease their number but rather diminished the expression and functional activity of FoxP3 of TNFR2^−/−^ mice compared to the wild type mice [182]. In all, T-regs are immunosuppressive cells by nature; the expression of TNFR2 on their surfaces dramatically boosted and strengthened their immunosuppressive capacity [183]. These findings show that TNF-α/TNFR2 axis is significantly important in increasing T-reg suppressive functions.

The presence of CD4^+^CD25^+^TNFR2^+^T-regs in certain TMEs now appears to be clinically significant and demonstrates potent immunosuppressive functions, facilitating tumor escape from the immune response. Recruitment and accumulation of T-regs is reported in various tumors, including gastric, pancreatic, colorectal, and lung cancers [184], and it is associated with poor prognosis. T-regs expressing TNFR2 were seen to have a strong suppressive effect in animal models of lung, colon, and hepatocellular malignancies [183, 185]. In BC, in contrast, T-regs were found to accumulate tremendously and were also associated with the aggressive TNBC phenotype [184, 186]. These accumulated Tegs are distinguished by the expression of genes such as *CXCR3*, *CX3CR1*, *CCR4*, *CCR5*, *CCR8*, *CCR10*, *ENTPD1*, *CD25*, *CD177*, *IL-1RL1*, *IL-1R2*, the *melanoma antigen family H1 gene* (*MAGEH1*), and *OX40* [186]. In addition, in stages I, II, and III of BC, patients had low accumulated levels of T-regs when compared with stage IV [184]. Moreover, the majority of CD4^+^TNFR2^+^ T-regs were negative for Foxp3 and CD25 in breast tumor-draining lymph nodes (TDLNs). It was also shown that the TNFR2^+^ cell ratio was considerably greater in CD4^+^CD25^+^FoxP3^+^T-regs than in CD4^+^CD25¯FoxP3¯, CD4^+^CD25¯FoxP3^+^, and CD4^+^CD25^+^FoxP3¯ T-regs [187]. In addition to these findings, a recent study suggested that TNFR2 was largely expressed in CD4^+^ T cells in the TDLNs of BC patients [188]. These reports were supported by the fact that anti-TNFR2 antibodies impaired the immunosuppressive function of TNFR2^+^Tregs on T-eff cells in BC via reducing FoxP3 overexpression [8]. Surprisingly, it seems that the dynamic expression of TNFR2 has a role extending much further than just the immunosuppressive function of T-regs. TNFR2 is crucial in maintaining FoxP3 expression by preventing DNA methylation at the *FoxP3* promoter region [182]. This increases enhancer of zeste homolog 2 (EZH2) expression [189], thereby exacerbating the immunosuppressive impact of T-regs. In this sense, there are a number of pathways necessary for T-regs to mediate their immunosuppressive role. One mechanism is through CD25. CD25 consumes IL-2, the essential cytokine for the proliferation and activation of T-eff cells, from the TME through high-affinity interactions with the cytokine. In addition, T-regs express tremendous quantities of IL-10, which down-regulates CD80 and CD86 on antigen-presenting cells (APCs), resulting in suppression. In addition, CTLA-4, a receptor highly expressed on T-regs, binds with high affinity to CD80 and CD86 on the surfaces of APCs to suppress CD28-induced co-stimulatory signaling [190]. Interestingly, FoxP3 regulates the surface expression of both CD25 and CTLA-4 on T-regs [190]. This suggests that TNFR2 may indirectly up-regulate CD25 and CTLA-4 by stabilizing FoxP3 and inhibiting its DNA methylation.

T-regs may express considerable levels of IL-10, IL-35, TGF-β, Galectin, NRP, Granzyme-B, and cAMP in order to sustain their suppressive activity [177]. In addition, T-regs can induce T-eff cell apoptosis through the activation of Bim, a critical pro-apoptotic protein [184]. One way in which Bim can also be induced is by TNFR2-mediated MEK/ERK activation [88]. This indicates that, depending on TNFR2 status, various mechanisms can interact with each other to increase the immunosuppressive effect of T-regs. Several signaling pathways have been proposed to mediate the function of TNFR2 signaling in T-regs, including NF-κB, MAPK (ERK1/2, p38, and JNK), and PI3K/Akt. In particular, NF-κB signaling components like NF-κB-p65, c-Rel, RelB, and IKKs, support T-reg formation and maintain their homeostasis. NF-κB-p65, for example, regulates the development of T-regs. While c-Rel, plays a crucial role in the homeostasis of T-regs during cancer response. c-Rel was also discovered to stimulate FoxP3 during the thymic growth of T-regs; however, this action can prevented in order to preserve their suppressive activity, following maturation [191]. FoxP3 interacts with the c-Rel N-terminal domain, resulting in the deactivation of NF-κB. Tianzhen et al*.* [191] found that mice lacking c-Rel had a decreased number of naturally occurring T-regs (nT-regs), indicating that c-Rel, along with other NF-κB components, plays an essential role in inducing *de-novo* FoxP3 expression. Pentoxifylline-mediated inhibition of c-Rel also resulted in T-reg function suppression. Additionally, animals deficient in p100, a normal RelB suppressor, exhibited reduced T-reg function [192]. In addition to c-Rel, other NF- κB components, such as IKK-α and IKK-β, also influence T-reg production, phenotypic stability, and optimal activity [191]. These reports clearly show that TNFR2-mediated NF-κB canonical and non-canonical activation is vital for T-reg immunosuppressive functions; however, more studies are needed to fully elucidate the mechanism of the TNFR2 signaling pathway in T-regs.

As previously discussed, TNFR2 can activate a number of MAPK signaling proteins such as ERK, JNK, and p38, which collectively aggregate and activate different transcription factors (TRAF2, ATF-2, RIP, TAK1, and MEKK1, 3, 6, and 7), resulting in highly suppressive TNFR2^+^Tregs. In fact, the MAPK signaling pathway can act independently of NF-κB to induce the development, proliferation, expansion, and activation of TNFR2^+^Treg cells through these transcription factors. [191]. One such transcription factor, activating transcription factor 2 (ATF-2), can be phosphorylated by ERK, JNK, and p38 allowing it to bind to DNA to induce cytokine production (IL-1β, IL-6, and TNF-α), and cell motility [193]. ATF-2 has a DNA binding element, called the CRE/ATF-2, which has a binding site on the *cyclin D1* promoter (Fig. 6) [194]. After binding, ATF-2 activates the *cyclin D1* gene and promotes T-reg development by stimulating other transcription factors [195]. It has been suggested that the presence of TNF-α in the TME after immunotherapies like toll-like receptor agonists, DC vaccines, and tumor vaccines might elevate the expression of TNFR2 on T-regs, thereby boosting their ability to suppress anti-cancer immune responses [196]. Moreover, the PI3K/Akt pathway seems to play a significant role in T-reg maintenance. As mentioned previously, TNFR2 can stimulate this pathway. T-reg cells have been found to suppress the PI3K/Akt pathway and its downstream target mTORC1 in order to switch off glycolysis and use fatty-acid oxidation (FAO)-fueled oxidative phosphorylation (OXPHOS) for their development and suppressive function [197]. However, signals from TNFR2 co-stimulation can re-activate the PI3K/Akt/mTORC1 pathway and induce NF-κB in T-reg cells, forcing them to utilize glycolysis for their phenotypic maturation and activity [197]. Additionally, TNFR2 co-stimulation with CD3 or CD28 significantly increases the expression of several genes, including FoxP3 and CD25 [197]. TNFR2 is necessary for persistent activation of Akt and NF-κB in response to TCR/CD28 co-stimulation [198]. In contrast, a different research group showed that down-regulation of FoxP3 is caused by TNF-α-TNFR2-mediated activation of Akt and suppression of the TGFβ/Smad3 signaling pathway [199]. However, further research is needed to fully understand the role of TNFR2/PI3K/Akt in the biology of T-regs in cancer, specifically BC.

**Fig. 9:**
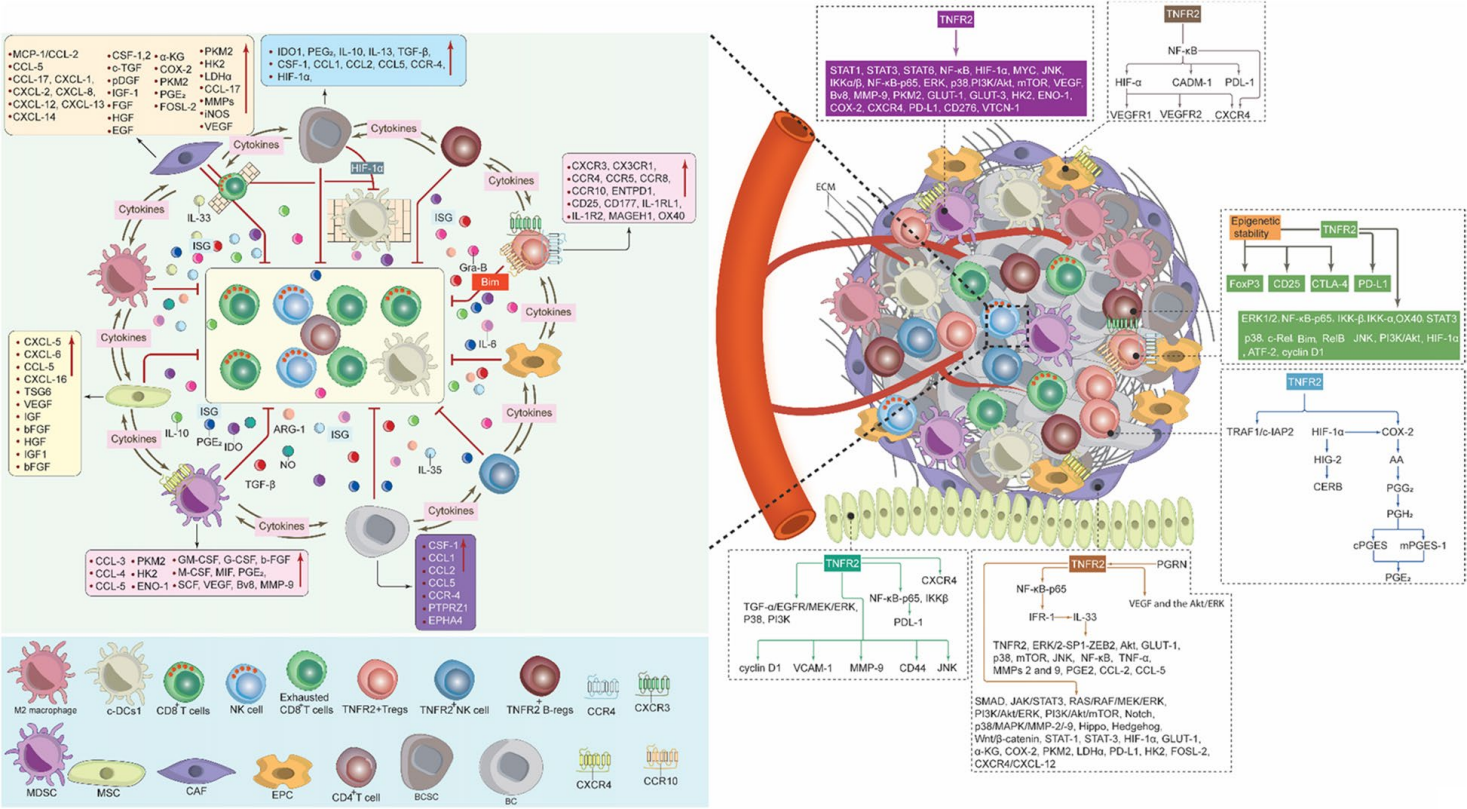
TNFR2 expression in BC TME. TNFR2^+^ cells secrete IL-10, IL-4, IL-6, IL-33, IL-35, TGF-β, HIF-1α, IDO, COX-2/PGE_2_, ARG-1, iNOS, and others, establishing the ISG. This ISG depletes essential amino acids, impairs MHC class I antigen presentation, leading to suppression of DC1s, CD8^+^ T cells and promoting CD8^+^ T cell exhaustion. COX-2/PGE_2_ inhibits NK cell effector receptors and induces M2 macrophage differentiation, further enhancing immunosuppression. Hypoxia-induced HIF-1α supports BC metabolism and immunosuppressive cells, inhibits NK cell cytotoxicity, and promotes MDSC differentiation, as well as induces the activation of COX-2/PGE_2_, IL-6, IL-10, and TGF-1β, leading to PD-L1 overexpression, which inhibits c-DC1s. TNFR2 also induces the expression of immunosuppressive receptors and stabilizes FoxP3. In CAFs, TNFR2 activation leads to stromal mass formation, hindering CD8^+^ T cell infiltration, and also augments AICD- and FAS-mediated cell death. TNFR2-mediates the expression of chemokines, and their receptors recruit c-DC1s to BC TME, initiating their suppression and affecting CD8^+^ T cell stimulation. Wnt/β-catenin downregulates CCL-4, inhibiting c-DCs1 migration, recruitment, and tumor infiltration. Moreover, increased BC cell mass and reduced blood vessel formation act as physical barriers, hindering the infiltration and recruitment of c-DC1 cells into the TME. CXCR-3^+^T-regs compete with CD8^+^ T cells for the CXCL-9 gradient, restricting their activation by c-DCs1. Immunosuppressive cells can also mediate cell-to-cell contact inhibition via PD-L1, PD-1, FAS, and CTLA-4 expression. Collectively, this can lead to dysfunctional c-DC1s, ultimately limiting their presence in the TME and supporting BC growth. TNFR2, tumor necrosis factor receptor type two; Mb-TNF-α, membrane-bound tumor necrosis factor; c-DC1, type 1 conventional dendritic cells; BC, breast cancer; BCSCs, breast cancer stem cells; IL-4, interleukin 4; IL-6, interleukin 6; IL-10, interleukin 10; IL-12, interleukin 12; IL-33, interleukin 33; IL-35, interleukin IL-35; TGF-β, transforming-growth factor β; COX-2, cyclooxygenase-2; HIF-1α, hypoxia inducible factor 1 alpha; PEG_2_, prorstaglandin-E2; ARG-1, arginase-1; MMP-9, matrix metalloproteinase-9; CCL-4, chemokine (C–C) motif ligand 4; CCL-19, chemokine (C–C) motif ligand 19; CCL-21, chemokine (C–C) motif ligand 21; CXCR3, C-X-C chemokine receptor 3; CXCR4, C-X-C chemokine receptor 4; XCR1, X-C motif chemokine receptor 1; CCR-5, C–C chemokine receptor type 5; CCR-7, C–C chemokine receptor type 7; IFN-γ, interferon gamma; EGFR, epidermal growth factor receptor; PD-1, programmed cell death 1; PD-L1, programmed cell death ligand 1; CTLA-4, cytotoxic T lymphocyte antigen 4; VEGF, vascular endothelial growth factor; MHC-1, major histocompatibility complex class I; ISG, immunosuppressive gradient; STAT3, signal transducer and activator of transcription 3; STAT5, signal transducer and activator of transcription 5; c-MYC; cellular myelocytomatosis; GLUT-1, glucose transporter 1; GLUT-3, glucose transporter 3; α-KG, alpha ketoglutarate; PKM2, pyruvate kinase M2; ENO-1, enolase-1; LDHα, lactate dehydrogenase-alpha; IDO, indoleamine 2,3-dioxygenase; CSF-1,2, macrophage colony-stimulating factor 1, 2; c-TGF, connective-tissue growth factor; pDGF, platelet-derived growth factor; IGF-1, insulin-like growth factor 1; FGF, fibroblast growth factor; HGF, hepatocyte growth factor; EGF, epidermal growth factor; bFGF, basic insulin-like growth factor; G-CSF, granulocyte-colony stimulating factor; M-CSF, macrophage colony stimulating factor; MDSCs, myeloid-derived suppressor cells; T-regs, T regulatory cells; B-regs, B regulatory cells, CAFs, cancer associated fibroblast; EPCs, endothelial progenitor cells; MSCs, mesenchymal stem cells; TME, tumor microenvironment; ECM, extracellular matrix; AICD, antigen-mediated activation-induced cell death

Considering the nature of BC as a solid tumor, T-regs can successfully exploit the hypoxic condition to activate various genes. As such, HIF-1α activation plays an important role in this process. For example, HIF-1α can induce the expression of VEGF [200], CXCR4/CXCL12, and CCL8/CCR10 [201], which together mediate the recruitment of T-regs in BC TME. Also, CXCR4, and HIF-1α are known to be activated by TNFR2, thus maximizing T-regs suppressive function. Furthermore, TNFR2 is involved in the activation and phosphorylation of STAT3. STAT3 positively controls FoxP3 expression, which can boost T-reg formation and their suppressive function via IL-10 and IDO production (Fig. 9) [202]. TNFR2 expression on T-regs also promotes the production of various anti-apoptotic proteins (Bcl-2, Bcl-XL, Bcl-W, c-IAP1/2, A1/BFL-1, Cyclin D1, and MCL-1), leading to Tregs-mediated apoptosis resistance, extending their survival in BC TME. In addition to CD4^+^CD25^+^T-reg cells, TNFR2 may activate CD8^+^CD25^+^T-reg cells. In this manner, TNF-α is observed to increase the proliferation, CD25 up-regulation, and immunosuppressive activity of CD8^+^FoxP3^+^T-regs, in response to CD3 antibodies. Anti-TNF-α antibodies were also seen to reverse this action, corroborating the idea that TNF-α can enhance CD8^+^CD25^+^T-regs. Similarly, TNFR2 activated FoxP3 and transformed CD8^+^T cells into CD8^+^FoxP3 + T-regs in the presence of anti-CD3/CD28 beads [203]. In fact, CD8^+^CD25^+^T-reg can also be triggered by physical contact (Mb-TNF-α/ TNFR2 and CD80/CD28) between CD8^+^T cells and monocytes, converting CD8^+^T to CD8^+^CD25^+^T-reg. This effect was decreased by the combined inhibition of TNF-α and CD86 [203]. Likewise, evidence suggest that CD8^+^T-regs expressing high amounts of TNFR2 are the most potent T-regs [204]. Signals via TNFR2 on the CD8^+^CD25^+^T-reg boosted PD-L1 synthesis, augmenting their suppressive activity. Moreover, TNFR2 is currently more effective than CD25 as a functional biological marker for identifying CD8^+^FoXP3^+^T-regs (Fig. 9) [203]. Importantly, T-reg cells with elevated CTLA-4, PD-L1, and TNFR2 expression seem to have a highly immunosuppressive phenotype in the BC TME; functional elimination or activity reversal of these cells could provide a promising treatment strategy for BC. This also opens up the door to the use of TNFR2 agonistic antibodies as potential regulators of different autoimmune diseases.

### Signals from TNFR2 activate B regulatory cells

Regulatory B cells, also known as B-regs, are the family of B cells that help to regulate the immune system [205]. They have been associated with inflammation control, autoimmunity prevention, and cancer progression. B-regs can inhibit different immune cells, including CD4^+^ T cells, CD8^+^ T cells, NK cells, NKT cells, DCs, and macrophages, by producing anti-inflammatory chemicals such as IL-10, IL-35, and TGF-β [205]. Furthermore, they may aid in the conversion of naive T cells into CD4^+^FoxP3^+^ T-regs through a mechanism involving TGF-β, thus weakening the immune system's response to malignancies [205]. Interestingly, independent of TGF-β, B-regs are still able to suppress cytokine secretion of CD4^+^ T cells through a mechanism dependent on CD80/ CD86, and IL-10, as well as increase the production of CTLA-4 and FoxP3 proteins in T-regs [205]. Importantly, B-reg cells are now implicated in a variety of tumors, including BC [206–209]. Indeed, these data indicate that B-regs may collaborate with T-regs to suppress the immunological response in the BC TME. However, the immunosuppressive action of B-regs might be greatly amplified by the presence of additional variables. It appears that the TNF-α/TNFR2 interaction may play an important role in enhancing the immunosuppressive effects of B-regs in the TME of BC. In vitro, analysis of the cytokine profiles of human naïve B cells, CD24^hi^CD38^hi^ transitional B cells (TrBs), and CD24^hi^CD27^+^ cells found that TNF-α and IL-10 were highly expressed by these cells. Relative to other cells, TrBs showed a greater TNF-α/IL-10 ratio and successfully suppressed T helper 1 cytokine release [210]. To add to this, anti-IL-10 significantly reduced this inhibition, while anti-TNF-α increased naïve B cells and CD24^hi^CD27^+^ suppressive activity [210], suggesting that the TNF-α/IL-10 ratio is more important in indicating regulatory function than IL-10 alone. Conversely, CD24^hi^CD27^+^ cells were found to suppress monocyte-mediated TNF-α production through an IL-10-dependent mechanism (Fig. 9) [205]. A study by Ammirante et al*.* also showed that when lymphocytes, including B cells, infiltrate tumors, they tend to produce higher levels of cytokines like IL-6 and TNF-α. It is thought that IKK-α and STAT3 signaling pathways may play a significant role in this phenomenon [211]. However, this study did not show which phenotype of B cells is engaged in this action. In a study using mice, researchers found that the development of skin cancer induced by chemicals DMBA and TPA was reduced when B cells were absent from these mice [212]. However, when B cells were introduced to these mice, the cancer growth was partially restored. Further experiments revealed that the introduction of B cells alone could not promote the growth of skin cancer in mice that lacked TNF-α. Additionally, the number of B-reg (CD19^+^CD21^+^IL-10^+^) cells was found to be significantly reduced in TNF-α-deficient mice when compared to wildtype mice[212]. These findings suggest that B-reg cells contribute to the growth of cancer, and that TNF-α may play a role in the generation and accumulation of these cells within tumors. Recent research has found that most IL-10-producing B cells also produce TNF-α and IL-6 across various phenotypes. When purified and re-stimulated, these B cells lost their capacity to produce IL-10, whereas non-IL-10 producing B cells can produce it after the second stimulation [213]. These data suggest that there may not be a dedicated B-reg cell lineage, but rather that B cells have the potential to produce regulatory cytokines depending on the stimulation they receive. Based on this information and the fact that TNF-α was shown to be a key element of how B-regs suppress the immune system, it is likely that TNFR2 is involved in this process. One research group found that activating human IL-10^+^B cells with the Toll-like receptor-9 (TLR-9) agonist CPG ODN dramatically boosted TNFR2 cell surface expression, leading to the formation of IgM^+^TNFR2^+^CD19^+^B-regs. TNFR2 expression was associated with higher IL-10 production and cell survival. It is believed that this activity was initiated by direct interaction between Mb-TNF-α and TNFR2 on B-regs surface [214]. It has also been proven that TNFR2-expressing B-regs inhibit CD4^+^ and CD8^+^ T cells by secreting IL-10 in response to TLR-9 stimulation [215].Although IL-10 was responsible for the suppressive action of TNFR2^+^B-regs, it is believed that additional pathways are also involved, including cell–cell contact through CD80/CD86, CD73, PD-L1, CTLA-4, Fasl, intercellular adhesion molecule-1 (ICAM-1), and Mb-TNF-α, as well as secretion of inhibitory cytokines (TGF-β, IL-35,) IDO, and Granzyme-B (Fig. 9) [215, 216]. The TLR-9 agonists CPG and CD40L activate STAT3, CREB, p38-MAPK, ERK1/2, and PI3K which leads to up-regulation of IL-10 in B-regs. It has also been shown that aryl-hydrocarbon receptor (AhR) and TLR interactions cause CD19^+^CD21^hi^CD24^hi^ B-regs to overexpress IL-10, thus inhibiting the development of GC B cells and plasma cells as well as a number of pro-inflammatory mediators [213]. This process is mediated via c-Maf stimulating the production of IL-10 though pathways involving interaction with AhR. These results together demonstrate that TNFR2 has the capacity to improve the suppressive effect of B-regs in BC and that TNFR2 expression could be a marker to identify, define, and purify B-reg cells. However, more research is needed to determine the exact source of the TNFR2 signal that enhances the suppressive functions of B-reg cells; one potential candidate might be Mb-TNF-α expressed on BC cells or other immune cells interacting with TNFR2 on the surface of the B-regs.

### Signals from TNFR2 activate myeloid-derived suppressor cells

Myeloid-derived suppressor cells (MDSCs) are a heterogeneous population of immature granulocytic and monocytic myeloid cells that arise in response to aberrant inflammatory conditions, including cancer, and are one of the primary sources of immunosuppression in the TME [217]. Interestingly, BC cells contribute to the establishment of MDSCs by influencing hematopoiesis and myeloid cell differentiation by secreting various cytokines–for example, granulocyte–macrophage colony stimulating factor (GM-CSF) [218], in addition to granulocyte-colony stimulating factor (G-CSF), M-CSF, macrophage migration inhibitory factor (MIF), PGE_2_, stem cell factor (SCF), VEGF, TGF-1β, IL-1β, IL-6, and IL-10 (Fig. 9) [219, 220]. In fact, the recruitment of human MDSCs into the BC TME is linked to a worse prognosis, worse clinical outcomes, treatment resistance, and profound immunosuppressive activities [219]. The MDSCs, in synergism with T-regs and B-regs, produce molecules that effectively suppress CD4^+^ T cells, CD8^+^ T cells, NK cells, NKT cells, and APCs, as well as supporting BC TME development [220]. Several molecules and events, including arginase-1 (ARG-1), inducible nitric oxide synthase (iNOS), IDO, reactive oxygen species (ROS), nitric oxide (NO), TGF-1β, IL-10, COX-2, STAT1, STAT3, STAT6, NF-κB, HIF-1α, endoplasmic reticulum stress pathway and down-regulation of L-selectin expression in T cells, facilitate the suppressive role of these cells [217, 220–222]. More specifically, following ARG-1 secretion by MDSC, the TME is depleted of L-arginine and cysteine amino acids, leading to CD3-ζ-chain inhibition in the TCR complex. Likewise, iNOS catalyzes the conversion of L-arginine and NADPH to produce citrulline and NO. These processes suppress anti-tumor CD8^+^ and CD4^+^ T cells by inhibiting the TCR (Fig. 9) [223]. NO production in the TME can impair major histocompatibility complex (MHC) class I presentation of tumor antigens on the surface of BC cells, thereby hindering CD8^+^ T cell and NK cell responses by impeding antigen presentation. IDO, on the other hand, inhibits CD8^+^ T cells by disrupting tryptophan metabolism through the L-kynurenine pathway (Fig. 9) [223]. Tumor-associated DCs can use the same approach to inhibit CD8^+^ T cells [224]. In addition, COX-2-mediated activation of PGE_2_ induces differentiation of M2 macrophages from M1 macrophages in the BC TME leading to enhanced immunosuppression [225], indicating a reciprocal activation between MDSCs and M2 macrophages. Furthermore, by secreting VEGF, Bv8, MMP-9, and basic fibroblast growth factor (b-FGF), MDSCs play an important role in TME reorganization, cancer angiogenesis, metastasis, and the formation of pre-metastatic niches, hence regulating cancer initiation and progression (Fig. 9) [219, 221]. Similarly, TNF-α can be regarded as an additional factor that can promote MDSCs suppressive impact. There is evidence that TNF-α inhibits MDSCs’ complete maturation and enhances their suppressive effects by increasing their accumulation and controlling the interaction between RAGE and its ligands S100A8/9 [222, 226]. Moreover, TNFR2 signals were essential for this process [226]. Further solidifying the role of TNFR2 in MDSC-mediated immunosuppression. TNFR2-mediated NF-κB activation supported MDSCs' apoptotic resistance. In addition, the levels of phosphorylated IKKα/β, NF-κB-p65, and IκBα proteins were significantly elevated after the administration of TNF-α. In conjunction with this, the increase in NF-κB subunits prevented caspase-8 cleavage and was substantially correlated with increased c-LIP levels [226]. Mb-TNF-α-mediated TNFR2 activation, but not s-TNF-α, was observed to activate and improve MDSCs suppressive action. Activation of TNFR2 by Mb-TNF-α boosted NF-κB-p65 nuclear translocation and promoted IκBα degradation, triggering the p38 signaling pathway. However, Mb-TNF-α showed no discernible effect on the ERK1/2 and JNK signaling pathways after stimulating TNFR2 [227]. Later evidence supports the notion that Mb-TNF-α is indeed involved in MDSCs infiltration to the tumor site, as TNFR2 stimulation by Mb-TNF-α up-regulates several chemokines and chemokine receptors. In line with this, TNFR2 stimulation has been observed to stimulate CXCR4 over-expression in MDSC cells following interaction with Mb-TNF-α [228]. CXCR4 overexpression were partly suppressed by inhibiting the NF-κB and p38 signaling pathways with pyrrolidine dithiocarbonate and SB203538, respectively. Moreover, PGE_2_ can also stimulate CXCR4 up-regulation in MDSCs [228], indicating a potential correlation between Mb-TNF-α/TNFR2/NF-κB/p38 activation and PGE_2_, ultimately leading to CXCR4 over-expression.

It seems that a majority of TNFR2-mediated signaling pathways support MDSC metabolism, maintenance, apoptosis resistance, and immunosuppression. Hypoxia, for instance, has been shown to promote MDSCs differentiation and activation [229]. This is substantiated by the fact that HIF-1α boosts ARG-1 and iNOS expression, transforming MDSCs into antigen-nonspecific suppressors of T cell activity (Fig. 9) [230]. A similar effect was observed with HIF-1α-induced miR-210 up-regulation [231]. In further support of MDSC suppressive function, HIF-1α triggered *PD-L1* over-expression on MDSC surfaces [232]. HIF-1α, through its interaction with MYC, STAT3, and STAT5, up-regulates the expression of genes encoding proteins that aid in the aerobic glycolysis of MDSCs, including PKM2, GLUT-1, GLUT-3, hexokinase-2 (HK2), and enolase-1 (ENO-1) (Fig. 9) [233]. Thus, given the hypoxic nature of BC as a solid tumor, MDSCs are believed to be particularly active inside the TME, where they produce a significant amount of VEGF and other cancer-promoting molecular species in response to HIF-1α (Fig. 9). In addition to the role of HIF-1α, TGF-1β was shown to up-regulate miR-494, facilitating MDSC invasion and metastasis. Overexpression of miR-494 suppressed PTEN, which in turn stimulated the accumulation of MDSCs through CXCR4 and activation of the PI3K/Akt, mTOR, and NF-κB pathways, all of which enhanced the expression of multiple MMPs, supporting BC development [234]. This highlights the potential for a possible correlation between HIF-1α, micro-RNA, and TNFR2 with MDCS recruitment and immunosuppressive maturation.

It also seems that additional immunosuppressive cells are impacted by TNFR2-mediated MDSCs activation. For instance, it was shown that MDSCs secrete TGF-1β, IL-10, and IFN-γ, encouraging the *de-novo* development of FoxP3^+^ T-regs when studied in vitro. In addition, T-regs have been shown to improve MDSCs’ activity by up-regulating the expression of *PD-L1*, *CD276*, and *V-set domain-containing T-cell activation inhibitor-1* (*VTCN-1*) (Fig. 9) [220]. It has also been observed that inter-tumoral MDSCs produce high amounts of CCL-3, CCL-4, and CCL-5, which can attract CCR5^+^ T-regs to the tumor site. Moreover, it was discovered that MDSCs bolster the generation of highly immunosuppressive M2 macrophages, which have poor IL-12 expression [220]. Collectively, TNFR2^+^ MDCS targeting may provide a novel strategy for BC treatment. Indeed, TNFR2^+^ T-regs, TNFR2^+^ B-regs, and TNFR2^+^ MDCSs all seem to be able to reciprocally activate one another, releasing a variety of immunological mediators, thus inducing a highly immunosuppressive TME, supportive of BC proliferation and development.

### TNFR2 expression in other immune cells

The TNFR2 receptor exhibits high expression levels in various other immune cells, thereby resulting in an augmented TME with immunosuppressive characteristics. These cells are further discussed in the subsections that follow.

#### Natural killer cells (NK cells)

NK cells play a critical role in controlling tumor growth in the TME through direct attacking cancer cells or by secreting cytokines like IFN-γ. NK cells expressing TNFR2 were found to exert a strong immunosuppressive effect in the TME via the TRAF1/c-IAP2 signaling pathway, which is activated through the interaction of TNF-α and TNFR2, thereby inhibiting the NK cell activating receptor, natural cytotoxicity triggering receptor 1 (NCR1) [235]. This interaction also influences the NK cell cytokine profile via the expression and secretion of IL-4, IL-10, and IL-13 as well as the abolition of NK cells’ cytotoxic effect [235]. Contrary to this notion, the effect of TNFR2 expression on the NK cell surface increased the secretion of IFN-γ [236], in addition to enhancing proliferation and metabolic activity via the TNF-α/TNFR2/IL-18/MyD88 pathway [237]. Additionally, the interaction between TNF-α and TNFR2 through direct cell–cell contact plays a crucial role in the ability of DCs to stimulate NK cell activation [238]. Likewise, Mb-TNF-α was found to activate and boost TNFR2^+^ NK cell activity, resulting in increased GM-CSF expression [239]. It was also discovered that NK cells upregulate the expression of TNFR2 in cancer cells although this mechanism is still elusive and requires more investigation [240]. Furthermore, NK cells were found to exhibit high levels of Mb-TNF-α constitutively [241], suggesting that BC cells may augment the immunosuppressive impact of NK cells by direct interaction between TNFR2 on BC cells and Mb-TNF-α on NK cells. Notably, TNFR2-mediated activation of HIF-1α can suppress NK cell function through different mechanisms. HIF-1α was observed to inhibit the expression of NKG2D and MHC class I chain-related polypeptide A and B (MICA/B). Loss of MICA leads to a decrease in NK cell cytotoxicity, impairment of antigen-MHC-1 presentation recognition, and inhibition of NK cell/T cell activation. Furthermore, HIF-1α was found to induce the expression of its downstream target gene, hypoxia-inducible gene-2 (HIG-2) (Fig. 9) [242]. The activation of HIG-2 subsequently triggers AMP-activated protein kinase (AMPK), resulting in the phosphorylation of CERB. This process ultimately leads to the over-expression of IL-10, thereby inhibiting the effective cytotoxic tumor-killing ability of NK cells [242]. Additionally, BC cells and TME cells can secrete COX-2 via TNFR2-mediated pathways such as HIF-1α, which can suppress NK cells. In this scenario, NK cells demonstrate elevated levels of prostaglandin E2 receptors (EP; 1–4), also known as PGE_2_ receptors. These receptors facilitate the binding of PGE_2_ and subsequently mediate its physiological effects [243]. More specifically, COX-2 catalyzes the conversion of arachidonic acid (AA) to prostaglandin G2 (PGG_2_) and subsequently to prostaglandin H2 (PGH_2_), serving as precursors for prostaglandins. PGH_2_ is then transformed into PGE_2_ by cytosolic PGE_2_ synthase (cPGES) and membrane-bound PGE_2_ synthase 1 (mPGES-1) [244]. In the BC TME, PGE_2_ will selectively bind to EP-4 receptors on NK cells. This binding results in the suppression of NK cell migration, cytotoxic activity, and the release of IL-12 and IFN-γ [245]. PGE_2_ inhibits NK cell effector receptors, such as NCR2/NKp44, NCR1/NKp46, NCR3/NKp30, NKG2D, and CD16 (Fig. 9) [243], leading to impaired function and promoting tumor immune evasion. However, further investigations are needed to fully elucidate the role of the TNFR2/COX-2/PGE_2_ signaling pathway in NK cell inhibition and immune evasion dysfunction within the BC TME. Indeed, understanding how TNFR2 contributes to NK cell activation and/or exhaustion presents an opportunity for further research into potential therapeutic strategies to enhance the anticancer activity of NK cells. Studying the relationship between TNF-α/TNFR2 and signaling pathways like COX-2 and HIF-1α in relation to the NK cell biology also presents a fascinating research opportunity to gain insight into the NK cell function.

#### Mesenchymal stem cells (MSCs)

MSCs are a type of self-renewing cells that can be found in multiple tissues throughout the body. These cells are also present in the TME where they function as cancer-resident MSCs, thereby facilitating the progression of tumors [246]. Mechanistically, in BC, MSCs have the ability to stimulate the maturation and specialization of M2-polarized macrophages and MDSCs from monocytic myeloid-derived suppressor cells and monocytic myeloid-derived DCs, respectively, both of which are known for their potent immunosuppressive properties [247, 248]. This process occurs through the MSCs releasing of exosomes containing semaphorins, TGF-β, and complement C1q, as well as secreting growth-regulated onco-proteins (GROs) such as CXCL-1/GRO-α, CXCL-2/GRO-β, and CXCL-3/GRO-γ, ultimately leading to the up-regulation of *PD-L* (*1* and *2*), increased ARG-1 and iNOS activity, and the induction of IDO, PGE_2_, IL-1β, IL-4, IL-6, COX-2, MMP-9 and IL-10 production via the TME cellular components (Fig. 9) [247, 248]. Similarly, MSCs can express a diverse repertoire of chemokine and cytokine receptors that recognize CXCL-8, along with IL-1β and TNF-α [249]. MSCs exhibit elevated expression of IL6R/gp130, enabling them to exploit the IL-6 gradients produced by BC and other cells to migrate toward the TME. Once there, MSCs secrete CXCL-17, which in conjunction with IL-6, contributes to the development of chemoresistance in BC [246]. Interestingly, the TNF-α/TNFR2 signaling pathway was found to induce activation of NF-κB-p65 and IKKβ, leading to the up-regulation of cyclin D1, VCAM-1, MMP-9, and CD44, which ultimately promotes the migration and invasion of MSCs. MSCs express both TNFR1 and TNFR2 receptors but do not undergo apoptosis upon exposure to TNF-α. This indicates that the TNF-α/TNFR2 signaling pathway is likely involved, rather than TNF-α/TNFR1 [250]. Recent data indicates that MSCs possess immunosuppressive properties through the activation of T-regs. This immunosuppressive effect is achieved by inhibiting T cell proliferation, activation, and cytokine production. Importantly, this is mediated by TNF-α/TNFR2 interaction [251]. Furthermore, activation of the TNF-α/TNFR2 signaling pathway results in a significant enhancement of MSCs stability, phenotypic characteristics, migratory function, and iNOS production capacity. These cells can stimulate and initiate the development of T-reg cells which together will collaborate to produce various immunosuppressive mediators including CXCL-5, CXCL-6, IL-10, PGE_2_, TGF-β, TSG6, and other supporting growth factors like VEGF, IGF1, basic fibroblast growth factor (bFGF), and HGF, all of which effectively inhibit the anti-tumor immune response [251–253]. Alternatively, MSCs are consistently attracted to the TME via various signaling molecules such as VEGF, IGF1, bFGF, HGF, CCL-5, CXCL-16, and TGF-β, which are produced by immunosuppressive cells, cancer-associated fibroblasts (CAFs), and BC cells (Fig. 9) [254]. In fact, the activation of TNFR2 by TNF-α was shown to lead to the stimulation of IKKβ, which subsequently caused the translocation of NF-κB-p65 into the nucleus, in a study [250]. This then triggered the expression of several onco-proteins, including CD44, JNK, VCAM-1, and MMP-9, resulting in the proliferation and invasion of MSCs. However, this effect was efficiently inhibited through treatment with a dominant-negative mutant of IKKβ (dnIKKβ) [250], indicating the importance of the NF-κB-IKKβ canonical pathway for MSC proliferation, migration, and invasion. In addition, TNF-α/TNFR2 interaction facilitated activation of the P38, PI3K, TGF-α/EGFR/MEK/ERK, and NF-κB signaling pathways, resulting in enhanced MSCs cell survival, angiogenesis, and immunosuppression via the production of HGF, VEGF, and MMP-9 [250, 255, 256]. Thus, the functions of TNFR2^+^MSCs go beyond their direct immunosuppressive activity in the TME to indirectly trigger and activate numerous immunosuppressive cells, enabling BC cell immune escape, proliferation, progression, and treatment resistance. As a result, targeting TNFR2^+^ MSCs may aid in the development of successful new BC therapies. In addition, TNFR2 could be used as a potential marker for functional isolation and characterization of MSCs.

#### Endothelial progenitor cells (EPCs)

EPCs are a subset of undifferentiated endothelial cells (ECs) that are pivotal in the maintenance of endothelial homeostasis and neo-angiogenesis. These cells possess the capacity to incorporate vascular structures at sites of injury, thus maintaining the integrity of the vasculature [257]. In BC, EPCs that express VEGFR1, VEGFR2, and CXCR4 are attracted to the tumor site due to estrogen-mediated HIF-α upregulation. This upregulation leads to the overexpression of tumorigenic molecules, including VEGF and CXCL-12, as well as stimulation of the PI3K/Akt/ERK signaling pathway [258]. This enhances the tumor vasculogenic process that is required for BC progression and development. In fact, CAFs are believed to recruit EPCs to BC TME via the secretion of massive amounts of CXCL-12, which interact with CXCR4 on EPCs, promoting tumor angiogenesis after differentiation into tumor-associated vascular endothelial cells (TAVECs) [259]. Although these reports clearly demonstrated the role of EPCs in BC development, no information is mentioned regarding their immunosuppressive contribution to BC TME.

More recent evidence indicates that EPCs may exert potent immunosuppressive effects within the TME, with the TNF-α/TNFR2 signaling pathway playing a crucial role in this mechanism. In fact, it was believed that TNF-α/TNFR2 interaction promotes EPC activation, mobility, and recruitment by upregulating NF-κB and cell adhesion molecule 1 (CADM-1) [260]. As previously stated, NF-κB signaling pathway activation through TNFR2 in EPCs is suggestive of immunosuppressive properties, as NF-κB is known to trigger and activate a range of proteins, including PDL-1, TGF-β, HIF-α, IL-6, and IL-10, among others (Fig. 9). This was supported by the finding that, in a co-culture model, TNF-α/TNFR2 signaling was necessary for EPCs to suppress the activation and proliferation of CD4^+^ and CD8^+^ T lymphocytes [257]. Additionally, after interacting with CD4^+^ and CD8^+^ T cells, TNFR2^+^EPCs produced significant amounts of HLA-G, IL-10, and TGF-β. Anti-TNFR2 antibodies abolished the inhibition of CD4^+^ and CD8^+^ T lymphocytes and dramatically reduced the concentrations of HLA-G, IL-10, and TGF-β, demonstrating its role in this process [257]. Unsurprisingly, EPCs deficient in Mb-TNF-α were unable to suppress CD4^+^ and CD8^+^ T lymphocytes [257], indicating that cell–cell contact via Mb-TNF-α/TNFR2 is required for the inhibitory effect of EPCs on CD4^+^ and CD8^+^ T lymphocytes. In addition to this, a recent study found that the addition of TNF-α to the media increased TNFR2 expression on EPCs, resulting in improved immunosuppression. TNFR2^+^EPCs co-cultured with CD4^+^ and CD8^+^ T cells in the absence of TNF-α exhibited significant inhibition of CD4^+^ and CD8^+^ T lymphocytes [261]. These data suggest that both forms of TNF-α can elicit the immunosuppressive function of EPCs. This also indicates that BC cells can stimulate EPCs via two mechanisms: one involving Mb-TNF-α/TNFR2-mediated cell–cell interaction, and the other the secretion of s-TNF-α. In summary, TNFR2^+^EPCs seem to play a critical role in the immunosuppressive TME of BC; however, additional research is necessary to understand the involvement of TNF-α/TNFR2 signaling and associated pathways.

#### *Cancer* associated fibroblast (CAF)

CAFs are a distinct cellular entity that exhibits significant diversity and constitute the predominant cellular component within the TME [262, 263]. Although the specific origin of these cells is unknown, recent research suggests that they might include typical resident tissue fibroblasts, MSCs, epithelial cells, endothelial cells, and less frequent cells such as stomach muscle cells, adipocytes, and pericytes [263]. Under normal physiological conditions, fibroblasts differentiate into myofibroblasts expressing alpha smooth muscle actin (α-SMA) during tissue fibrotic scarring or wound healing. During wound healing, myofibroblasts modify the ECM by producing the uPA, MMPs, ECM proteins, and tissue inhibitors of metalloproteinases (TIMPs). Additionally, these cells generate specific mediators to interact with and regulate adjacent cells [262, 263]. As a result, CAF cells in the BC TME are characterized as activated myofibroblasts and exhibit similar behavior as described previously [262]. In fact, these cells have the ability to manipulate the TME through the modulation of the ECM’s structure. This modulation promotes the interaction between BC cells and the stromal cells, possibly boosting cancer growth and chemoresistance while also producing significant immunosuppressive effects [263]. Mechanistically, CAF cells promote the development, differentiation, and mobility of M2-polarized macrophages, T-reg cells, and MDSCs [262, 264]. They can suppress NK cells by downregulating various NK cell activating receptors, including the poliovirus receptor (CD155), NCR2/NKp44, NCR3/NKp30, and DNAM-1 (CD226) (Fig. 9) [265]. In addition, they augment the stromal mass of BC by secreting fibrillar and fibronectins; these establish a sturdy physical obstacle that hinders the infiltration of immune effector cells, affects their distribution in the TME, and facilitates intercellular communication (Fig. 9) [263]. Likewise, CAFs can suppress CD8^+^ T cells by inducing antigen-mediated activation-induced cell death (AICD) and FAS-mediated cell death in CD8^+^ T cells expressing high FAS. CAFs can hinder CD8^+^ T cells by expressing the inhibitory molecules PD-L1 and 2 [263], as well as up-regulating PD-1 and CTLA-4 on T-reg cells (Fig. 9) [264]. This interaction promotes BC cell immune evasion. Indeed, CAFs positively modulate the BC TME by secreting massive quantities of chemokines, pro-inflammatory cytokines, and growth factors. For example, CAFs increase the expression of various chemokines (MCP-1/CCL-2, CCL-5, CCL-17, CXCL-1, CXCL-2, CXCL-8, CXCL-12, CXCL-13, and CXCL-14). Also, the produce various cytokines (TNF-α, IL-1β, IL-4, IL-6, IL-8, and IL-10), in addition to various exosomes containing miRNA, various MMPs (MMP-1, MMP-7, MMP-9, MMP-11, MMP-12, and MMP-14), various growth factors (colony stimulating factor 1, 2 ((CSF-1,2), connective-tissue growth factor (c-TGF), platelet-derived growth factor (pDGF), IGF-1, FGF, HGF, TGF-1β, epidermal growth factor (EGF) and VEGF). CAFs utilize multiple signaling pathways (SMAD, JAK/STAT, RAS/RAF/MEK/ERK, PI3K/Akt/ERK, PI3K/Akt/mTOR, Notch, p38/MAPK/MMP-2/-9, Hippo, Hedgehog, and Wnt/β-catenin). CAFs also supporting their metabolism and BC metabolism by several metabolic proteins (STAT-1, STAT-3, HIF-1α, GLUT-1, alpha-ketoglutarate (α-KG), COX-2, and PKM2), and other oncogenic proteins such as PGE_2_, TGF-1β, FOS-like 2 (FOSL-2), and clusterin. All these materials can promote BCSCs growth, in addition to drug resistance, development, EMT, migration, angiogenesis, and invasion of BC cells, as well as immunosuppressive cells recruitment to the BC TME (Fig. 9) [262–265]. Based on the data presented, it can be inferred that the interaction between TNF-α and TNFR2 has the potential to significantly influence many different signaling pathways and the oncogenic proteins involved in tumorigenesis. In fact, it was shown that activation of the JAK/STAT3 signaling pathway, through TNFR2 stimulation, supported the phenotypic stability of CAFs and improved their function [263]. Similarly, the secretion of CXCL-12 from BC cells, mediated by TNFR2 activation, can greatly amplify the pro-tumorigenic activities of CXCL-4^+^CAFs in the TME [140]. In addition, CAFs have the ability to release CXCL-12, which promotes the attraction of a variety of immunosuppressive cells expressing CXCR4, such as T-regs, MDSCs, EPCs, B-regs, and M2 macrophages. Indeed, α-SMA^+^CAFs have been shown to promote the proliferation of CD44^+^CD24^‾^BCSCs through the signaling pathway axis involving CXCR4 and its ligand, CXCL-12 [266]. This suggests that the TNFR2/CXCR4/CXCL-12 cascade may have a significant impact on the recruitment of immunosuppressive cells, the initiation of cancer, and the proliferation of tumor cells, thereby supporting the development of BC.

TNF-α also induced the expression of IL-33 in CAFs via the TNFR2/NF-κB/interferon regulatory factor 1 (IFR-1) pathway [267]. This process involves the binding of NF-κB-p65 to the IFR-1 sequence in the IL-33 promotor region, upon TNF-α/TNFR2 interaction. This binding event subsequently leads to the up-regulation of IL-33. The evidence supporting TNFR2 as the regulator of IL-33 expression, as opposed to TNFR1, is derived from the observation that IL-33 expression was eliminated by anti-TNFR2 antibodies but not by anti-TNFR1 antibodies [267]. The interaction between IL-33 and its receptor, ST2, is well-established in BC and has been linked to both onset and malignant transformation [268]. Additionally, IL-33 facilitates EMT, tumor invasion, and metastasis by binding to ST2 on BC cells. This triggers the activation of various signaling pathways including TNFR2, ERK/2-SP1-ZEB2, Akt, GLUT-1, p38, mTOR, JNK, and NF-κB, as well as other oncogenic pathways such as TNF-α, MMPs (2 and 9), PGE_2_, CCL-2, and CCL-5 [268]. IL-33-expressing CAFs can also indirectly cause immunosuppression (Fig. 6) [268]. IL-33 promotes the immunosuppressive functions, as well as the recruitment and activation, of MDSCs through the induction of ARG-1, NF-κB, and the MAPK signaling pathway (Fig. 9). The ST2/IL-33 axis is also essential for the recruitment and activation of T-regs, tumor-associated macrophages (TAMs), group 2 innate lymphoid cells (ILC2s), and mast cells. Infiltration of these cells into the TME ultimately leads to high immunosuppression [268] and facilitates tumor immune escape. It should also be noted that TNF-α may be secreted by both BC cells and CAFs or other immune cells in the TME, leading to constitutive TNFR2 activation. However, further studies are necessary to clarify whether the activation of TNFR2 is facilitated by s-TNF-α or Mb-TNF-α, as both may produce distinct outcomes.

In addition to IL-33, progranulin (PGRN), a secreted glycoprotein containing 7.5 cysteine-rich repeat motifs, has been discovered to be essential for the development of certain malignancies, including BC [269]. It was found to stimulate TAM polarization by effectively augmenting PD-L1, STAT3, ARG-1, CD206, α-SMA, and fibroblast activating protein alpha (α-FAP) expression while simultaneously reducing iNOS and CD86 production, thus promoting CD8^+^ T cell exclusion (Fig. 9) [269]. The FAP^+^CAFs were discovered to utilize the CXCR4/CXCL-12 axis to effectively exclude CD8^+^ T cells from the region containing cancer cells by releasing CXCL-12, ultimately preventing the CD8^+^ T cells from reaching the TME [270]. In addition, the secretion of IL-6 by CAFs can result in excessive activation of STAT3. This activation will prevent the development of M1 macrophages and cause monocytes to become immature and suppressive, resulting in reduced numbers of functional M1 macrophages in the BC TME [271]. Mechanistically, CAFs were found by utilizing RIG-1 receptor which receive signals from CAF-derived exosomes to stimulate STAT1 in BC leading to Notch-3-mediated BCSC drug resistance. CAFs can also take advantage of the BC TME’s hypoxic conditions to deliver circ-HIF-1α, mediating miRNA-580-5p activation and modulating CD44 expression [272]. This suggests that the TNFR2-mediated activation of HIF-1α, STAT1, and Notch-3 pathways could have a significant impact on this particular process. Hypoxia can induce metabolic reprogramming of CAFs, promoting a pro-glycolytic phenotype through promoter hypomethylation of certain genes including, HIF-1α, PKM2, HK2, lactate dehydrogenase-alpha (LDHα), and GLUT1 (Fig. 9) [273]. CAFs transfer these metabolites to BCs to support their metabolism and growth [273]. These data indicate a connection between TNFR2/HIF-1α and CAF metabolism; however, this notion requires more investigation. Remarkably, tumor-derived PGRN can prompt fibroblast differentiation into CAF by activating TNFR2, which subsequently triggers the up-regulation of VEGF and the Akt/ERK signaling pathway [274]. Moreover, it appears that TNFR2 can be activated in the absence of TNF-α via PGRN, adding a second mechanism by which TNFR2 can be activated absent its ligand.

#### The effect of TNFR2-mediated immunosuppressive cell activation on the antitumor function of dendritic cells

Dendritic cells (DCs) are a type of immune cells that facilitate the activation of the adaptive T cell immune response by antigen processing and presentation. Therefore, they are referred to as professional APCs. They are categorized based on their anatomical location, developmental history, observable characteristics, and function [275]. On the basis of their phenotypic features, these cells are typically categorized as plasmacytoid pre-DCs (p-DCs), type 1 conventional DCs (c-DC1s), and type 2 conventional DCs (c-DC2s). c-DC1s rely on the Bcl-2, interferon regulatory factor family 8 (IRF8), inhibitor of DNA binding 2 (ID2), nuclear factor interleukin-3 (NFIL3), zinc-finger and BTB domain-containing-46 (ZBTB46), and basic leucine zipper transcription factor ATF-like 3 (BATF3) transcription factors for their differentiation a maturation [260]. c-DC2s, on the other hand, rely on the IRF4, PU.1, zinc-finger E-box-binding homeobox-2 (ZEB-2), RelB, NOTCH, and recombination signal binding protein-Jκ (RBP-J) transcription factors for their differentiation and maturation, among others [275].

c-DC1s are known to have a central function in the TME by leveraging their ability to cross-present (cross-priming) tumor-associated antigens (TAAs), eliciting a robust CD4^+^ and CD8^+^ T cell-mediated anti-tumor immune responses involving both MHC-I and II [276]. Specifically, c-DC1s stimulate CD8^+^ T cell and CD4^+^ Type 1 Helper T cell (Th1) maturation and differentiation via cell-to-cell interaction (CD40/CD40L). This causes the T cells to secrete massive quantities of IL-12 and IFN-γ, resulting in significant anti-tumor immune responses [277]. This interaction between CD40 and CD40L also has the potential to activate and enhance c-DC1s function in return, indicating the possibility of mutual activation between these cells. After activation, c-DC1s exhibit the expression of other co-stimulatory molecules such as CD80 and CD86, which are indicative of complete maturation. CD80 and CD86 on c-DC1s have the ability to either stimulate or hinder T cells upon interaction with CD28 or CTLA-4, respectively. Moreover, c-DC1s can suppress CD8^+^ T cells by means of the interaction between PD-L1 and PD-L2 on c-DC1s and PD-1 and V-domain immunoglobulin suppressor of T cell activation (VISTA) on CD8^+^ T cells [224]. It is also important to note that c-DC1s-mediated TAA presentation without co-stimulatory signals can lead to T cell anergy, thereby limiting their activity.

Another important aspect of c-DC1s’ ability to shape the TME is their use of various chemokines and chemokine receptors. c-DC1s exhibit increased expression of CCR-5 and CXCR4, in addition to constitutive expression of CCR-7, the c-type lectin receptor CD370/DNGR-1/CLEC9A, and X-C motif chemokine receptor 1 (XCR1). As a result, they are likely to be mobilized towards the TME or TDLNs in response to CCL-4, CXCL-12, and CCL-19/CCL-21 chemokines, respectively (Fig. 9) [224, 276]. It should be noted that the cells of the BC TME are the primary sources of these chemokines. Similarly, c-DC1s have the capability to secrete CXCL-9 and CXCL-10, thereby facilitating the recruitment of CD8^+^ T cells to the TME [224]. As such, the c-DC1s are crucial for promoting a strong anti-tumor immune response in the TME; this elucidates the significance of c-DC1s, being the sole subset of myeloid cells capable of transporting TAAs from the TME to the TDLNs and activating naïve CD8^+^ T cells through cross-presentation [278]. However, the immunosuppressive gradient (ISG) in BC TME, which is partly caused by TNFR2 activation on TME cells, can lead to dysfunction, altered phenotype, poor differentiation, poor circulation, and improper tolerance in c-DC1s, ultimately limiting or excluding their presence in the TME. Notably, in addition to the immunosuppressive soluble mediators, c-DC1s can also be blocked by cell-to-cell contact, thus inhibiting the effector functions of c-DC1s as well as the CD8^+^ T cell anti-tumor immune response. These observations may provide a potential explanation for the significantly diminished quantities of c-DC1s within the TME of BC [279, 280]. The ISG in the TME of BC also employs various other mechanisms to impair the functions of c-DCs1. In fact, HIF-1α, COX-2/PGE_2_, VEGF/VEGFR, JNK, STAT3, TGF-1β, SMAD, Wnt/β-catenin, IL-1β, IL-4, IL-6, IL-10, and G-CSF all contribute to c-DC1 impairment. HIF-1α, in particular, can activate and release VEGF from BC cells, which can then interact with VEGFR on c-DC1s (Fig. 9). This interaction will induce *PD-1* up-regulation and significantly stimulate cofilin 1 (COF-1) over-expression, resulting in CD8^+^ T cell exhaustion and targeted destruction of filamentary actin (F-actin), respectively [242]. This process subsequently leads to impaired differentiation and migration of c-DC1s. In addition, VEGF has the ability to effectively suppress the activity of FMS-like tyrosine kinase 3 ligand (FLT3L), a central transcription factor in the growth and development of c-DC1s. Consequently, this inhibition negatively affects the differentiation and survival of c-DC1s [224]. Similarly to VEGF, HIF-1α can up-regulate the *COX-2* gene. It does this by binding to a specific sequence in the promotor of *COX-*2. This leads to PGE_2_ secretion and subsequently increases the expression of *PD-L1*, inhibiting the maturation, differentiation, and survival of c-DC1s (Fig. 9) [224, 242]. In fact, PGE_2_ promotes a change in the cytokine profile of c-DC1 cells, leading to a shift from Th1 polarization to Th2 polarization in the TME [281]. HIF-1α also binds to the promoter regions of IL-10 and TGF-1β, leading to the activation of the JAK/STAT and TGF-1β/SMAD signaling pathways [242]. This ultimately prevents c-DC1s from effectively priming CD8^+^ T cells and Th1 cells. Furthermore, the over-expression of IL-6 and IL-10 induced by BC and other immunosuppressive cells will result in the activation of STAT3. This activation, in turn, will suppress the production of IL-12 and IFN-γ by c-DC1s and impede their differentiation (Fig. 9) [224]. Mechanistically, BC TME-induced G-CSF was observed to hinder the development, differentiation, and maturation of c-DC1s [280]. G-CSF suppresses IRF8 by activating STAT3, leading to a decrease in both its mRNA and protein levels. G-CSF was seen to decrease the levels of MHC molecules, *TapBP*, *Tap-2*, *Batf-3*, and *Pml* mRNA. However, there was no change in the level of *Id2* mRNA [280]. The restoration of IRF8 function and normal development of c-DC1s was successfully achieved through the neutralization of G-CSF and the deletion of its receptor, *Csf3,* using CRISPR**-**Cas9 technology [280]. Additionally, excessive activation of the Wnt/β-catenin pathway in BC cells can also inhibit the migration and recruitment of c-DCs [277]. The down-regulation of CCL-4 by Wnt/β-catenin results in reduced tumor infiltration by c-DC1. BC cells, in addition to other immunosuppressive cells secrete PGE_2_ which can reduce the presence of tumor-infiltrating NK cells and inhibit NK cells production of XCL1 and CCL-5. This reduction limits the presence of c-DC1s in the TME (Fig. 6) [224]. Moreover, HIF-1α can efficiently inhibit NK cells, which in turn inhibits NK cell-mediated c-DC1 activation in the BC TME.

As stated previously, TNFR2 has been found to promote the growth and progression of BC cells, resulting in increased BC cell mass and reduced blood vessel formation. Consequently, this could act as a physical barrier, effectively hindering the infiltration and recruitment of c-DC1 cells into the TME. In fact, T-regs were found to up-regulate certain chemokine receptors, such as CXCR-3, CCR-4, and CCR-8, in the BC TME [282]. In particular, CXCR-3 is required for T-reg recruitment and immunosuppressive activation. CXCR-3-expressing T-regs were found to be in close proximity to XCR1^+^-CXCL-9^+^ c-DC1, with CXCL-9 being central to this process [282]. Once T-regs are recruited, they compete with CD8^+^ T cells for the CXCL-9 gradient, thus restricting the proper activation of CD8^+^ T cells by c-DC1 [282]. Once in the TME, T-regs can secrete IL-6, IL-10, and TGF-β, thus inhibiting both CD8^+^ T cells and c-DC1s. This insinuates a correlation between CXCR-3 and TNFR2 expression on T-regs (Fig. 9) [282], since TNFR2^+^T-regs is the most potent immunosuppressive phenotype. However, further investigation is needed to validate this notion. In summary, the activation of the TNFR2 signaling pathway seems to negatively influence the effective CD8^+^ T cells priming, movement, chemoattraction, survival, and stimulation of dendritic cells in the BC TME. Yet, the impact of TNFR2 on DCs in the TME, particularly in BC, remains incompletely understood. Therefore, there is an urgent requirement for further research to investigate this matter.

## Clinical significance of TNFR2 expression in BC

Although the expression of TNFR2 has been extensively studied in different cancer models, both in vitro and in vivo, the clinical relevance of this receptor in BC is still a topic of discussion and ongoing investigation. Multiple studies have suggested that sTNFR2 may play a significant role in the clinical pathophysiology of BC. This is in addition to the common idea that membrane-bound TNFR2 generates strong signals when it interacts with Mb-TNF-α, thereby activating various biological processes that support tumor growth. In fact, the effects of sTNFR2 in the blood varied between fatigue and worse prognosis. It was initially reported by Tesarová et al. that plasma sTNFR2 levels were greater in BC patients than in control patients, and this increase correlated with higher TNF-α levels [283]. TNF-α levels decreased considerably following the treatment, but sTNFR2 levels remained relatively unchanged [283]. Although no correlation was shown between the elevated levels of TNF-α/sTNFR2 and BC pathophysiology, a separate study involving 103 early-stage BC patients who had undergone surgery or received radiation and/or chemotherapy found that a majority (60%) experienced notable fatigue and sleep disturbances after treatment. The chemotherapy group exhibited elevated levels of TNF-α/sTNFR2, which were significantly correlated with fatigue and sleep disturbances. However, no significant association was observed between TNF-α/sTNFR2 and fatigue or sleep problems in the other groups [284]. Another study also found that levels of sTNFR2 were higher in the plasma of BC patients who received chemotherapy compared to those who did not receive chemotherapy. Elevated levels of sTNFR2 were linked to fatigue [285–287]. Subsequent studies have provided further support for this notion, indicating that elevated levels of sTNFR2 and leptin in post-menopausal women are each independently linked to an increased risk of BC [288]. Additionally, increased levels of sTNFR2 were also associated with the development of cancer-related cognitive impairment (CRCI)/chemotherapy-associated cognitive impairment (CACI) and memory loss in BC patients [289, 290]. It is important to note that these reports focused on BC survivors who successfully completed chemotherapy. A recent study examined the impact of sTNFR2-induced CRCI/CACI before and after chemotherapy in BC patients. The study observed a positive correlation between elevated sTNFR2 levels and cognitive impairment [291]. In fact, a study by Bulska-Będkowska et al*.* also found that sTNFR2 levels were strongly related to an increased risk of BC development in the later stages of the disease [292]. Surprisingly, doxorubicin combined with 2-mercaptoethane sulfonate (MESNA) significantly reduced the plasma levels of sTNFR2 post-therapy in BC patients compared to those only receiving doxorubicin [293]. This validates the potential benefit of targeting the TNF-α/sTNFR2 axis pre- and post-chemotherapy to improve the overall quality of life (QOL) in BC patients. Interestingly, a clinical study (NCT01478477) in early-stage BC survivors with chemotherapy history evaluated diet quality using the Healthy Eating Index-2010 (HEI-2010) to see how it correlated with inflammation, health, and/or functional outcomes that impacted QOL. The study discovered that a healthier diet, as indicated by a higher overall HEI-2010 score, was linked to lower plasma levels of sTNFR2. This reduction in sTNFR2 was associated with improved health status and functional capacity [294], indicating that a healthy diet may possess anti-inflammatory properties, especially against sTNFR2. Connor et al. investigated the association between increased levels of sTNFR2 and the mortality risk among BC survivors of Hispanic and non-Hispanic backgrounds. The study found a significant association between high levels of sTNFR2 in the blood and an increased risk of mortality, specifically in obese Hispanic women [295]. This implies that obesity could be a contributing factor to elevated plasma levels of sTNFR2. This also suggests that effective lifestyle management may decrease mortality risk in women by reducing obesity-related sTNFR2 levels. The study did not investigate the specific anti-cancer drugs employed, which could contribute as an additional explanation for the increased levels of sTNFR2 in the plasma, since different drugs, such as chemotherapy and radiotherapy, may increase the concentration of this receptor [294]. However, a study of 142 women diagnosed with primary BC found no association between plasma levels of sTNFR2 and the risk of developing BC [296]. Despite the fact that multiple experimental studies have demonstrated that sTNFR2 can reduce inflammation by effectively competing with transmembrane TNFR2 in terms of their binding ability to TNF-α [167], the notion that sTNFR2 causes inflammation and other cancer-related problems in BC patients must be revised to account for this fact when designing clinical trials in the feature. Furthermore, the exact mechanism by which sTNFR2 triggers inflammation, whether by binding to TNF-α or via other pathways, as well as which cell types in the BC TME are the source of sTNFR2, are yet to be elucidated in BC. Moreover, a limited number of studies have found a link between membrane-bound TNFR2 and the clinical manifestations of BC. The initial identification of an association between TNFR2 and its prognostic significance in BC patients was made through a molecular investigation. A biallelic polymorphism, specifically located in exon 6 of TNFR2, results in variations by substituting thiamine (T) with guanine (G) in codon 196. This substitution produces the abnormal allele 196 M/R [297]. Indeed, the 196 M/R allele was found to be associated with an increased risk of BC, especially in post-menopausal patients. This allele was also linked to lower overall survival (OS) and decreased disease-free survival (DFS). On the other hand, the presence of the 196R/R allele was actually associated with a more positive response [297]. These findings indicate that 196 M/R has the potential to serve as a biomarker for both the severity and late-onset occurrence of BC in post-menopausal women. Supporting this notion, in a study including 125 patients with primary BC, a significant association was shown between TNFR2 and worse outcomes, such as increased tumor size, a more advanced clinical stage, a higher pathological grade, reduced OS, and DFS [298]. Comprehensive molecular investigations in BC patients are necessary to examine if there are other polymorphisms in the TNF-α and TNFR2 genes that could potentially enhance their interaction and whether these polymorphisms are BC-subtype specific. Paradoxically, several clinical studies have found a correlation between TNF-α or NF-κB and a negative prognosis in patients with BC [299–301]. Nevertheless, it is important to note that there is currently no confirmed correlation between TNF-α, NF-κB, and sTNFR2. This highlights the necessity for further investigation to fully understand and incorporate this concept. Similarly, the expression of TNFR2 was observed to be elevated in both in situ and infiltrating carcinomas when compared to benign BC. Infiltrating carcinomas exhibit notably higher expression levels compared to in situ carcinomas. Additionally, there is evidence suggesting that the expression of TNFR2 is directly linked to the production of IL-6, potentially through the involvement of the NF-κB pathway [302]. Furthermore, the expression of TNF-α and TNFR2 was observed in uncommon subtypes of BC, specifically invasive micropapillary carcinoma (IMPC), which is known for its high incidence of lymph node metastasis and unfavorable prognosis. IMPC exhibited higher expression of TNFR2 compared to invasive ductal carcinoma (IDC) [303]. The study revealed positive associations between TNF-α expression and the rates of cellular proliferation, lymph node metastasis, microvessel density, and histological grade. Additionally, TNFR2 expression was found to be associated with the rate of cellular proliferation and TNF-α expression [303]. Overall, while previous studies have attempted to provide a clear understanding of TNFR2 expression in human BC, there is a lack of clinical trials that clarify the precise role and potential mechanisms of TNF-α/TNFR2 in human BC TME. Massive and rigorously designed clinical studies that encompass different races are urgently required to find the association between TNF-α/TNFR2 and BC development. Studies are also required to determine the association between TNF-α/TNFR2 and different BC subtypes, as well as whether the degree of this association affects the aggressiveness of these subtypes in terms of their metastasis, recurrence, and drug resistance to provide an effective targeted therapy in the near future.

## Targeting TNFR2 in BC TME

### Anti-TNFR2 antagonistic antibodies

The main goal of a successful targeted treatment for TNFR2, aimed at enhancing strong anti-tumor effects, is to impede the complex signaling pathway of the receptor. This can be achieved through direct interaction with the receptor or by inhibiting Mb-TNF-α and s-TNF-α. However, the complexity of the TNFR2 signaling pathway can result in the activation of various downstream signaling pathways, which may ultimately contribute to the potential failure of any treatment. Despite this challenge, several researchers have attempted to selectively target TNFR2 in various tumor types, including BC [8, 175, 304–308], to enhance anti-cancer immune response. One drug in particular, cyclophosphamide, has been used to deplete CD4^+^CD25^+^TNFR2^+^T-regs in a murine model of malignant mesothelioma, thereby allowing the activation of CD8^+^ T cells. The observed depletion of CD4^+^CD25^+^TNFR2^+^T-regs is hypothesized to be a result of the downregulation of TNFR2 expression on the surface of these cells [309]. Torrey et al. discovered that two antibodies that antagonize TNFR2 effectively suppresses ovarian cancer cells (OVCAR3) and the growth of T-reg cells, even with TNF-α present [310]. This was achieved by inhibiting the activation of various proteins involved in the TNFR2 signaling pathway, including RelB, TRAF2, TRAF3, cIAP2/BIRC3, MAP3K11, CHUK, NFKBIA, and NFKBIE, resulting in inhibition of RelA/NF-κB phosphorylation [311]. This led to the expansion and activation of T-eff cells and CD8^+^ T cells. Interestingly, the inhibitory effect of TNFR2 antagonistic antibodies extended to s-TNFR2, even though the presence of soluble receptors could impede the action of these antibodies by sequestering them, thereby limiting their interaction with TNFR2 [310]. Moreover, in a mouse model of colon cancer, the M861 anti-TNFR2 antibody was found to decrease trans-membrane TNFR2 levels and effectively inhibit the expansion and proliferation of T-regs. M861 exhibited an indirect effect on T-regs by inhibiting the interaction between TNF-α and TNFR2 [196]. However, despite the fact that CT26 cells are known to express TNFR2, M861 was found to be ineffective in inhibiting CT26 tumor activity [196]. This suggests that M861 specifically targets TNFR2^+^ T-reg cells. In particular, the combination of M861 and the toll-like receptor 9 ligand CpG oligodeoxynucleotide (CpG-ODN) resulted in a significant reduction in surface TNFR2 levels, a decrease in T-reg cell count, and an increase in CD8^+^ T cell count. Additionally, this combination enhanced the capacity of CD8^+^ T cells to generate and release IFN-γ [196]. CpG-ODN may activate and improve the activity of other immune cells, including DCs, B cells, and CTLs, while also diminishing the immunosuppressive function of MDSCs [196]. Alternatively, CpG-ODN-mediated DC and B cell activation may also lead to the production of substantial amounts of TNF-α, leading to T-reg activation and expansion through TNFR2. Similarly, SB203580, a selective inhibitor of p38 MAPK, was found to substantially inhibit TNFR2^+^ T-reg function and proliferation in vitro and in vivo, whereas sulfasalazine and Bay-11–7082 (NF-κB inhibitors) failed to suppress TNFR2^+^ T-regs [312]. This indicates that SB203580 could target and inhibit other TNFR2-activated downstream singling pathways, such as p38-MAPK, while sulfasalazine and Bay-11–7082 could not. SB203580 specifically inhibited the expression of TNFR2 and FoxP3. SB203580 also activates DCs to stimulate anti-tumor immunity by activating T-eff cells through the inhibition of TNFR2^+^T-regs [312].

Previous studies have aimed to identify effective anti-TNFR2 antibodies that can sufficiently block the interaction between TNFR2 and TNF-α. This inhibition could potentially limit cancer progression and the immunosuppressive effects of T-regs and other TNFR2-expressing immunosuppressive cells. However, the precise mechanism of action of these anti-TNFR2 antibodies remains unclear. Tam et al. [313] conducted a study to elucidate the underlying mechanism through which anti-TNFR2 antibodies exert their effects on TNFR2. They discovered five anti-TNFR2 antibodies, H5L10, Y7, Y9, Y10, and M3, selectively bound to certain epitope structures in the 4 CRDs. CRD4 is bound by H5L10, CRD2 by Y7, CRD1 by Y9, CRD3 by Y10, CRD1 and CRD2 by M3. Y9 showed the greatest affinity binding (0.2 nM) to TNFR2, followed by Y10 (0.5 nM), H5L10 (1.0 nM), M3 (2.8 nM), and Y7 with a binning affinity of 25 nM [313]. Because Y7 and Y10 possess the capacity to bind epitopes in CRD2 and CRD3, respectively, this enabled them to overlap with the ligand interface, thereby effectively blocking TNF-α. Similarly, Y9, despite its interaction with the outside of the ligand interface, had the highest efficacy in inhibiting TNF-α when compared to other antibodies targeting TNFR2 [313]. More specifically, Y9 antibodies activated CD8^+^ T cells via NF-κB activation, resulting in enhanced expansion, survival, and cytokine production. Additionally, Y9 antibodies up-regulated the expression of CD25, PD-1, and granzyme B. Likewise, Y9 stimulated the activation of NK cells, T-regs, and conventional CD4^+^ T cells. The proposed mechanism of action for Y9 is believed to include powerful Fc-dependent co-stimulation, facilitating higher-order cross-linking of TNFR2 and subsequently acting as an agonist [313]. It is important to note that Y9 does not promote TNFR2-mediated T-reg depletion, and this was supported by the fact that treatment with Y9 antibodies in vitro did not affect the immunosuppressive activity of T-regs but rather activated them [313]. This suggests that Y9 behaves as a cross-linkage activator agonist for transmembrane TNFR2 and does not compete with TNF-α. Importantly, Y9 antibodies exhibited comparable outcomes in an in vivo model of CT26 syngeneic tumors [313]. In fact, Y9 displayed a superior anti-tumor immune response when paired with the murine anti-PD-1 (J43) in the CT26 and EMT6 mice models. Interestingly, in a WEHI-164 mouse model, J43 or Y9 alone had a comparable anti-cancer impact, but their combination had no statistically significant effect [313]. In all three models, the combination of Y9 with murine anti-PD-L1 (MPDL3280a) demonstrated improved anti-cancer effects. Suggesting a mechanism by which Y9 functions as a co-stimulator, activating effector immune cells such as NK cells, CD8^+^ T cells, and APCs, while J43 and MPDL3280a provide protection to immune cells against cell-to-cell contact inhibition caused by cancer cells, T-regs, and MDSCs. This also indicates that anti-TNFR2 antibodies such as Y9 could be used in combination treatment strategies with other immune checkpoint inhibitors (ICIs). Furthermore, Y9 exhibited acceptable toxicity levels in comparison to anti-CTLA-4 antibodies [313].

Contrary to the previous notion of anti-TNFR2 antibody-mediated CD8^+^ T cell co-stimulation, upcoming studies suggest that the anti-TNFR2 antibody activity is due to the blocking of TNFR2 to prevent TNF-α binding on both cancer cells and immune suppressive cells. Recently, Yang et al. designed human chimeric anti-TNFR2 IgG2 antibodies and found that they effectively eliminated both cancer cells and T-reg cells while leaving T-eff cells unaffected, suggesting a TME-directed killing specificity [314]. Following the same notion, the combination of anti-TNFR2 (TY101) antibodies and anti-PD-1 (CD279) antibodies effectively reduced the number of TNFR2^+^T-regs and increased the ratio of CD8^+^ T cells in murine cancer models of CT26 and MC38 [304]. This combination led to complete tumor remission and eradication, surpassing the effects of using anti-TNFR2 or anti-PD-1 alone. However, in vitro experiments revealed that TY101 exhibited a lower capacity to induce cell death in CT26 and MC38 cells [304]. In addition, TNFR2 antibodies were provided with different Toll-like receptor (TLR) agonists to function as anti-tumor medications. Regarding this matter, the combination of TY101 and CD120b antibodies with high mobility group nucleosome binding proteins 1 (HMGN-1, TLR-4 agonist) and R848 or 3 M-052 (TLR-7/8 agonists) showed a highly effective immune response against tumors [306, 307]. The antibodies effectively targeted T-regs and tumor cells, resulting in a significant reduction in tumor growth. TLR agonists were found to activate DCs, leading to the production of IL-12 and TNF-α. Additionally, they were observed to stimulate CD8^+^ T cells [306, 307], indicating a potential synergistic effect when combined with TNFR2 antibodies. Similarly, scutellarin, a flavonoid compound, was found to effectively inhibit TNFR2 on both tumor and T-reg cells. Scutellarin prevented the binding of TNF-α to TNFR2 by forming hydrogen bonds, arene-arene, and hydrophobic interactions with specific amino acid residues (Arg77, Arg108, and Arg113) in region 4 of TNFR2 [308]. This leads to the inhibition of p-38 MAPK phosphorylation. When combined with CpG-ODN-treatment, it enhances the ratio of CD8^+^ T cells [308], thereby promoting a robust anti-tumor immune response. Likewise, the application of TNFR2 inhibition was tested in the pancreatic ductal adenocarcinoma (PDAC) and colon cancer mouse models using anti-PDL-1 and anti-PD-1 antibodies, respectively, and the findings were consistent with previous reports [175, 315]. Indeed, combining anti-TNFR2 with anti-PD-1 or anti-PD-L1 antibodies seems to generate a potent anti-tumor immune response in BC. When Ab-1 and Ab-2 anti-TNFR2 antibodies were combined with nivolumab (anti-PD-1) in a mouse model of MDA-MB-231-established BC, there was a notable increase in CD4^+^ T cells and CD8^+^ T cells [313]. This combination also resulted in a stronger anti-tumor immune response compared to using anti-PD-1 alone. The binding affinity of Ab-1 and Ab-2 to the human CRD1 region is 700 and 200 pM, respectively. These antibodies do not overlap with each other and effectively block the binding of TNF to TNFR2, with IC_50_ values of 177 pM for Ab-1 and 89 pM for Ab-2 [313]. Supporting this notion, anti-TNFR2 antibodies induced apoptosis, inhibited the proliferation of 4T1 breast cells in vitro, and reduced tumor growth in vivo [8]. Moreover, anti-TNFR2 antibodies inhibited CD4^+^CD25^+^TNFR2^+^T-regs proliferation and reduced FoxP3 expression, thus ablating their inhibitory effect and increasing the proportion of CD8^+^ T cells [8]. When combined with anti-PD-L1 antibodies, they demonstrated a synergistic anti-tumor immune response compared to anti-PD-L1 antibodies alone. Similarly, the combination therapy affected the cytokine profile by increasing the expression of IL-17A, CXCL-10, and IFN-γ as well as reducing the expression of TGF-1β, IL-10, TNF-α, and TNFR2 [8]. This, in turn, could enhance the recruitment of CD8^+^ T cells to the BC TME. Collectively, these reports highlight the importance of targeting TNFR2 in order to ensure a strong anti-cancer immune response. They also indicate that anti-TNFR2 antibodies are effective both alone and when paired with ICIs. This further suggests that these antibodies could be used for the treatment of patients who do not respond or cannot tolerate anti-PD-L1 or anti-PD-1 antibodies. Inhibiting both tumor- and T-reg-expressing TNFR2 seems to be a valid approach in modulating the immune response in favor of cancer eradication by enhancing effective CD8^+^ T cell activation by DCs. Indeed, inhibiting the binding of TNFR2 and its ligand TNF-α through high-affinity antibodies, especially in the CRD regions where both proteins interact the most, is crucial to eliminating the burden of TNFR2 as an immunosuppressive protein in the BC TME. However, functional studies regarding the effective doses and their associated toxicity are needed to move on to the next stage of human cancer patient application. Table 1 summarizes different anti-TNFR2 agents and their mechanism of action.

**Table 1:** Different TNFR2 targeted therapies and their mechanisms of action

Targeted therapy	Molecular target (TNFR2 or downstream)	Mechanism of action	Cancer type (Tumor model)	Status (Animal or Clinical)	Ref
Cyclophosphamide	CD4^+^CD25^+^TNFR2^+^T-regs	Activate CD8^+^ T cells	Mesothelioma	mouse model	[309]
2 TNFR2 antagonistic antibodies	RelB, TRAF2, TRAF3, cIAP2/BIRC3, MAP3K11, CHUK, NFKBIA, and NFKBIE	Inhibit Treg proliferation, reduce soluble TNFR2 secretion from normal cells, and enable T effector cell expansion. Inhibit RelA/NF-κB phosphorylation	Ovarian cancer	Cells (ovcar3)	[311]
M861 anti-TNFR2 antibody	TNFR2^+^ T-reg cells	Inhibit interaction between TNF-α and TNFR2 Decrease trans-membrane TNFR2 levels and inhibit the expansion and proliferation of T-regs	Colon cancer	Mouse model	[196]
M861 and the toll-like receptor 9 ligand CpG oligodeoxynucleotide (CpG-ODN)	TNFR2^+^ T-reg cells	Reduce surface TNFR2 levels Decrease T-reg cell count Increase CD8^+^ T cell count, release IFN-γ	Colon cancer	Mouse model	[196]
CpG-ODN	DCs, B cells, and CTLs	Diminish immunosuppressive function of MDSCs Produce substantial amounts of TNF-α, activate and expand T-regs through TNFR2	Colon cancer	Mouse model	[196]
Y7, Y9 and Y10	Bind epitopes in CRD1 to CRD4	Overlap with the ligand interface to effectively block TNF-α	Colon cancer, breast cancer, fibrosarcoma, B lymphocyte sarcoma, melanoma	Mouse model	[313]
TNFR2 IgG2 antibodies	T-reg cells	TME-directed killing specificity	T cell lymphoma	Patients and cell lines	[314]
anti-TNFR2 (TY101) antibodies and anti-PD-1 (CD279)	TNFR2^+^T-regs CD8^+^ T cells	Reduce the number of TNFR2^+^T-regs Increase the ratio of CD8^+^ T cells	Colon cancer	Murine colon cancer models of ct26 and mc38	[304]
TY101, CD120b	nucleosome binding proteins 1 (HMGN-1, TLR-4 agonist) and R848 or 3 M-052 (TLR-7/8 agonists)	Deplete Tregs and stimulate cytotoxic CD8 T cell activation	Colon cancer	Murine colon cancer	[306, 307]
scutellarin	TNFR2 on both tumor and T-reg cells, prevent the binding of TNF-α to TNFR2	Inhibit p-38 MAPK phosphorylation	Colon cancer	Mouse ct26 colon cancer model	[308]
anti-PDL-1 and anti-PD-1	NF-κB-p65pathway	Inhibit growth, relieve tumor immunosuppression, and generate robust memory recall	Pancreatic ductal adenocarcinoma (PDAC) and colon cancer	Mouse models	[175, 304]
Ab-1 and Ab-2 anti-TNFR2 antibodies combined with nivolumab (anti-PD-1)	CRD1	Inhibit CD4^+^ T cells and CD8^+^ T cells	Breast cancer	Mouse model of MDA-MB-231-established BC	[313]
anti-TNFR2	IL-17A, CXCL-10, IFN-γ, TGF-1β, IL-10, TNF-α, and TNFR2	Inhibit CD4^+^CD25^+^TNFR2^+^T-regs proliferation Reduce FoxP3 expression Reduce inhibitory effect and increase the proportion of CD8^+^ T cells	Breast cancer	Mice model	[8]

T-regs, T-regulatory cells; TRAF2, TNF receptor associated factor 2; TRAF3, TNF receptor associated factor 3; cIAP2, cellular inhibitor of apoptosis 2; BIRC3, baculoviral IAP repeat containing 3; MAP3K11, mitogen-activated protein kinase kinase kinase 11; TGF-1β, transforming growth factor beta; IL-10, interleukin 10; IL-17A, interleukin 17 alpha; CXCL-10, C-X-C motif chemokine ligand 10; IFN-γ, interferon gamma; CRD, cysteine-rich domain; DC, dendritic cell; CTLs, cytotoxicity T lymphocyte; TNF-α; tumor necrosis alpha; TNFR2, tumor necrosis receptor type two; TME, tumor microenvironment; PD-1, programmed death 1; PD-L1, programmed death ligand 1

### New strategies to handle the deleterious effect of TNFR2 in BC TME

#### Bispecific antibodies

**Fig. 10:**
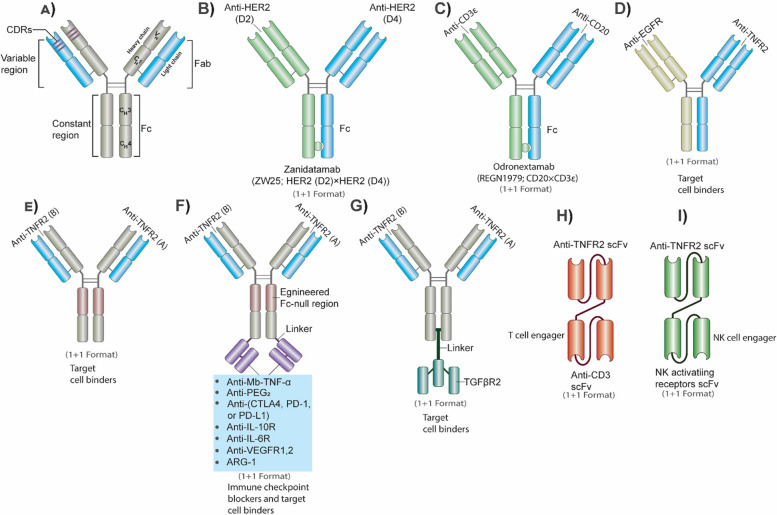
Bs-Abs for neutralizing TNFR2. a. The standard structure of an IgG antibody. b. Established bs-Abs, zanidatamab (ZW25; HER2 (D2) × HER2 (D4)) targeting both forms of HER2 (D2 and D4)) with format (1 + 1). c. Established bs-Abs, odronextamab (REGN1979; CD20 × CD3ε) targeting bs-Ab for both CD20 and CD3ε with format (1 + 1). While d, e, f, and g represent proposed different bs-Abs for targeting TNFR2 on both BC cells and immunosuppressive cells, in addition to some immunosuppressive receptors (EGFR, CTLA-4, PD-1, PD-L1, Mb-TNF-α, TGFβR2, VEGFR1,2, IL-6R, and IL-10R) and mediators (ARG-1 and PGE_2_) with format (1 + 1), h and i bs-Abs engagers for CD8^+^ T cells (h) and for NK cells (i) that target TNFR2 on both BC cells and the immunosuppressive cells as well as targeting and stimulating immune activating receptors on NK cells such as NCR1, NCR2/NKp44, NCR3/NKp30, NCR1/NKp46, NKG2D, MICA/B, MICA, as well as on CD8^+^ T cells such as CD3ε. TNFR2, tumor necrosis factor receptor type two; Mb-TNF-α, membrane-bound tumor necrosis factor; Bs-Ab**,** bi-specific antibody; CDR, combativity determine region; HER2, epidermal growth factor receptor 2; IL-6R, interleukin 6 receptor; IL-10R, interleukin 10 receptor; PD-1, programmed cell death 1; PD-L1, programmed cell death ligand 1; CTLA-4, cytotoxic T lymphocyte antigen 4; TGF-βR2, transforming-growth factor β receptor 2; VEGFR 1,2, vascular endothelial growth factor receptor 1 and 2; PEG_2_, prostaglandin-E2; ARG-1, arginase-1; NCR1, natural cytotoxicity triggering receptor 1; NCR3, natural cytotoxicity triggering receptor 3; MICA and B, MHC class I chain-related polypeptide A and B; NKG2D, natural killer group 2-member D; NKp30, natural killer protein 30; NKp44, natural killer protein 44; NKp46, natural killer protein 46

Over the past decade, there has been considerable interest in bi-specific antibodies (bs-Abs) because of their distinct and adaptable mode of action. Bs-Abs offer novel mechanisms and therapeutic applications that conventional IgG-based antibodies cannot perform [316]. Typically, bs-Abs function by binding to two separate antigens or two separate epitopes of the same antigen [316]. Bs-Abs can serve as connectors between immune effector cells (CD8^+^ T cells, NK cells, and APCs) and cancer cells and/or target numerous cancer-important signaling pathway receptors [316–318]. These antibodies can also perform dual receptor co-stimulation, tumor-targeting co-stimulation, dual receptor inhibition, and ligand-receptor inhibition. Bs-Abs have been made to target various proteins such as CD3ε, CD16, CD19, CD 20, CD223 (LAG-3), CTLA-4, PD-1, PD-L1, epithelial cellular adhesion molecule (EpCAM), and B cell maturation antigen (BCMA) [316, 318]. There are several bs-Abs that have the ability to alter various aspects of cancer TME, including tumor proliferation, angiogenesis, metastasis, and immunosuppression by targeting signaling pathways such as HER1, HER2, HER3, EGFR, TGF-β and VEGF. They include zenocutuzumab (MCLA-123; HER2 × HER3) and lazalontamab (SI-BOO1; EGFR × HRE3) [318], Zanidatamab (ZW25; HER2 (D2) × HER2 (D4)), retlirafusp alfa (SHR-1701; anti-PD-L1 × TGFβR2), vudalimab (XmAb20717; PD-L1 × CTLA-4) targets PD-L1 and CTLA-4 [318], and Ivonesocimab (AK112; PD-1 × VEGF). For T cell-mediated cytotoxicity (T cell engagement), odronextamab (REGN1979; CD20 × CD3ε) stimulates both CD20 and CD3ε (Fig. 10) [318]. It should be mentioned that some of the most effective of these bs-Abs antibodies are in the advanced stages of clinical development.

Bs-Abs come in many forms such as those with the ability to bind two different antigens are represented by the format 1 + 1. A bs-Ab that can bind more than two different antigens can have the formats 1 + 2, 1 + 3, or 2 + 2, depending on the context. The valency, or the number of antigens that a bs-Ab can bind, is what determines its format [319]. Formats are further categorized based on the building blocks that make them up, such as bs-Ab with FcγR and those without FcγR [319]. Bs-Abs containing FcγR are capable of carrying out various antibody effector functions, including antibody-mediated phagocytosis, antibody-mediated complement activation, and antibody-dependent cell-mediated cytotoxicity (ADCC). However, bs-Abs lacking FcγR due to mutations in the Fc region, do not possess these functions. This means that FcγR-containing bs-Abs have the potential to engage innate immune cells, such as NK cells, and simultaneously block receptor tyrosine kinases like HER1, HER2, and HER3 [318]. When designing T cell engager bs-Abs, it is important to use bs-Abs without FcγR to prevent unnecessary immune cell activation, recruitment, and cytokine secretion [318].

Several studies in the field of autoimmune diseases and cancer have found promising results from the use of bs-Abs targeting TNF-α and other receptors. For instance, it has been shown by Christel et al*.* that bs-Ab (TNF-α × CEA) against carcinoembryonic (CEA) and TNF-α enhances radiotherapy both in vivo and in vitro of colon cancer, particularly in tumors that overexpress CEA [320]. Two separate reports also discovered that Valpha, and V5-3, bs-Abs for TNF-α and VEGF (TNF-α × VEGF), can effectively inhibit TNF-α and VEGF impact [321, 322]. A novel bs-Ab antibody (TNF-α × Ang-2) targeting TNF-α and Angiopoietin 2 (Ang-2) was also found to successfully bind and inhibit both TNF-α and Ang-2 [323]. Several other studies observed that bs-Abs effectively targeted and inhibited TNF-α and IL-17, resulting in a notable reduction in the inflammatory response [324–326]. Another study showed that a bs-Ab targeting TNF-α and IL-6 (TNF-α × IL-6)successfully inhibited CXCR-13 activity in both in vitro and in vivo models of rheumatoid arthritis [327]. Given the availability of these bs-Abs and the fact that they can successfully target various receptors and signaling pathways, similar strategies could be employed to develop bs-Abs that effectively target TNFR2, Mb-TNF-α, and their associated receptor signaling pathways in BC.

**Fig. 11:**
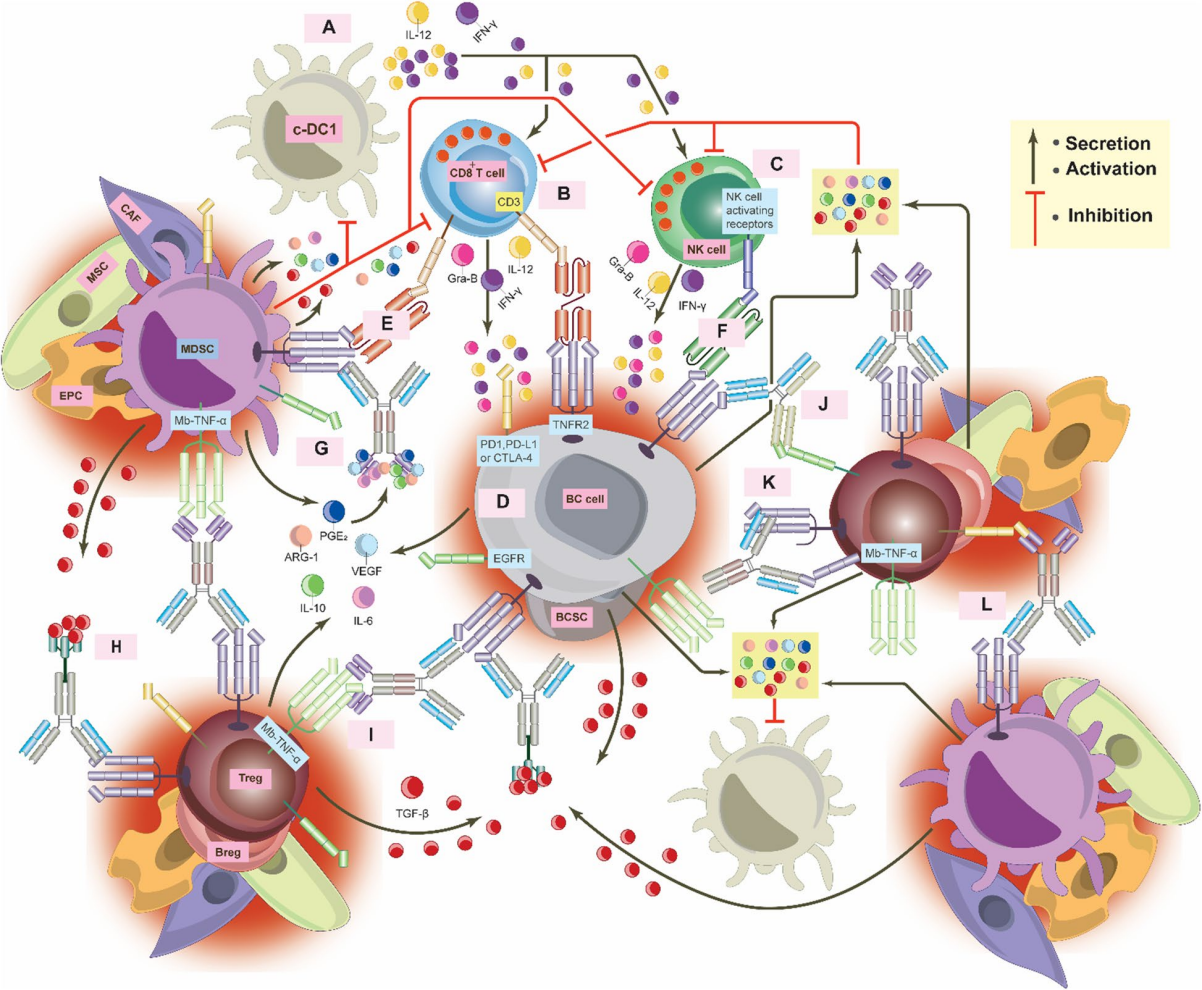
Anti-TNFR2 bs-Ab mechanism of action. **A.** DCs activate both CD8^+^ T cells and NK cells by TAA-MHC-1 presentation and secretion of IL-12 and IFN-γ. **B, C,** and **D.** Following the activation**,** CD8^+^ T cells and NK cells will attack and destroy BC cells by producing high quantities of IL-12, IFN-γ, and gra-B. BC cells and BCSCs, in turn, express EGFR, Mb-TNF-α, TNFR2, PD-1, PD-L1, and CTLA-4, and also produce IL-6, IL-10, PEG_2_, VEGF, TGF-β, and ARG-1, which suppress DCs, CD8^+^ T cells, and NK cells. Other immunosuppressive cells, including MDSCs, T-regs, B-regs, CAFs, EPCs, and MSCs in the BC TME also produce tremendous quantities of IL-6, IL-10, PEG_2_, VEGF, ARG-1, and TGF-β, as well as over-express TNFR2, PD-1, PD-L1, EGFR, Mb-TNF-α, and CTLA-4, leading to DC, CD8^+^ T cell, and NK cell suppression. **E.** T cell engager bs-Ab (TNFR2 × CD3ε) targeting CD3ε on CD8^+^ T cells and TNFR2 on BC cells and BCSCs, blocking the signals of TNFR2 and stimulating CD8^+^ T cells to kill tumors. **F.** NK cells engager bs-Ab (TNFR2 × NK cell activating receptors) targeting NK cell activating receptors (NCR1, NCR2/NKp44, NCR3/NKp30, NCR3/NKp46, NKG2D, and MICA/B) on NK cells and TNFR2 on BC cells and BCSCs blocking the signals of TNFR2 and stimulating NK cells to kill tumor. **G, I,** and **L.** bs-Ab targeting TNFR2 on TNFR2^+^ cells to hinder its signal as well as targeting CTLA-4, PD-1, PD-L1, Mb-TNF-α, ARG-1, PGE_2_ TGFβR2, VEGFR1,2, IL-6R, and IL-10R expressed by the same cells inhibit their signaling pathway. These bs-Ab capture TGF-β, VEGF, IL-6, IL-10, ARG-1, and PGE2, which in turn diminishes the immunosuppressive effect in BC TME. **K.** bs-Ab (TNFR2 × EGFR) targeting TNFR2 and EGFR on BC cells, BCSCs, and the immunosuppressive cells to suppress their signals and induce an effective anti-tumor immune response. **L.** Bs-Ab (TNFR2 × TNFR2) binding to one chain of the tri-TNFR2 complex to prevent clustering on the surface of BC cells, BCSCs, MDSCs, T-regs, B-regs, CAFs, EPCs, and MSCs. TNFR2, tumor necrosis factor receptor type two; Mb-TNF-α, membrane-bound tumor necrosis factor; Bs-Ab**,** bi-specific antibody; c-DC1, type 1 conventional DCs; BC, breast cancer; BCSCs, breast cancer stem cells; TAA, tumor associated antigen; MHC-1, major histocompatibility complex class one; IL-6, interleukin 6; IL-10, interleukin 10; IL-12, interleukin 12; IFN-γ, interferon gamma; gra-B, granzyme B; EGFR, epidermal growth factor receptor; PD-1, programmed cell death 1; PD-L1, programmed cell death ligand 1; CTLA-4, cytotoxic T lymphocyte antigen 4; TGF-β, transforming-growth factor β; VEGF, vascular endothelial growth factor; PEG_2_, prorstaglandin-E2; ARG-1, arginase-1; MDSCs, myeloid-derived suppressor cells; T-regs, T regulatory cells; B-regs, B regulatory cells, CAFs, cancer associated fibroblast; EPCs, endothelial progenitor cells; MSCs, mesenchymal stem cells; TME, tumor microenvironment; NCR1, natural cytotoxicity triggering receptor 1; NCR3, natural cytotoxicity triggering receptor 3; MICA and B, MHC class I chain-related polypeptide A and B; NKG2D, natural killer group 2-member D; NKp30, natural killer protein 30; NKp44, natural killer protein 44; NKp46, natural killer protein 46

In order for TNFR2 to be fully activated, three molecules (A, B, and C) need to cluster together on the cell surface (Fig. 10) (Fig. 11) (Table 2). Therefore, bs-Abs that can prevent cluster formation or its association with Mb-TNF-α could be highly effective in inhibiting the impact of TNFR2 activation in the BC TME (Fig. 11). Inhibiting TNFR2 in this manner could have a significant impact on the TME, by suppressing cancer cell growth, limiting angiogenesis, preventing metastasis, restoring TAA presentation by DC1s, and enhancing NK cell and CD8^+^ T cell function (Fig. 11). The versatility of TNFR2 bs-Abs allows for a wide range of functions, providing superior inhibitory effects and more selective inhibition when compared to traditional monoclonal antibody combinations (Fig. 10).

**Table 2:** Different proposed bs-Abs for targeting TNFR2 and different immunosuppressive molecules and receptors

Bs-Abs	Target antigen	Cell type	Function	Format
TNFR2A × TNFR2B, TNFR2A × TNFR2C and TNFR2B × TNFR2C	TNFR2	BC cells, BCSCs, MDSCs, T-regs, B-regs, CAFs, MCSs, EPCs	Block the signal of TNFR2. Prevent clustering of TNFR2 units	1 + 1
Mb-TNFαA × Mb-TNFαB, Mb-TNFαA × Mb-TNFαC and Mb-TNFαB × Mb-TNFαC	TNFR2 and Mb-TNFα	BC cells, BCSCs, MDSCs, T-regs, B-regs, CAFs, MCSs, EPCs	Block the interaction between membrane-bound-TNFR2 and Mb-TNFα	1 + 1
TNFR2 × EGFR	TNFR2 and EGFR	BC cells, BCSCs, MDSCs, T-regs, B-regs, CAFs, MCSs, EPCs	Block the signals of TNFR2 and EGFR	1 + 1
TNFR2 × CTLA-4	TNFR2 and CTLA-4	BC cells, BCSCs, MDSCs, T-regs, B-regs, CAFs, MCSs, EPCs	Block the signals of TNFR2 and CTLA-4	1 + 1
TNFR2 × PD-L1/PD-1	TNFR2 and PD-L1	BC cells, BCSCs, MDSCs, T-regs, B-regs, CAFs, MCSs, EPCs	Block the signals of TNFR2 and PD-L1/PD-1	1 + 1
TNFR2 × VEGFR	TNFR2 and VEGFR	BC cells, BCSCs, MDSCs, T-regs, B-regs, CAFs, MCSs, EPCs	Block the signals of TNFR2 and block the binding of VEGF (trap)	1 + 1
TNFR2 × CD20	TNFR2 and CD20	BC cells, BCSCs, MDSCs, T-regs, B-regs, CAFs, MCSs, EPCs	Block the signals of TNFR2 and CD20	1 + 1
TNFR2 × TGFβR2	TNFR2 and TGFβR2	BC cells, BCSCs, MDSCs, T-regs, B-regs, CAFs, MCSs, EPCs	Block the signals of TNFR2 and block the binding of TGFβ (trap)	1 + 1
TNFR2 × CD3ε	TNFR2 and CD3ε	BC cells, CD8^+^ T cells	Block the signals of TNFR2 and activate CD8^+^ T cells (T cell engager)	1 + 1
TNFR2 × NK cell activating receptors	TNFR2 and NK cell activating receptors	NK cells, BC cells, BCSCs, MDSCs, T-regs, B-regs, CAFs, MCSs, EPCs	Block the signals of TNFR2 and activate NK cells (NK cell engager)	1 + 1
TNFR2 × LAG-3	TNFR2 and LAG-3	BC cells, BCSCs, MDSCs, T-regs, B-regs, CAFs, MCSs, EPCs	Block the signals of TNFR2 and LAG-3	1 + 1
TNFR2 × CXCR-3	TNFR2 and CXCR-3	BC cells, BCSCs, MDSCs, T-regs, B-regs, CAFs, MCSs, EPCs	Block the signals of TNFR2 and block the binding of CXCR-3 ligands	1 + 1
TNFR2 × CXCR-4	TNFR2 and CXCR-4	BC cells, BCSCs, MDSCs, T-regs, B-regs, CAFs, MCSs, EPCs	Block the signals of TNFR2 and block the binding of CXCL-12	1 + 1
TNFR2 × IL-4R	TNFR2 and IL-4R	BC cells, BCSCs, MDSCs, T-regs, B-regs, CAFs, MCSs, EPCs	Block the signals of TNFR2 and block the binding of IL-4 (trap)	1 + 1
TNFR2 × IL-6R	TNFR2 and IL-6R	BC cells, BCSCs, MDSCs, T-regs, B-regs, CAFs, MCSs, EPCs	Block the signals of TNFR2 and block the binding of IL-6 (trap)	1 + 1
TNFR2 × IL-10R	TNFR2 and IL-10R	BC cells, BCSCs, MDSCs, T-regs, B-regs, CAFs, MCSs, EPCs	Block the signals of TNFR2 and block the binding of IL-10 (trap)	1 + 1
TNFR2 × E2 receptors	TNFR2 and E2 receptors	BC cells	Block the signals of TNFR2 and block the binding of PEG_2_	1 + 1

TNFR2, tumor necrosis factor receptor type two; Mb-TNF-α, membrane-bound tumor necrosis factor; Bs-Ab, bi-specific antibody; BC, breast cancer; BCSCs, breast cancer stem cells; IL-6, interleukin 6; IL-10, interleukin 10; IFN-γ, interferon gamma; EGFR, epidermal growth factor receptor; PD-1, programmed cell death 1; PD-L1, programmed cell death ligand 1; CTLA-4, cytotoxic T lymphocyte antigen 4; TGF-β, transforming-growth factor β; VEGF, vascular endothelial growth factor; PEG_2_, prorstaglandin-E2; MDSCs, myeloid-derived suppressor cells; T-regs, T regulatory cells; B-regs, B regulatory cells, CAFs, cancer associated fibroblast; EPCs, endothelial progenitor cells; MSCs, mesenchymal stem cells; IL-4R, IL-4 receptor; IL-6R, IL-6 receptor; IL-10R, IL-10 receptor, VEGFR, VEGF receptor; CXCL-12, C-X-C motif chemokine receptor 3; CXCR4, C-X-C motif chemokine receptor 4; LAG3, lymphocyte activation gene 3

When designing bs-Abs it is crucial to consider epitope locations, target choice, distance between binding sites, affinities, molecular size, valencies, the presence or absence of an Fc region or Fc-mediated effector functions, and flexibility [318]. Some bs-Abs have been specifically engineered to support chimeric antigen receptor (CAR) T cells without interacting with normal T cells. Bs-Abs have been made to function as T cell engagers for CAR T cells, serving as adaptors between CAR T cells and cancer cells. These have been used to great effect when adoptively transferring T cells with bs-Abs against cancer antigens in the synthetic agonistic receptor (SAR)-T cell system [318]. The utilization of bs-Abs antibodies for linking effector immune cells and cancer target cells can also be extended to NK cells. The HER2-S-Fab bs-Ab demonstrated significant efficacy in inhibiting BC both in vivo and in vitro [328]. Additionally, it was able to direct and link NK cells to BC cells that overexpressed HER2 receptors, thereby enhancing the anti-tumor immune response [328]. In addition to this, another bs-Ab was employed to establish a connection between NK cells and tumor cells that exhibited high levels of EpCAM. The CD16 × EpCAM bs-Ab effectively targeted and activated NK cells, resulting in increased NK cell proliferation and enhanced ADCC function [329]. These findings indicate that patients with EpCAM overexpression can bs-Abs to great effect. In a similar manner, ULBP2-BB4, a bispecific antibody that links tumor CD138 with the NK cell receptor NKG2D, was able to activate NK cells [330], leading to an increase in the production of IFN-γ and the destruction of cancer cells [330]. Likewise, muc1-Bi-1 and muc1-Bi-2, two bs-Abs, have demonstrated remarkable efficacy in specifically targeting MUC-1 on cancer cells and connecting it to CD16 on NK cells [331]. This bs-Abs effectively attracted NK cells to cancer cells that had high levels of MUC-1, which in turn increased NK cells' ability to kill the cancer cells and also boosted their production of cytokines, both in in vivo and in vitro [331]. These data suggest that bs-Abs can be used to connect CAR T cells and NK cells to tumor cells, enhancing their anti-tumor immune response. While these pre-clinical and clinical results are promising, additional research is required to understand the true potential of using TNFR2 bs-Abs in the BC TME.

#### *CAR *NK cells and *CAR* T cells for TNFR2 targeting

**Fig. 12:**
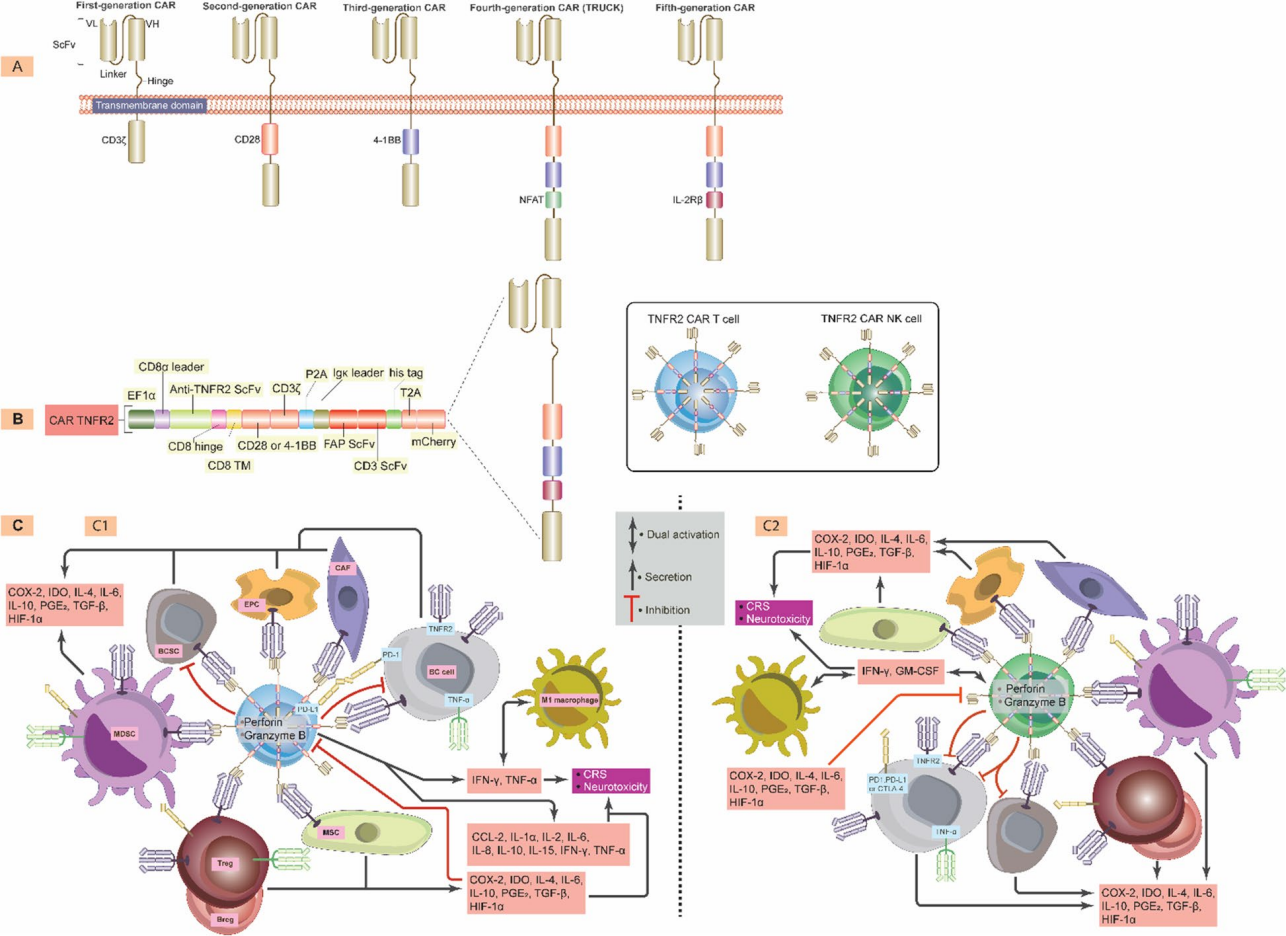
TNRT2-specific CAR T and CAR NK cells. **A.** Schematic representing the structure of the five generations of CARs. **B.** The proposed structure of TNFR2-specific CAR, including TNFR2 CAR T cells (blue) and TNFR2 CAR NK cells (green). **C.** The proposed mechanism of action: **C1.** First, TNFR2 CAR T cells will target TNFR2 on BC cells, BCSCs, MDSCs, T-regs, B-regs, CAFs, EPCs, and MSCs to deactivate TNFR2 to hinder its signal. These can produce the immunosuppressive mediators COX-2/PGE_2_, IDO, IL-4, IL-6, TGF-β, and HIF-1α which can inhibit TNFR2-specific CAR T cells and the effector CD8^+^ T cells. When targeting TNFR2 on BC cells and BCSCs, it will be able to block the interaction between Mb-TNF-α on activating cells (which could be any cell that over-expresses Mb-TNF-α in the BC TME) and TNFR2 on cancer cells, and at the same time, TNFR2-specific CAR T cells will produce perforin and granzyme B to kill the BC cells. **C2.** Second, TNFR2-specific CAR NK cells will fulfill the same functions as TNFR2-specific CAR T cells. While TNFR2-specific CAR T cells secrete CCL-2, IL-1α, IL-2, IL-6, IL-8, IL-10, IL-15, IFN-γ, and TNF-α, TNFR2-specific CAR NK cells will secrete IFN-γ and GM-CSF. TNFR2, tumor necrosis factor receptor type two; Mb-TNF-α, membrane-bound tumor necrosis factor; BC, breast cancer; BCSCs, breast cancer stem cells; IL-1α, interleukin 1 alpha; IL-2, interleukin 2; IL-4, interleukin 4; IL-6, IL-8, interleukin 8; IL-10, interleukin 10; IFN-γ, interferon-gamma; GM-CSF, granulocyte–macrophage colony stimulating factor; HIF-1α, hypoxia inducible factor 1 alpha; COX-2, cyclooxygenase-2; TGF-β, transforming-growth factor β; IDO, indoleamine 2,3-dioxygenase; PEG_2_, prostaglandin-E2; ScFv, single-chain variable fragment; MDSCs, myeloid-derived suppressor cells; T-regs, T regulatory cells; B-regs, B regulatory cells, CAFs, cancer associated fibroblast; EPCs, endothelial progenitor cells; MSCs, mesenchymal stem cells; TME, tumor microenvironment; CRS, cytokine release syndrome

CAR T cells contain receptors that have been engineered to bind to a particular antigen. This binding triggers a signaling cascade that activates T cells, leading to increased cell proliferation, enhanced cytotoxic activity, and elevated cytokine production. ultimately leading to the destruction of cancer cells [332]. The extracellular-antigen recognition domain of CAR T cells consists of an antibody single-chain variable fragment (ScFv), while its intracellular signaling domain typically includes a peptide or protein associated with CD3ζ of the TCR receptor (Fig. 12A) [332]. In fact, CAR T cells often include CD28 or 4-1BB/CD137, which serve as co-stimulatory proteins [333]. The primary source of approved CAR T cells is typically autologous T cells from the patients. This approach effectively prevents cell rejection by the patient's body [332, 333]. CAR T cells have shown efficacy in combating several types of blood cancers [334], while their effectiveness in treating solid tumors is currently limited [335]. Various receptors, such as CD19, CD2, CD20, CD22, CD33, CD123, CD138, CD171, HER2, PD-1, E7, CTLA-4, Igκ, glypican 3, LewisY antigen, fibroblast activation protein α (FAP-α), ROR1, protein claudin-6 (CLDN6), EGFRvIII, BCMA, IL-13Rα, MUC-1, GD2, CEA, VEGR, ICAM-1 and prostate-specific membrane antigen (PSMA) have been identified as targets for CAR T cells in multiple types of cancers [332, 334–338]. Currently, there is no evidence to suggest the development of engineered TNFR2 specific CAR T cells. However, there are some studies reporting the effectiveness of CAR T cells against Mb-TNF-α. A study conducted by Hongping et al*.* demonstrated that Mb-TNF-α-specific CAR T cells had potent cytotoxicity Mb-TNF-α-overexpressing BC cells [339]. This was evident from the substantial increase in IFN-γ and IL-2 levels observed both in vivo and in vitro [339]. Although, the study also found that overexpression of Mb-TNF-α on BC cells led to a significant increase in the production of surface PD-L1 through the TNFR2/p38/NF-κB-p65/Akt pathway; this ultimately hindered the effectiveness of PD-1 expressing CAR T cells in eliminating cancer. However, antibodies targeting PD-1 restored CAR T cell function [339]. In addition, CAR T cells targeting claudin 18.2 (CLDN-18.2) and expressing CXCR4 were discovered to have increased infiltration and improved effectiveness in response to CAFs-mediated significant CXCL-12 secretion [340]. CXCR4^+^ CAR T cells in the TME caused the depletion of CXCL-12, resulting in a reduction of cytokine secretion, including TNF-α, IL-6, and IL-17. As a result, MDSC recruitment was reduced through the inhibition of STAT3/NF-κB/CXCL-12 in CAFs [340]. On the other hand, the increased expression of CXCR4 on T-regs, B-regs, and MDSCs through TNFR2 could result in a higher ratio of these cells compared to CXCR4^+^ CAR T cells. As a result, they would effectively outcompete CXCR4^+^ CAR T cells for the stromal CXCL-12, leading to a significant reduction in the number of CXCR4^+^ CAR T cells in the TME. It is possible that CAR T cells targeting TNFR2 may have a large potential for cancer therapy, in a similar manner as observed for Mb-TNF-α (Fig. 12B, C).

By targeting cancer cells, T-regs, MDSCs, and B-regs, as well as other immunosuppressive cells using TNFR2-specific CAR T cells, the negative impact of these cells in the BC TME can be significantly diminished and an efficient anti-tumor immune response can be reestablished. Manriquez et al. have shown that there was an increase in TNFR2 expression in CAR T-19 cells, which was associated with a defective phenotype [341]. Nevertheless, deletion of TNFR2 using CRISPR-Cas9 resulted in notable proliferation, elevated cytotoxicity, and improved anti-tumor efficacy of CAR T-19 cells [341]. This suggests that TNFR2 expression might be associated with loss of function and ultimately cell exhaustion in CAR T cells. In fact, regardless of its origins, TNFR2 seems to be consequential, since all TNFR2^+^ cells within the TME of BC are capable of transmitting signals of exhaustion to CD8^+^ T cells and CAR T cells (Fig. 9C). Functional investigations are necessary to address the issue of TNFR2 expression on CAR T cells and determine if combining TNFR2-negative CAR T cells with monoclonal or bs-Abs could impact the BC TME. Various factors in the BC TME have the potential to affect the function of TNFR2^+^ CAR T cells negatively which are part from the ISG. These factors include COX-2, IDO, IL-4, IL-6, IL-10, PGE_2_, TGF-β, and HIF-1α, all of which are linked to the activation of the TNFR2 signaling pathway (Fig. 12C). In particular, TGF-β’s presence in the TME can decrease CXCR-3, diminishing cancer immune surveillance and boosting collagen production (a key component of the ECM), ultimately impeding CAR T cells homing [337, 342]. This issue was resolved, however, through the development of TGF-β DNR, TGF-βR-4-1BB, and TGF-β-specific CAR T cells, which disrupt TGF-β’s intercellular signaling pathway [342]. It was also discovered that a short peptide expressed by CAR T cells could disrupt the protein kinase A (PKA, PGE_2_ downstream target protein) signals, which in turn increases CXCR-3 expression leading to an increase in CAR T cell infiltration into active tumor regions and enhanced tumor cell killing potential [342]. Hypoxia and HIF-1α impose various physical and metabolic challenges that favor a tumor immunosuppressive environment that could also be supported by the TNFR2 signaling pathway. Thus, a CAR T cell was developed by fusing the CAR scaffold with the HIF-1α oxygen-sensitive subdomain to take advantage of the TME hypoxia to further infiltrate cancer effectively [343]. Continued research is being conducted to develop advanced CAR T cells that can effectively address the issue of hypoxia in the TME and overcome the metabolic challenges. It appears that CAR NK cells have a comparable function to CAR T cells within the BC TME. Research has shown that the presence of TGF-β in the TME can decrease the levels of NKG2D receptors in NK cells [342]. This reduction allows tumor cells and immunosuppressive cells like MDSCs, which produce NKG2D ligands, to evade the immune system. It was observed that NKG2D CAR NK cells effectively targeted and eliminated MDSCs, even in the presence of TGF-β [342]. Nevertheless, the immunosuppressive TME mediated by the TNFR2 signaling pathway may also be relevant for CAR NK cells. Therefore, the utilization of CAR NK cells that specifically target TNFR2 could potentially mitigate the detrimental impact of TNFR2 on the BC TME. Indeed, CAR NK cells have the ability to eliminate cancers through both CAR-dependent and CAR-independent mechanisms [344]. This, in turn, could enhance the TNFR2 CAR NK cell functions by neutralizing TNFR2 on cancer cells, T-regs, MDSCs, and B-regs, as well as other immunosuppressive cells, and at the same time killing cells via cytotoxic mediators. When comparing CAR NK cells with CAR T cells, it is evident that the incidence of cytokine release syndrome (CRS) and neurotoxicity is significantly lower in CAR NK cells (Fig. 12C) [344]. The cytokine profile of CAR NK cells differs from that of CAR T cells, which leads to SRS and neurotoxicity. CAR NK cells primarily produce IFN-γ and GM-CSF, while CAR T cells produce CCL-2, IL-1α, IL-2, IL-6, IL-8, IL-10, IL-15, and TNF-α (Fig. 12C) [344]. Similarly, the design of CAR T cells against a single antigen may impact their function due to TME heterogeneity. On the other hand, increasing CAR T cell specificities could result in an on-target, off-cancer toxic effect, thus leading to fatal clinical complications [342]. Selecting TNFR2 as a unique antigen for BC cells for CAR T cell targeting could reduce this effect. While CAR NK cells could offer an “off-the-shelf” approach, they could be used universally across various subjects without the need for personalized customization [344]. Collectively, when designing receptor-engineered TNFR2 CAR T cells and TNFR2 CAR NK cells, all the previous conditions should be taken into consideration. In addition, the possibility for the TNFR2 gene to acquire mutations should be considered for successful application in the clinic, alongside taking safety concerns regularly present in the development process into account.

## Concluding remarks and future directions

TNFR2 is crucial in various aspects of BC, including development, evasion of anti-tumor immune response, resistance to drugs, resistance to apoptosis, metastasis, and growth. Many cell types, including T-regs, MDSCs, B-regs, NK cells, CAFs, EPCs, and MSCs, as well as BC cells and BCSCs, may exhibit aberrant TNFR2 expression, which in turn enhances their immunosuppressive activity. This abnormal expression is associated with the activation of several signaling pathways, which can result in the suppression of NK cells, CD8^+^ T cells, M1 macrophages, and DC1s through the production of several immunosuppressive mediators. This highlights the potential of TNFR2 as a targeting marker for immunotherapy. Blocking the TNFR2 signaling pathway in the BC TME could provide a therapeutic opportunity in the battle against BC. An alternative strategy involves targeting the TNFR2 expression on the surface of BC cells, BCSCs, and immunosuppressive cells. This has the potential to decrease their immunosuppressive functions and improve the efficacy of different BC drugs and cellular therapies, such as immune checkpoint inhibitors and adoptive T cell and NK cell therapies. An issue that arises from the abnormal activation of TNFR2 is the creation of an immunosuppressive gradient (ISG) due to the activation of the immunosuppressive cells. The ISG has the capacity to induce global immunosuppression, impacting a wide range of effector cells such as NK cells, CD8^+^ T cells, M1 macrophages, and c-DC1s. One potential solution for this ISG could involve minimizing its presence in the BC TME. Therefore, it is crucial to conduct studies to identify methods that shift the ISG in favor of an anti-tumor immune response.

Although there are a well-established TNFR2 antagonistic antibodies in the pre-clinical settings but their role in clinical trials still very limited. A better designing and full understanding of anti-TNFR2 antibodies could facilitate their transition to the clinics to combat BC.

Given the fact that TNFR2 is expressed abundantly on immunosuppressive cells, dominant TNFR2 bi-Abs and TNFR2-specific CAR T cells and TNFR2-specific NK cells can be used to mitigate the deleterious effects of TNFR2. Safety precautions such cellular toxicity should be put in consideration when designing these approaches.

## Abbreviations

BC: Breast cancer
TME: Tumor Microenvironment
TNFR2: Tumor Necrosis Factor (TNF) Receptor 2
Mb-TNF-α: Membrane-bound tumor necrosis factor
T-Regs: Regulatory T Cells
MDSCs: Myeloid-Derived Suppressor Cells
TRADD: TNFR1-Associated Death Domain
Trafs: TNFR-Associated Factors
Crds: Cysteine-Rich Domains
PLAD: Pre-Ligand Assembly Domain
THD: TNF-Α Homology Domain
TACE: TNF-Α-Converting Enzyme
ADAM17: Disintegrin or Metalloproteinase Domain-Containing 17
T2bs-C: TRAF2-Binding Site C
T2bs-N: TRAF2's Binding Site N
cIAP: Cellular Inhibitor of Apoptosis
SHARPIN: Shank-Associated RH Domain-Interacting Protein
HOIL-1-K63-Ub: Heme-Oxidized IRP2 Ubiquitin Ligase 1
HOIP-M1-Ub: HOIL-1-Interacting Protein
LUBAC: Linear Ubiquitin Chain Assembly Complex
MEKK: MAP Kinase/ERK Kinase
TAK1: Transforming Growth Factor-Activated Kinase 1
TAP1: TAK1-Binding Protein Complex
IKKs: Iκb Kinases
PI3K: Phosphoinositide 3-Kinases
CYLD: Ubiquitin-Deubiquitinase Cylindromatosis
PFK-2: Phosphofructokinase-2
PFKFB2, 3: 6-Phosophofrcto-2-Kinase/Fructose-2, 6-Biphosphatase 3
mTOR: Mammalian (Or Mechanistic) Target of Rapamycin
PTEN: Phosphatase And Tensin Homolog
VEGF: Vascular Endothelial Growth Factor
VEGFR2: VEGF Receptor 2
CCL-2: Chemokine (C–C) Motif Ligand 2
CXCL-1: C-X-C Motif Chemokine Ligand 1
M-CSF: Macrophage Colony Stimulating Factor
ETK: Epithelial Tyrosine Kinase
BMX: Marrow X-Linked Kinase
EMT: Epithelial-Mesenchymal Transition
MMP: Matrix Metalloproteinase
FOXO3a: Forkhead Box O3a
JNK: C-Jun N-Terminal Kinase
ERK: Extracellular Signal-Regulated Kinase
C-MYC: Cellular Myelocytomatosis
Stats: Signal Transducer and Activator Of Transcriptions
AP-1: Activator Protein-1
TET: Ten-Eleven Translocation
5mc: 5-Methylcytosine
5hmc: 5-Hydroxymethycytosine
5fc: 5-Formylcytosine
5cac: 5-Carboxylcytosine
IBC: Inflammatory BC
COX-2: Cyclooxygenase-2
PMA: Phorbol-12-Myristate-13-Acetate
PGE2: Prostaglandin E2
NDRG-2: N-MYC Downstream-Regulated Gene 2
CRP: C-Reactive Protein
TGF-Β: Transforming-Growth Factor Β
FAK: Phosphorylates Focal Adhesion Kinase
IL-10: Interleukin 10
TRADD: TNFR1-associated death domain
TWEAK: Tumor necrosis-like weak inducer of apoptosis
BAD: Bcl-2-associated agonist of cell death
pro-cas8: Pro-caspase 8
pro-cas10: Pro-caspase 10
Bcl-2: B cell lymphoma 2
Bcl-XL: B cell lymphoma extra-large
PTPRZ1: Receptor-type tyrosine-protein phosphatase ζ
EPHA4: Ephrin type-A receptor-4
HIF-1α: Hypoxia inducible factor 1 alpha
CXCR4: C-X-C chemokine receptor 4
HRE: Hypoxia response element
VHL: Von Hppel Lindau
PD-L1: Programmed cell death ligand 1
T6BD: TRAF 6 binding domain
PHD1: Prolyl-hydroxylase 1
FIH: Factor-inhibiting HIF-1α
VCAM-1: Vascular cell adhesion molecule-1
DNMTs: DNA methyl transferases
MAGEH1: Melanoma antigen family H1 gene
XCR1: X-C motif chemokine receptor 1
CCR: C–C chemokine receptor type
EGFR: Epidermal growth factor receptor
GLUT: Glucose transporter
α-KG: Alpha ketoglutarate
PKM2: Pyruvate kinase M2
ENO-1: Enolase-1
LDHα: Lactate dehydrogenase-alpha
IDO: Indoleamine 2,3-dioxygenase
c-TGF: Connective-tissue growth factor
pDGF: Platelet-derived growth factor
IGF-1: Insulin-like growth factor 1
FGF: Fibroblast growth factor
HGF: Hepatocyte growth factor
EGF: Epidermal growth factor
bFGF: Basic insulin-like growth factor
G-CSF: Granulocyte-colony stimulating factor
B-regs: B regulatory cells
CAFs: Cancer associated fibroblast
EPCs: Endothelial progenitor cells
MSCs: Mesenchymal stem cells
ECM: Extracellular matrix
MIC: MHC class I chain-related polypeptide
NKG2D: Natural killer group 2-member D
NKp: Natural killer protein
ARG-1: Arginase-1
iNOS: Inducible nitric oxide synthase
NCR1: Natural cytotoxicity triggering receptor
cPGES: Cytosolic PGE2 synthase
mPGES-1: Membrane-bound PGE2 synthase 1
TAVECs: Tumor-associated vascular endothelial cells
CADM-1: Cell adhesion molecule 1
c-DCs1: Type 1 conventional DCs
IRF8: Interferon regulatory factor family 8
ID2: Inhibitor of DNA binding 2
NFIL3: Nuclear factor interleukin-3
ZBTB46: Zinc-finger and BTB domain-containing-46
BATF3: Basic leucine zipper transcription factor ATF-like 3
EpCAM: Epithelial cellular adhesion molecule
BCMA: B cell maturation antigen
Bs-Abs: Bi-specific antibodies
CAR T cells: Chimeric antigen receptor T cells
ISG: Immune suppressive gradient
